# Organocatalytic synthetic route to esters and their application in hydrosilylation process

**DOI:** 10.1038/s41598-024-70036-y

**Published:** 2024-08-17

**Authors:** Aleksandra Mermela, Małgorzata Bołt, Aleksandra Mrzygłód, Patrycja Żak

**Affiliations:** 1 Faculty of Chemistry, Department of Organometallic Chemistry, Adam Mickiewicz University in Poznań, Uniwersytetu Poznańskiego 8, 61-614 Poznań, Poland; 2 Centre for Advanced Technologies, Uniwersytetu Poznańskiego 10, 61-614 Poznań, Poland

**Keywords:** Homogeneous catalysis, Metal-organic frameworks

## Abstract

A facile esterification of α,β-unsaturated aldehydes with alcohols has been developed for the synthesis of esters by using bulky *N*-heterocyclic (NHC) carbene as a metal-free and eco-friendly organocatalyst. This new protocol has been proved to be effective with a wide substrate scope, giving selective esters in yields greater than 84% under mild conditions. Moreover, proposed synthetic strategy enables modification of various types of silsesquioxanes (SQ) which cannot or are technically difficult to be carried out with known protocols. For the first time, a *one-pot* sequential esterification/hydrosilylation has been successfully carried out.

## Introduction

The ester functional group represents one of the most common structures that can be found in many drug molecules, natural compounds and building blocks of organic materials^1^. Esters are currently used in a wide range of industries e.g. in the pharmaceutical / medical^2,3^, fuel^4^, food^4,5^, or cosmetic^5^, and toiletry^3^ industries. In the field of medicine some esters have found application in dissolving human cholesterol gallstones^6^. These carbonyl compounds are also ingredients of biodiesel fuel which is a more ecofriendly alternative to fossil fuels^4^. Furthermore, compounds with an ester unit may be found in many substances without which we cannot even imagine everyday life e.g. chewing gums, soaps, deodorants or toothpaste (these products contain ester-based flavoring agents, fragrances)^4^. In view of the wide range of applications of this class of compounds, it is highly desirable to develop and improve synthetic procedures of their production.

**Figure 1:**
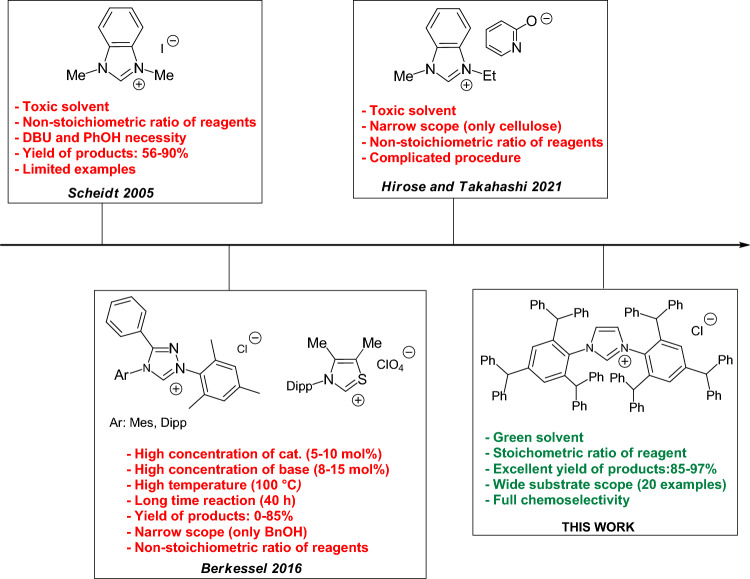
Known NHCs used in the reaction α,β-unsaturated aldehydes with alcohols and our concept.

There are many different methods for syntheses of esters. These compounds can be obtained e.g. by the reaction of alcohols and carboxylic acids^7^ or alcohols and ketenes^8^ or via sp^3^ C-H functionalization^9^. Nowadays, one of the most interesting methods for the ester production is based on the catalytic conversion of aldehydes into esters. Transition metal complexes are widely used as catalysts of this process^10–12^. In the 1980s, Murahashi et al. developed a method for converting alcohols and aldehydes to esters using a ruthenium catalyst. This process required high temperatures and led to low product yields^10,11^. In 2016, nickel complex with NHC ligands was reported to be used in this reaction and gave satisfactory synthesis results. However, in this synthetic pathway it is necessary to use non-ecologically friendly solvents and add substances that would increase the selectivity of the proces^12^. Moreover, the need to protect the natural environment and comply with the principles of green chemistry requires elimination of the use of catalysts introducing metals into the environment. An interesting alternative to metal catalysts proved to be organocatalysts. Currently NHC carbenes are commonly used as catalysts in reactions between aldehydes and alcohols^13–16^ (Fig. 1).

However, the methods proposed so far provided the desired products with unsatisfactorily yields^13–16^. Moreover, the known catalytic systems very often require the application of high temperature, excess of one of the substrates or a significant amount of NHC to achieve the intended effect. The number of different esters obtained by the conversion of aldehydes is also still truly small^13–16^. Therefore, although a number of research reports on the procedure for obtaining esters using the above-mentioned method have been published, additional study on this subject is necessary for economic and ecological reasons.

Among the large number of organocatalysts, bulky NHC carbenes deserve special attention e.g. because of their specific steric properties that significantly affect the selectivity of the reaction^17,18^. However, the currently available scientific literature lacks information on the use of bulky NHC ligand precursors as catalysts for conversion of aldehydes into esters.

Herein, we described an effective synthetic pathway for obtaining esters via reactions of alcohols and α,β-unsaturated aldehydes in the presence of bulky NHC carbene (Fig. 1). We also indicated the usefulness of the selected product by subjecting it to further modifications. Finally, we developed a new class of functionalized SQ derivatives containing ester moieties.

## Results and discussion

### Design and optimization of the reaction system

**Figure 2:**
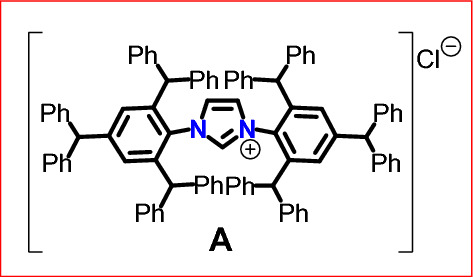
Structure of organocatalyst precursor** A**.

**Figure 3:**
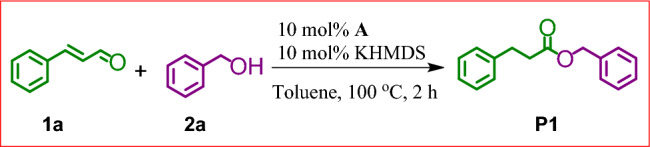
Esterification of cinnamaldehyde (**1a**) with benzyl alcohol (**2a**).

Our examination started with the synthesis of the organocatalyst precursor—NHC salt (**A**) containing sterically crowded groups localized at the terminal nitrogen atoms in imidazole rings, according to the methodology described in our previous paper^19^ (Fig. 2):

It has been proved that conversion of α,β-unsaturated aldehydes into saturated esters could occur in the presence of simple NHC precursors and a strong base^14^. For this reason we decided to apply similar conditions to the reaction of cinnamaldehyde (**1a**) and benzyl alcohol (**2a**) in the presence of the bulky NHC salt (**A**). The addition of equimolar ratio of reagents to a toluene solution of 10 mol% of **A** and 10 mol% of KHMDS at 100 °C, resulted in a complete conversion of the substrates after 2 h, as revealed by the GC–MS technique. The ^1^H NMR analysis of the reaction mixture showed a selective formation of the expected product **P1** in a quantitative yield (Fig. 3).

This achievement prompted us to continue our studies. A series of tests were performed to select optimal temperature, the concentration of NHC precursor and the appropriate solvent. All optimization tests were performed on the above model reaction. The results are summarized in Table 1.

**Table 1:** Optimization of the type of solvent, temperature and concentration of NHC precursor (A).

Entry	Solvent	T [°C]	[A] [mol%]	Time [h]	Conversion of 1a^d^ [%]	Yield of P1^d^ [%]
1	**Toluene**	100	10	2	100	100
2		80	10	5	100	100
3		60	10	10	95	95
4		40	10	48	75	75
5		100	5	18	100	100
6		80	5	24	98	98
7		60	5	24	90	90
8		40	5	24	25	25
9	**Acetone**	100	5	12	100	100
10		80	5	18	100	100
**11**		**60**	**5**	**24**	**98**	**98**
12		40	5	24	45	45
13		60	1	24	30	30
14^a^		60	5	24	99	99
15		60	-	72	0	0
16^b^		60	5	72	8	8
17^c^		60	5	72	3	3
18	**EGDA**	100	5	14	100	100
19		80	5	24	100	100
20		60	5	24	97	97
21		40	5	24	45	45
22	**MIBK**	100	5	16	100	100
23		80	5	24	99	99
24		60	5	24	95	95
25		40	5	24	40	40

Reaction conditions: [**1a**]:[**2a**] = 1:1, [**A**]**:**[**KHMDS**] = 1:1, argon.

^a^Freshly isolated carbene was used as a catalyst.

^b^Without KHMDS.

^c^Air atmosphere.

^d^Determined by GC–MS using dodecane as an internal standard.

Solvent screening showed that the process can be carried out both in toluene, ethylene glycol diacetate (EGDA), methyl isobutyl ketone (MIBK) and acetone. This is especially important in ecological aspects as these solvents, except toluene, are classified as green solvents^20^. Because of its density, boiling point and price, acetone was selected as the most suitable medium for the tested process. As presented in Table 1, the reaction that proceeded at 100 °C gave the same results as the ones run at 80 °C and 60 °C (Table 1, Entries 1–3, 5–7, 9–11, 18–20, 22–24). Further decrease in temperature led to a reduction in the product yield, even though the reaction time was increased to 48 h (Table 1, Entry 4). Next, the optimum concentrations of the NHC precursor and KHMDS were established. The best results were achieved by carrying out the process in the presence of 5 mol% A and 5 mol% base (Table 1, Entry 11). Lowering the concentration of NHC salt to 1 mol % resulted in a decrease in substrate conversion, despite the extension of the reaction time (Table 1, Entry 13). Carrying out the reaction without addition of KHMDS led to a conversion in trace amounts, which confirmed that a base is indispensable for the effective reaction course (Table 1, entry 16). Finally, we found that the atmosphere in which the process was conducted had a significant impact on the efficiency of the esterification reaction. This process must be carried out under dry argon, using standard Schlenk-line and vacuum techniques. Otherwise, the main product of the reaction practically does not form (Table 1, Entry 17). We did not observe significant differences between the process carried out in the presence of carbene generated in situ and the reaction catalyzed by freshly isolated free carbene (Table 1, Entry 11 vs Entry 14).

In the next step, the catalytic activity of the known, less sterically crowded precursors of NHC carbenes (IMes, IPr) was also verified. Surprisingly, these salts were found inactive, as the conversion of substrates reached only ca. 5% and no products of esterification were observed (Table 2).

**Table 2:** Optimization of the type of NHC precursor and base.

Entry	NHC·HCl	Base	Time [h]	Conversion of 1a^a^ [%]	Yield of P1^a^ [%]
1	A	KHMDS	24	98	98
2	A	DABCO	24	90	90
3	A	K_2_CO_3_	48	10	10
4	A	Cs_2_CO_3_	24	60	60
5	B	KHMDS	24	5	5
6	C	KHMDS	24	5	5
**NHC**·**HCl**					

Reaction conditions: [**1a**]:[**2a**] = 1:1, [**NHC**·**HCl**]**:**[**Base**] = 1:1, argon.

^a^Determined by GC–MS using dodecane as an internal standard.

### Scope of the reaction

**Figure 4:**
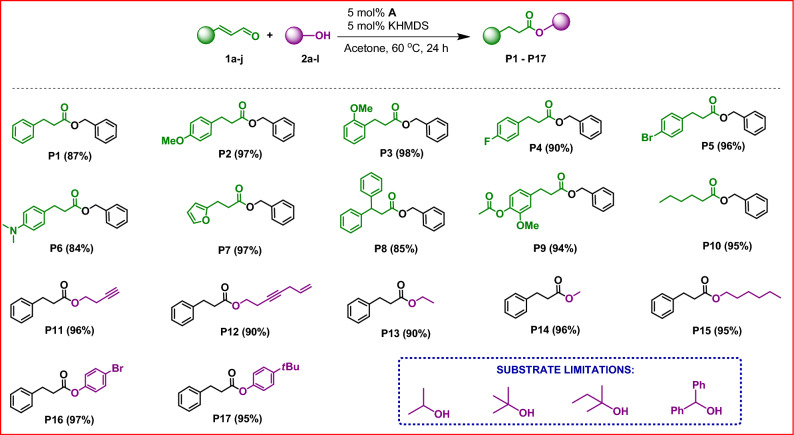
Substrate scope and overview of the obtained products. Isolated yields are given in parentheses.

Having the optimized conditions in hand, the range of substrates was extended to determine the versatility of the method. We tested the reactivity of commercially available α,β-unsaturated aldehydes (**1a-j**) toward selected benzyl (**2a**) and alkyl (**2b-f**, **2i-l**) alcohols as well as phenols (**2 g**, **2 h**) (Fig. 4).

In the first series of experiments, we checked the reactivity of a broad range of commercially available α,β-unsaturated aldehydes (**1a-j**) toward benzyl alcohol (**2a**), obtaining a wide variety of esters (**P1**-**P10**) in very good isolated yields with low organocatalyst concentrations and under mild reaction conditions. Fortunately, alkyl and aryl enals could be readily adopted in this protocol. No meaningful difference in the efficiency of the process for aryl aldehydes with both electron-withdrawing and electron-donating substituents was noted. Next, the catalytic properties of NHC salt **A** were evaluated in the esterification of a series of alcohols (**2a-j**) with cinnamaldehyde (**1a**). As shown in Fig. 4, the proposed method can be successfully applied to all alkyl- and aryl-substituted primary alcohols. Disappointingly, secondary and tertiary alcohols were found unsuitable for our catalytic system. When we used these compounds, their conversions were below 10%. The alcohols having unsaturated bonds in their structures were also applied in the reactions with **1a** and exhibited high activity leading to desired products (**P11** and **P12**). This opens up the possibility of synthesizing esters that may contain groups susceptible to further modification. Analogously, we carried out experiments using phenols instead of alcohols. As a result of the reactions, we observed the formation of esters, as confirmed by the analysis of their isolates from the post-reaction mixture (**P16**, **P17**). In this instance, as in other studies reported by our research group^17^, the course of the reaction depended on the type of the organocatalyst used. A significant steric hindrance promotes intramolecular proton transfer, leading to the formation of an ester rather than an SMA product (See section: Mechanistic studies). We isolated all products (**P1**-**P17**) in order to develop a universal method for their separation from the reaction mixture.

**Figure 5:**
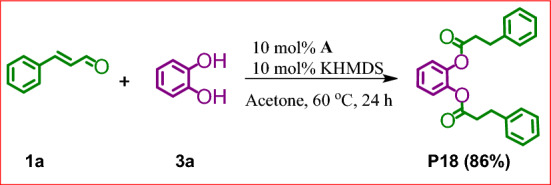
Bis-esterification of cinnamaldehyde (**1a**) with catechol (**3a**).

In the optimized reaction systems, we have also checked the possibility of multiesters formation. Relevant tests were performed using cinnamaldehyde (**1a**) and catechol (**3a**) at the molar ratio 2:1 (Fig. 5).

To our great satisfaction, the reaction led to the expected disubstituted derivative and product **P18** was isolated in a pure form in 86% yield. It is worth mentioning that we performed an additional reaction starting from equimolar amounts of reagents **1a** and **3a** in an attempt to achieve the selective esterification of only one hydroxyl group of **3a**. Unfortunately, this trial gave mixtures of mono- and disubstituted product at the 6:4 ratio. Decrease in the reaction temperature from 80 to 25 °C or addition of diol in small portions did not improve this selectivity.

**Figure 6:**
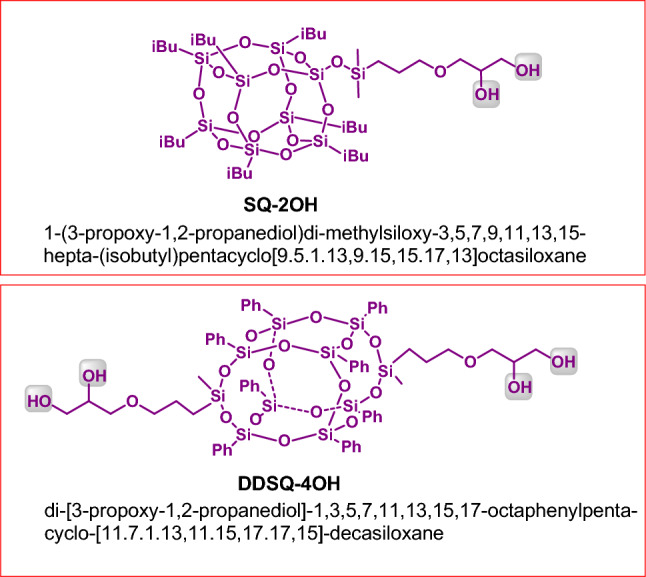
Structures of SQs used as substrate.

To highlight the utility of our procedure for the coupling between polyols and enals, we conducted experiments with the use of two different types of SQs depicted below (Fig. 6). We turned our attention to functionalization of compounds of this kind because, according to our knowledge, there are no literature reports on the functionalization of SQs containing hydroxyl groups with enals. Moreover, the materials based on SQs have the unique properties determining the directions of their versatile applications^21^, Particularly attractive is the possibility of using them in diverse areas of medicine, e.g. as drug delivery platforms to specific target sites^22–30^ or as the functional group carriers in photodynamic therapy and bioimaging^31,32^.

**Figure 7:**
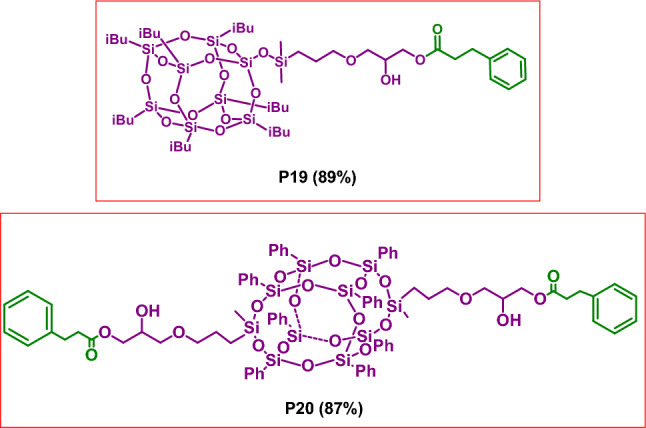
Structures of the obtained ester moieties containing SQs.

Finally, we obtained two novel hybrid materials of structures depicted in Fig. 7. We also made attempts to functionalize the other hydroxyl group(s) in the products **P19** and **P20**, at the secondary carbon atoms. In order to do this, we run the reaction of **SQ-2OH** using twofold excess of **1a** and the reaction of **DDSQ-4OH** using fourfold excess of **1a**. No expected products were obtained, which is consistent with the method limitation given in Fig. 4

### Application of selected product in hydrosilylation process

**Figure 8:**
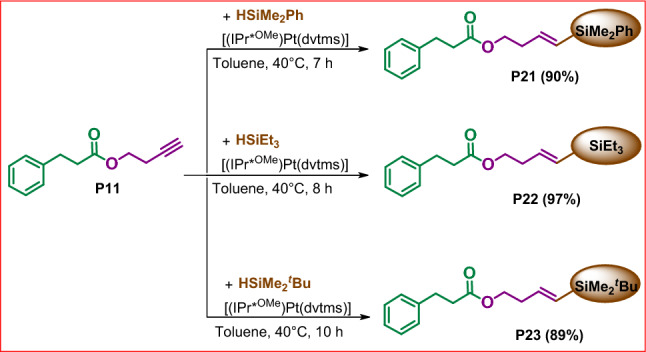
Usability of **P11** in hydrosilylation process. Isolated yields are given in parentheses.

Next, we have focused on potential applicability of the selected material. For further studies, we chose derivative **P11** containing a functional group susceptible to subsequent modifications. This choice permitted designing compounds that are excellent synthons for the synthesis of complex molecules of well-defined structures and interesting properties. Thus, **P11** bearing terminal unsaturated C≡C bond was verified in hydrosilylation processes (Fig. 8).

The proposed transformation was carried out under optimal conditions for terminal alkenes, established in our previous work^33^. As a result, we obtained a variety of organic derivatives with good to excellent isolated yields, ranging from 89 to 97%. It should be emphasized that in each reaction we observed a quantitative conversion of reagents. In the reaction systems proposed, neither side products nor other isomers of products **P21-P23** were observed to form.

**Figure 9:**
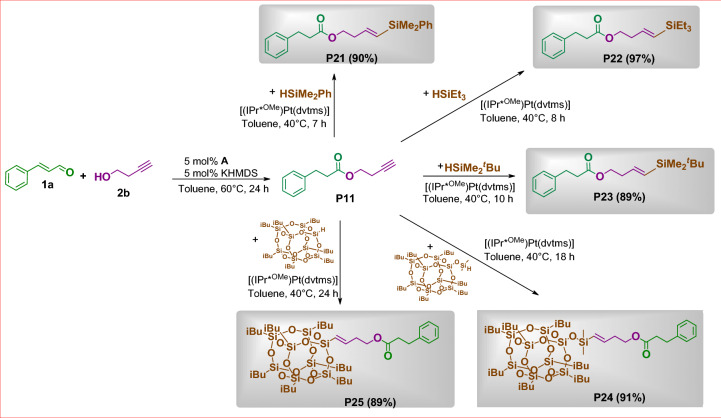
One-step synthesis of **P21**-**P25.** The isolated yields are given in parentheses.

The successful functionalization of **P11** prompted us to probe the feasibility of a one-pot procedure leading to products **P21**-**P25**. To accomplish this goal, we performed esterifications of **1a** with **2b** and subsequently use the obtained derivative **P11** in transformations depicted in Fig. 9.

### The preparative scale of synthesis

In the next step, we conducted a larger number of tests to reliably determine the scalability of the described method. The results, which are summarized in Table 3, indicated that the proposed methodology has a significant application potential. The conducted tests have clearly demonstrated that the described method allows the production of esters on a large scale, while maintaining high efficiency. This synthetic pathway permitted reduction of the amount of catalyst by half, while the amount of base by three times when compared to the amounts needed in the previously described methods, and does not require any excess of substrates. Additionally, what is important from the environmental perspective, only small amounts of the green solvent-acetone-are needed, rather than harmful toluene.

**Table 3:** Scaled-up synthesis of P1.


Entry	Amount of 1a [mg]	Amount of 2a [mg]	Conv. of 1a [%]^a^	Yield of P1 [%]^[a]^
1	21	17	100	100
2	63	52	100	98
3	528	432	98	90
4	1056	864	90	85

Reaction conditions: [**1a**]:[**2a**] = 1:1, [**A**]**:**[**KHMDS**] = 1:1, argon.

^a^Determined by GC–MS using dodecane as an internal standard.

In accordance with the standard practice of our laboratory, all catalytic tests were repeated three times and the obtained results indicated a high reproducibility of the method. To better verify the reproducibility of yields and reaction conditions, the model reaction was repeated five times. The standard deviation for the obtained yield values was calculated to be around 2. The results of the statistical analysis demonstrate good reproducibility and reliability of the described method (See ESI).

### Mechanistic studies

**Figure 10:**
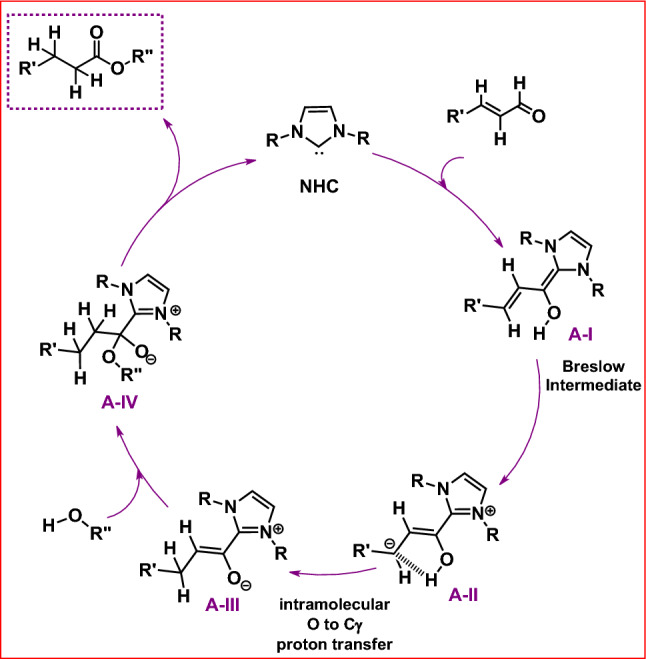
Proposed mechanism of esterification reaction.

Finally, based on our research of thioester synthesis from α,β-unsaturated aldehydes and alcohols catalyzed by bulky NHC^16^ and the deuterium-labelling experiment (See ESI), the following mechanism of this process is proposed (Fig. 10).

The reaction mechanism commences from the free carbene (NHC), which undergoes the reaction with α,β-unsaturated aldehyde. This reaction can lead to Breslow intermediate (**A-I**), which remains in equilibrium with its homoenolate form (**A-II**). ^13,34,35^ In the next step a direct proton transfer between the hydroxyl group and γ carbon atom can occur. ^36–38^ In this case there is no need to use alcohol excess or any additive, facilitating proton transfer, which was inevitable in the earlier developed systems. Finally, intermediate **A-III** reacts with alcohol to form the reaction product after the imidazole elimination and catalyst regeneration.

### Importance of the developed method

Our studies have brought about an efficient and eco-friendly synthetic pathway for obtaining esters which are important and valuable compounds in modern organic chemistry. In our opinion, the presented results are very attractive from the cognitive, economic and ecological points of view and the procedure proposed is a metal-free alternative to the hitherto applied catalytic methods described in literature. As shown below the system described and optimized proved to be very attractive when compared to the known organocatalytic protocols for obtaining esters. To make the comparison credible, we based it on the results obtained using similar reagents, i.e. α,β-unsaturated aldehyde and alkyl alcohol (Table 4).

**Table 4:** Comparison of the proposed method with the best known organocatalytic protocols.


Entry	NHC·HCl: [mol%]	Base [mol%]	Ratio S_1_ :S_2_	T [°C]	t [h]	Yield [%]^a^
1	**A** [5 mol%]	KHMDS [5 mol%]	1:1	60	24	100
2	**D** [10 mol%]	DIPEA [15 mol%]	1:1.2	100	40	33
3	**E** [10 mol%]	DIPEA [15 mol%]	1:1.2	100	40	97
4	**F** [10 mol%]	DIPEA [15 mol%]	1:1.2	100	40	51
**NHC·HCl**						

^a^Determined by GC–MS using dodecane as an internal standard.

The tabulated comparison shows that in the case of a catalytic system proposed by us (**A**), it is possible to use smaller amounts of the organocatalyst and the base when compared to those needed in the systems based on non-bulky salts (**D**-**F**). Our system also ensures the highest product formation efficiency in the shortest time (24 h vs 40 h) and at the lowest reaction temperature (60 °C vs. 100 °C). These aspects demonstrate the superiority of the proposed method over the known procedures, both financial and environmental. Additionally, the systems described in literature have been tested only for methanol, ethanol and butanol, whereas the system based on precursor **A** has been examined for a wide range of alcohols and aldehydes. Therefore, the proposed system can be applied to a greater number of substrates than the other systems described in literature so far.

A significant advantage of the described method is also its pro-ecological nature. A comparison of its environmental impact with those of the other known methods was conducted using The Green Degree Method (GDM)^39^. Within this study, the catalyst, the base and the solvent have been examined taking into account such aspects as global warming potential (GWP), ozone-depleting potential (ODP), photochemical ozone creation potential (PCOP), acidification potential (AP), eutrophication potential (EP), ecotoxicity potential to water (EPW), ecotoxicity potential to air (EPA), human carcinogenic toxicity potential to water (HCPW), human non-carcinogenic toxicity potential to water (HNCPW). In the described method, acetone is used as the solvent, for which the calculated Green Degree (GD) is -2.3072 (-[GWP], -[ODP] 0.094 [PCOP], -[AP], -[EP], 0.00962 [EPW], 0.0132 [EPA], -[HCPW], 0.006538 [HNCPW]). For comparison, toluene, used as a solvent in the other synthesis methods of esters, described in literature, has a GD of -5.4919 (-[GWP], -[ODP] 0.637 [PCOP], -[AP], -[EP], 1.6269 [EPW], 0.0025 [EPA], -[HCPW], -[HNCPW]). This value is more than twice as high as for acetone. Safety data sheets and literature sources concerning the employed supersteric precursor salt (A) and the base (KHMDS) do not report them as hazardous or harmful to the environment. They do not undergo bioaccumulation and are not considered toxic. Therefore, their environmental impact is negligible and can be omitted when determining the overall environmental impact. Considering all aspects, it can be concluded that the proposed method does not have a significant negative impact on the environment and is the most environmentally friendly among all the presented synthesis routes of esters from alcohols and α,β-unsaturated aldehydes.

Because of full elimination of the need of using metals, oxidants and any other additives, besides the application of a stoichiometric ratio of reagents, low catalyst loading, eco-friendly solvent and full chemoselectivity, the proposed method can be used for the synthesis of products of the cosmetic, food and pharmaceutical industries. In these areas, there is a need to move away from traditional catalytic methods due to the particularly stringent requirements regarding the purity and quality of products. ^5^ The proposed strategy is, for example, an ideal solution to improve the production of esters from polysaccharides, which find applications as non-toxic materials for food packaging or prebiotics. ^40^ Moreover, we have demonstrated that the proposed approach can be easily extended to various types of polyhedral oligomeric silsesquioxanes (SQ), which offers a great chance of using the designed materials as non-toxic drug nanocarriers. Recent development of efficient drug delivery platforms to specific target sites in a controlled manner have attracted wide interest because it is critical for implementation of current advances in diverse areas of medicine. From this point of view, SQs have many benefits. ^1^ Because of their nanoscale size and high charge density, SQ units can be easily transferrable through vascular pores, which considerably improves the uptake by tissues. Unlike traditional organic compounds, SQ derivatives are colorless, nonvolatile, odorless, non-toxic and can be easily functionalized through introduction of reactive organic groups to the vertex positions via various chemical methods. After suitable modifications, the SQ units gain water-solubility which allows the SQ-based drug delivery systems to be taken orally. Additionally, they can be successfully used as the functional group carriers in photodynamic therapy and bioimaging. ^2^ Their great advantage is also high thermal stability, simple synthesis and well-defined 3D structure that eliminates the that arise from the polydispersity of linear polymers. Therefore, the SQs derivatives described in the manuscript seem particularly attractive for applications in the medical and pharmaceutical fields, because they contain the reactive functional groups susceptible to further modification (including deposition of therapeutic substances on them).

## Conclusion

To sum up, we have proved a very high catalytic activity of bulky NHC carbene in the efficient esterification of α,β-unsaturated aldehydes with alcohols. In comparison to previous proposals, the presented protocol allows highly efficient synthesis of a series of esters under mild conditions and low organocatalyst loadings. What is important, the other less bulky NHCs tested in this system proved to be practically inactive. The developed protocol shows a high atom economy and leads to compounds having significant application potential. Their synthetic utility was confirmed by performing several transformations, which allowed selective and efficient syntheses of alkenyl functional organosilicon compounds. It is worth emphasizing that the proposed methodology can also be applied to substrates with multiple functional groups, resulting in air-stable new products that are open to further modifications, i.e. through hydrosilylation hydroboration or hydrothiolation processes. It opens up a possibility of simple syntheses of a number of classes of new compounds with potential for practical application. Consequently, future research should focus on synthesizing esters with multiple bonds and exploring their subsequent uses in chemical synthesis. In this article, on the basis of the experiments performed and literature reports, we proposed a plausible reaction mechanism.

## Methods

### General methods and reagents

All reagents, except NHC carbenes precursors and silsesquioxanes, were commercially available and used as received. NHC salts^19,41,42^, SQ-2OH^43^, DDSQ-4OH^44^ and SQ-SiOMe_2_H^45^ were prepared according to literature procedures. All syntheses and catalytic tests were carried out under argon atmosphere using standard Schlenk-line and vacuum techniques. THF was dried over sodium benzophenone ketyl and freshly distilled before use. The other solvents were dried over CaH_2_ prior to use and stored over 4 Å molecular sieves under argon. Dichloromethane was additionally passed through an alumina column and degassed by repeated freeze–pump–thaw cycles.

### Instruments and measurements

*Nuclear magnetic resonance (NMR) spectroscopy:* ^1^H NMR (402.6 MHz), ^13^C NMR (101.2 MHz) and ^29^Si NMR (79 MHz) spectra were recorded at 25 °C on a Varian 400 and 300 MHz spectrometers in CDCl_3_ solution. Chemical shifts are reported in ppm with reference to the residual solvent peaks for ^1^H and ^13^C NMR and to TMS for ^29^Si NMR.

*Gas chromatography (GC):* GC analyses were carried out on an Agilent 7890B instrument equipped with a DB-530 capillary column (30 m length, 0.53 mm internal diameter) and TCD.

*Gas chromatography—Mass spectrometry (GC–MS):* GC–MS analyses were performed on a Varian Saturn 2100 T instrument equipped with a DB-1 capillary column (30 m length, 0.25 mm internal diameter) and an ion trap detector.

*Electrospray Ionization Mass Spectrometry (ESI–MS):* Mass spectra were obtained using Synapt Gs-S HDMS (Waters) mass spectrometer with electrospray ion source and quadrupole-time-of-flight analyzer with resolving power FWMH 38,000. Acetonitrile was used as the sample solvent. The Capillary Voltage was set to 4.5 kV, the sampling was set 40 and the source temperature was equal to 120 °C. The most abundant ions in ESI–MS spectra were sodiated and potassiated ions of desired products.

*Thin-layer chromatography (TLC):* TLC was conducted on plates coated with 250 μm thick silica gel and column chromatography was performed on silica gel 60 (70–230 mesh) using a mixture of *n*-hexane or *n*-heptane/DCM.

### Procedure for generating free NHC carbene

A flame-dried glass reactor equipped with a stirring bar and connected to a gas and vacuum line was charged with NHC carbene precursor **A** (11.0 mg, 1.12 × 10^–5^ mol, 1 equiv.), KHMDS (2.68 mg, 1.34 × 10^–5^ mol, 1.1 equiv.) and toluene (1.0 mL). After 30 min of vigorous stirring at RT the reaction mixture was filtered under argon into another flame-dried glass reactor equipped with a magnetic stirring bar and connected to gas and vacuum line. The solvent was evaporated and the isolated carbene was washed with *n*-hexane and dried.

### Procedure for catalytic tests

#### Approach A: Reactions based on isolated NHC carbene

A flame-dried glass reactor equipped with a magnetic stirring bar was charged with alcohol (1.6 × 10^–4^ mol), aldehyde (1.6 × 10^–4^ mol), acetone (0.5 mL) and internal standard (decane or dodecane, 20 μL) under argon. Then, the isolated NHC carbene (9,7 mg, 8 × 10^–6^ mol) was added and the reaction mixture was warmed up in an oil bath to 60 °C for 24 h. The reaction course was monitored by GC. Formation of a desired product was confirmed by GC–MS and ^1^H NMR analysis.

#### Approach B: Reactions based on NHC carbene generated in situ

An oven-dried 5 mL glass reactor equipped with a magnetic stirring bar was charged under argon with the NHC carbene precursor **A** (10 mg, 8 × 10^–6^ mol), KHMDS (1.6 mg, 8 × 10^–6^ mol) and acetone (0.5 mL). The reaction mixture was stirred at RT and after 30 min, alcohol (1.6 × 10^–4^ mol), aldehyde (1.6 × 10^–4^ mol) and internal standard (decane or dodecane, 20 μL) were added. The reaction mixture was warmed up in an oil bath to 60 °C The reaction course was monitored by GC. Formation of a desired product was confirmed by GC–MS and ^1^H NMR analysis.

### General procedure for the synthesis of products P1-P17

A flame-dried glass reactor equipped with a magnetic stirring bar was charged with NHC carbene (29 mg, 2.4 × 10^–5^ mol) in the glovebox. Then alcohol (4.8 × 10^–4^ mol), aldehyde (4.8 × 10^–4^ mol) and acetone (1.0 mL) were added under argon. The reaction mixture was stirred at 60 °C for 24 h. The solvent was evaporated under vacuum and the residue was purified by column chromatography on silica gel using 1:1 v/v mixture of *n*-hexane and DCM as eluent. Evaporation of the solvents afforded analytically pure compounds.

### General procedure for the synthesis of products P18-P20

A flame-dried glass reactor equipped with a magnetic stirring bar was charged with NHC carbene (29 mg, 2.4 × 10^–5^ mol) in the glovebox. Then acetone (2.0 mL), aldehyde (2.4 × 10^–4^ mol), catechol or DDSQ-4OH (2.4 × 10^–4^ mol) or SQ-2OH (1.2 × 10^–4^ mol) were added under argon. The reaction mixture was stirred at 60 °C for 24 h. The solvent was evaporated under vacuum and the residue was purified by column chromatography on silica gel using 1:1 v/v mixture of *n*-hexane and DCM (**P18**) and 3:1 v/v mixture of DCM and methanol (**P19**, **P20**). Next, products **P19** and **P20** precipitated from diethyl ether. Evaporation of the solvents afforded analytically pure compounds (**P18**: yellow liquid, 0.80 mg, 90%; **P19**: white solid, 250 mg, 89%; **P20**: white solid, 180 mg, 87%;).

### General procedure for the synthesis of products P21-P23

A 10 mL high-pressure Schlenk vessel was charged with toluene (1 mL), silane (2.9 × 10^–4^ mol) and product **P11** (50 µL, 2.9 × 10^–4^ mol). Then platinum catalyst (1.5 × 10^–7^ mol) was added and the reaction mixture was heated to the 40 °C. When full conversion of Si–H was detected, the solvent was evaporated under vacuum. The residue was purified by column chromatography on silica gel using DCM as eluent. After evaporation of the solvents the products were characterized by spectroscopic methods (**P21**: yellow liquid, 0.92 g, 90%; **P22**: yellow liquid, 90 mg, 97%; **P23**: yellow liquid, 0.89 mg, 89%;).

### One-pot esterification / hydrosilylation process

A 10 mL high-pressure Schlenk vessel equipped with a magnetic stirring bar and connected to the gas and vacuum line was charged with the NHC carbene precursor **A** (10 mg, 8 × 10^–6^ mol), KHMDS (1.6 mg, 8 × 10^–6^ mol) and toluene (2 mL). The reaction mixture was stirred at RT and after hour cinnamaldehyde (20 µL, 1.6 × 10^–4^ mol) and 3-butyn-1-ol (12 µL, 1.6 × 10^–4^ mol) were added. The reaction mixture was warmed up to 60 °C and stirred until full conversions of the reagents were detected by GC–MS. Next, organosilicon compound (1.6 × 10^–4^ mol) and a platinum complex (0.1 mg, 1.6 × 10^–8^ mol) were added. The reaction mixture was stirred at 40 °C for 8-24 h. The solvent was then evaporated under vacuum and the residue was purified by column chromatography on silica gel using DCM as eluent **(P21-P23)** or purified by precipitation from methanol **(P24, P25)**. Evaporation of the solvents afforded analytically pure compounds (**P21**: yellow liquid, 50 mg, 90%; **P22**: yellow liquid, 49 mg, 97%; **P23**: yellow liquid, 45 mg, 89%; **P24**: white solid, 145 mg, 89%; **P25**: white solid, 159 mg, 91%).

### Synthesis of product P1 on a preparative scale

A 10 mL high-pressure Schlenk vessel equipped with a magnetic stirring bar and connected to a gas and vacuum line was charged with the NHC carbene precursor (251 mg, 2 × 10^–4^ mol), KHMDS (40 mg, 2 × 10^–4^ mol) and acetone (5 mL). The reaction mixture was stirred at RT and after 1 h benzyl alcohol (0.83 mL, 4 × 10^–3^ mol) and cinnamaldehyde (1 mL, 4 × 10^–3^ mol) were added. The reaction mixture was stirred at 60 °C for 24 h. Then, the solvent was evaporated under vacuum and the residue was purified using column chromatography (silica gel 60/*n*-hexane : DCM = 1 : 1, DCM). Evaporation of the solvent gave the analytically pure product.

### Supplementary Information

Supplementary Information.

## Data Availability

All data generated or analyzed during this study are included in this published article and its supplementary information file.

## References

[1] Matsumoto, K., Yanagi & R., Oe, Y. *Carboxylic Acid-Key Role in Life Sciences (ed. Badea, G. I., & Radu, B. L.)* 7–34 (IntechOpen, 2018).

[2] Hong, J., Zeng, X. A., Brennan, C. S., Brennan, M. & Han, Z. Recent advances in techniques for starch esters and the applications: A review. *Foods* **5**, 50 (2016). 28231145 10.3390/foods5030050 PMC5302408

[3] SÁ, A. G. A., de Meneses, A. C., de Araújo, P. H. H., & de Oliveira, D. A review on enzymatic synthesis of aromatic esters used as flavor ingredients for food, cosmetics and pharmaceuticals industries. *Trends Food Sci. Technol.,* **69**, 95–105 (2017).

[4] Otera, J., & Nishikido, J. *Esterification: methods, reactions, and applications* 293–320 (John Wiley & Sons*,* 2019).

[5] Ortega-Requena, S. *et al.**Materials* **17**, 268 (2024). 38204120 10.3390/ma17010268 PMC10779758

[6] Capmul 8210 (MCM) Pharmaceutical Grade Bulletin, Capital City Products, Columbus, Ohio.

[7] Neises, B. & Steglich, W. Simple method of the esterification of carboxylic acids. *Angew. Chem. Int. Ed. Engl*. **17**, 522– 524 (1978).

[8] Wiskur, S. L. & Fu, G. C. Catalytic asymmetric synthesis of esters from ketenes. *J. Am. Chem. Soc.* **127**, 6176–6177 (2005). 15853315 10.1021/ja0506152

[9] Majji, G., Rout, S. K., Rajamanickam, S., Guin, S. B. & Patel, K. Synthesis of esters via sp^3^ C-H functionalization. *Org. Biomol. Chem.* **14**, 8178–8211 (2016). 27488288 10.1039/C6OB01250G

[10] Runikhina, S. A., Usanov, D. L., Chizhov, A. O. & Chusov, D. Atom- and step-economical ruthenium-catalyzed synthesis of esters from aldehydes or ketones and carboxylic acids. *Org. Lett.* **20**, 7856–7859 (2018). 30525672 10.1021/acs.orglett.8b03375

[11] Cheng, J. *et al.* Chemoselective dehydrogenative esterification of aldehydes and alcohols with a dimeric rhodium(II) catalyst. *Chem. Sci.* **7**, 4428–4434 (2016). 30155090 10.1039/C6SC00145A PMC6090528

[12] Whittaker, A. M. & Dong, V. M. Nickel-catalyzed dehydrogenative cross-coupling: Direct transformation of aldehydes into esters and amides. *Angew. Chem. Int. Ed.* **54**, 1312–1315 (2015). 10.1002/anie.201410322 PMC4551080 25424967

[13] Chan, A. & Scheidt, K. A. Conversion of α, β-unsaturated aldehydes into saturated esters: an umpolung reaction catalyzed by nucleophilic carbenes. *Org. Lett.* **7**, 905–908 (2005). 15727471 10.1021/ol050100f

[14] Kusuma, S. B. W. *et al.* Direct synthesis of full-biobased cellulose esters from essential oil component α, β-unsaturated aldehydes. *ACS Sustain. Chem. Eng.* **9**, 8450–8457 (2021). 10.1021/acssuschemeng.1c01267

[15] Ekoue-Kovi, K. & Wolf, C. One-pot oxidative esterification and amidation of aldehydes. *Chem. Eur. J.* **14**, 6302–6315 (2008). 18523938 10.1002/chem.200800353

[16] Yatham, V. R. *et al.* 1,4-Bis-Dipp/Mes-1,2,4-trazolylidenes: Carbene catalysts that efficiently overcome steric hindrance in the redox esterification of α- and β-substituted α, β-enals. *J. Am. Chem. Soc.* **138**, 2670–2677 (2016). 26797403 10.1021/jacs.5b11796

[17] Bołt, M., Hanek, K. & Żak, P. Metal-free thioesterification of α, β-unsaturated aldehydes with thiols. *Org. Chem. Front.* **9**, 4846–4853 (2022). 10.1039/D2QO00678B

[18] Bołt, M., Mermela, A., Hanek, K. & Żak, P. Metal-free synthesis of unsymmetric bis(thioesters). *Chem. Commun.* **59**, 956–959 (2023). 10.1039/D2CC05160E 36598061

[19] Żak, P., Bołt, M., Dudziec, B. & Kubicki, M. Synthesis of (*E*)-1,4-disilsesquioxylsubstituted but-1-en-3-ynes *via* platinum-catalyzed dimerization of ethynylsiloxysilsesquioxanes. *Dalton Trans.* **48**, 2657–2663 (2019). 30702739 10.1039/C8DT05142A

[20] Prat, D. *et al.* CHEM21 selection guide of classical- and less classical-solvents. *Green Chem.* **18**, 288–296 (2016). 10.1039/C5GC01008J

[21] Dong, F., Lu, L. & Ha, C. S. Silsesquioxane-containing hybrid nanomaterials: Fascinating platforms for advanced applications. *Macromol. Chem. Phys.* **220**, 1800324 (2019). 10.1002/macp.201800324

[22] McCusker, C., Carroll, J. B., Rotello*,* V. M. Cationic polyhedral oligomeric silsesquioxane (POSS) units as carriers for drug delivery processes. *Chem. Commun.* 996–998 (2005). 10.1039/b416266h 15719094

[23] Kaneshiro, T. L. & Lu, Z. R. Targeted intracellular codelivery of chemotherapeutics and nucleic acid with a well-defined dendrimer-based nanoglobular carrier. *Biomaterials* **30**, 5660–5666 (2009). 19595449 10.1016/j.biomaterials.2009.06.026

[24] Rocca, J. D., Huxford, R. C., Comstock-Duggan, E. & Lin, W. Polysilsesquioxane nanoparticels for targeted platin-based cancer chemotherapy by triggered releas. *Angew. Chem. Int. Ed.* **50**, 10330–10334 (2011). 10.1002/anie.201104510 PMC4040275 21915976

[25] Rocca, J. D., *et.al*. Polysilsesquioxane nanoparticels for triggered release of cisplatin and effective cancer chemoradiotherapy. *Nanomedicine: NBM* **11** (1), 31–38 (2015). 10.1016/j.nano.2014.07.004 PMC4280316 25038495

[26] Pu, C. Y., Zhang, L., Zheng, H., He, B., & Gu, Z. Synthesis and drug release of star-shaped poly(benzyl L-aspartate)-*block*-poly(ethylene glycol) copolymers with POSS cores. *Macromol. Biosci*., **14**, 289–297 (2014). 10.1002/mabi.201300270 23943596

[27] Yang, Q., Li, L., Li, W., Zhou, Z. & Huang, Y. Dual stimuli-responsive hybrid polymeric nanoparticles self-assembled from POSS-based starlike copolymer-drug conjugates for efficient intracellular delivery of hydrophobic drugs. *ACS Appl. Mater. Interf.* **8**(21), 13251–13261 (2016). 10.1021/acsami.6b02403 27167898

[28] John, Ł, Malik, M., Janeta, M. & Szafert, S. First step towards a model system of the drug delivery network based on amide-POSS nanocarriers. *RSC Adv.* **7**, 8394–8401 (2017). 10.1039/C6RA26330E

[29] Rózga-Wijas, K. & Sierant, M. Daunorubicin-silsesquioxane conjugates (POSS-DAU) for theranostic drug delivery system: Characterization, biocompatibility and drug release study. *React. Funct. Polym.* **143**, 104332 (2019). 10.1016/j.reactfunctpolym.2019.104332

[30] Piorecka, K., Jurjata, J. & Stanczyk, W. A. Novel polyhedral silsesquioxanes [POSS(OH)_32_] as anthracycline nanocarriers-potential anticancer prodrugs. *Molecules* **26**, 47 (2021). 10.3390/molecules26010047 PMC7794878 33374161

[31] Loman-Cortes, P., Binte-Hug, T. & Vivero-Escoto, J. L. Use of polyhedral oligomeric silsesquioxane (POSS) in drug delivery. *Photodyn. Ther. Bioimag.. Mol.* **26**, 6453 (2021). 10.3390/molecules26216453 PMC8588151 34770861

[32] Abudukelimu, S., *et.al.* Polyhedral oligomeric silsesquioxanes (POSS)-based hybrid nanocomposite for synergistic chemo-photothermal therapy against pancreatic cancer. *J. Chem. Eng.* 136124 (2022).

[33] Żak, P., Bołt, M., Kubicki, M. & Pietraszuk, C. Highly selective hydrosilylation of olefins and acetylenes by platinum(0) complexes bearing bulky N-heterocyclic carbene ligands. *Dalton Trans.* **47**, 1903–1910 (2018). 29340394 10.1039/C7DT04392A

[34] Pareek, M., Reddi, Y. & Sunoj, R. B. Tale of the Breslow intermediate, a central player in N-heterocyclic carbene organocatalysis: Then and now. *Chem. Sci.* **12**, 7973–7992 (2021). 34194690 10.1039/D1SC01910D PMC8208132

[35] Mahatthananchai, J. & Bode, J. W. The effect of the *N*-mesityl group in NHC-catalyzed reactions. *Chem. Sci.* **3**, 192–197 (2012). 23687565 10.1039/C1SC00397F PMC3655724

[36] Maki, B. E., Patterson, E. V., Cramer, C. J. & Scheidt, K. A. Impact of solvent polarity on N-heterocyclic carbene-catalyzed β-protonations of homoenolate equivalents. *Org. Lett.* **11**, 3942–3945 (2009). 19645427 10.1021/ol901545m PMC2733935

[37] Berkessel, A., Yatham, V. R., Elfert, S. & Neudӧrfl, J.-M. Characterization of the key intermediates of carbene-catalyzed umpolung by NMR spectroscopy and X-ray diffraction: Breslow intermediates, homoenilates, and azolium enolates. *Angew. Chem. Int. Ed.* **52**, 11158–11162 (2013). 10.1002/anie.201303107 24038872

[38] Biswas, A., Neudӧrfl, J.-M., Schlӧrer, N. E. & Berkessel, A. Acyl donor intermediates in N-heterocyclic carbene catalysis: Acyl azolium or azolium enolate?. *Angew. Chem. Int. Ed.* **60**, 4507–4511 (2021). 10.1002/anie.202010348 PMC7986403 33140529

[39] Zhang, X., Li, C., Fu, C. & Zhang, S. Environmental impact assessment of chemical process using the green degree method. *Ind. Eng. Chem. Res.* **47**, 1085–1094 (2008). 10.1021/ie0705599

[40] Ragavan, K. V., Hernandez-Hernandez, O., Martinez, M. M. & Gutiérrez, T. J. Organocatalytic esterification of polysaccharides for food applications: A review. *Trends Food Sci. Technol.* **119**, 45–56 (2022). 10.1016/j.tifs.2021.11.028

[41] Meiries, S., Speck, K., Cordes, D. B., Slawin, A. M. Z. & Nolan, S. P. [Pd(IPr*^OMe^)(acac)Cl]: Tuning the N-heterocyclic carbene in catalytic C-N bond formation. *Organometallics* **32**, 330–339 (2013). 10.1021/om3011867

[42] Hans, M., Lorkowski, J., Demonceau, A. & Delaude, L. Efficient synthetic protocols for the prepartaion of common N-heterocylic carbene precursos. *Beilstein J. Org. Chem.* **11**, 2318–2325 (2015). 26734080 10.3762/bjoc.11.252 PMC4685873

[43] Walczak, M., Franczyk, A., & Marciniec, B. Synthesis of Monofuntionalized Silsesquioxanes (RSiMe_2_O)(*i*Bu)_7_Si_8_O_12_ via Alkene Hydrosilylation. *Chem. Asian J.* **13**, 181–186 (2018) 10.1002/asia.201701569 29194982

[44] Gągor, A., Ślepokura, K. & John, Ł. Hydroxyalkyl-substituted double-decker silsesquioxanes: effective separation of *cis* and *trans* isomers. *Inorg. Chem. Front.* **16**, 3999–4008 (2022).

[45] Waehner, J., Marciniec, B. & Pawluć, P. Functionalization of Vinylspherosilicates by ruthenium-catalyzed silylative coupling reactions. *Eur. J. Inorg. Chem.* **18**, 2975–2980 (2007). 10.1002/ejic.200700142

