# Supplementary material for: Organocatalytic synthetic route to esters and their application in hydrosilylation process
Source: Sci Rep. 2024 Aug 17;14:19108. doi: 10.1038/s41598-024-70036-y (PMC11330490; doi:10.1038/s41598-024-70036-y)
Supplement: Supplementary file 1 — Supplementary Information. [file 41598_2024_70036_MOESM1_ESM.pdf]

Supporting Information

for

Organocatalytic synthetic route to esters and their application in hydrosilylation process

Aleksandra Mermela,[a] Małgorzata Bołt,[a] Aleksandra Mrzygłód[a,b] and Patrycja Żak[a,*]

[a] *Department of Organometallic Chemistry, Faculty of Chemistry, Adam Mickiewicz University in Poznan, Uniwersytetu Poznańskiego 8, 61-614 Poznań, Poland. E-mail: pkw@amu.edu.pl*

[b] *Center for Advanced Technologies, Uniwersytetu Poznańskiego 10, 61-614 Poznan, Poland*

CONTENTS:

1. **Deuterium-labelling experiment** **S-2**
2. **Statistical analysis** **S-3**
3. **Analytical data of isolated products P1-P25** **S-4**
   3.1. Esterification of alcohols with α,β-unsaturated aldehydes **S-4**
   3.2. Esterification of polyols with enals **S-9**
   3.3. Hydrosilylation of product ***P11*** **S-11**
4. **NMR spectra of isolated products P1-P25** **S-13**
5. **References** **S-41**

1. Deuterium-labelling experiment

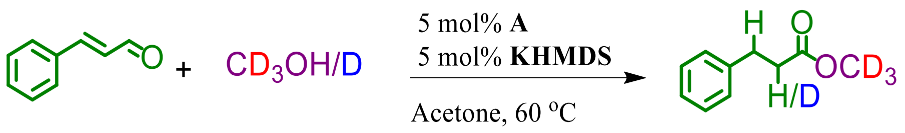

A 10 mL high-pressure Schlenk vessel connected to the gas and vacuum line was charged under argon with the **A** (20 mg, 1.6 × 10^{-5} mol), KHMDS (3.2 mg, 1.6 × 10^{-5} mol) and acetone (0.5 mL). The reaction mixture was stirred at RT and after 30 minutes deuterated methanol (6 µL, 1.6 × 10^{-4} mol) and cinnamaldehyde (20 µL, 1.6 × 10^{-4} mol) were added. The reaction was stirred at 60 °C in a closed vessel for 24 h. Then the solvent was evaporated under vacuum and the resulting product was isolated and purified by chromatography (silica gel 60/*n*-hexane : DCM = 1 : 1). Evaporation of the solvent gave the product.

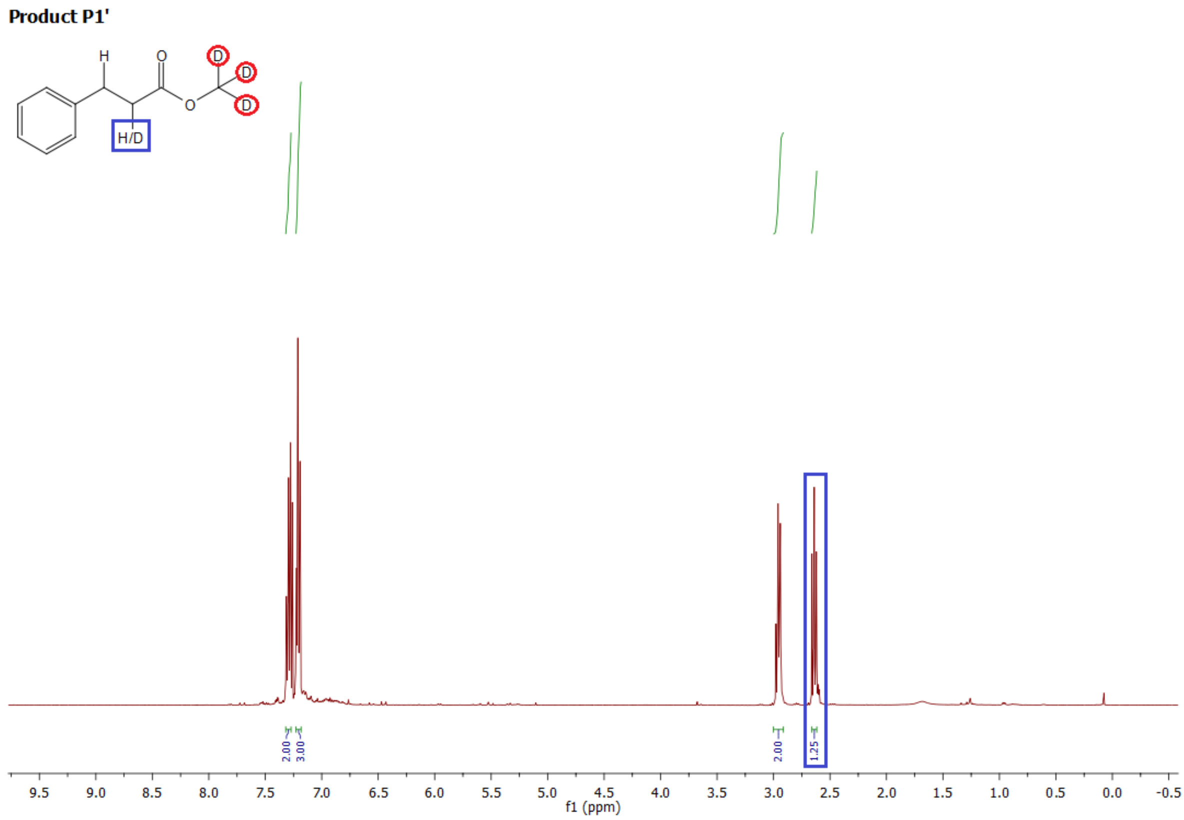

Figure S1. ^{1}H NMR (400 MHz, $CDCl_3$) of product **P1'**. **Signal from CD_3 was not observed.**

2. Statistical analysis

The reaction of cinnamaldehyde (**1a**) with benzyl alcohol (**2a**) was repeated five times under optimized conditions.

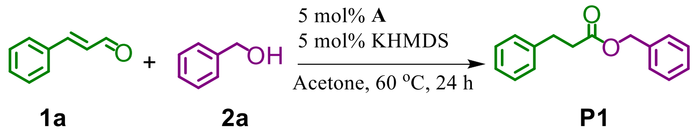

Entry	Yield of P1 [%][a]
1	99
2	98
3	95
4	97
5	98

Reaction conditions: [**1a**]:[**2a**] = 1:1, [**A**]:[**KHMDS**] = 1:1, argon; [a] Determined by GC-MS using dodecane as an internal standard.

For the obtained yield results, the standard deviation was calculated (SD=2) and a Gaussian curve illustrating the reproducibility of yields and reaction conditions was created.

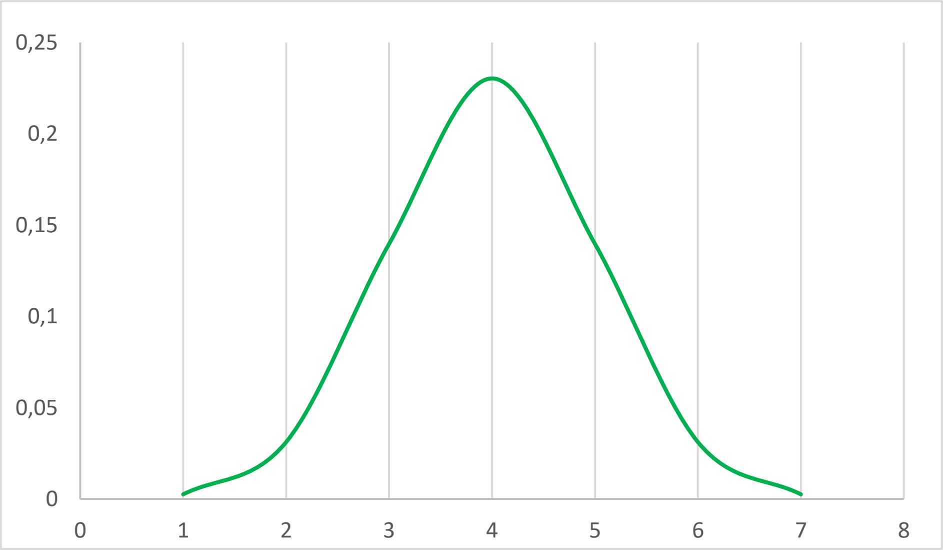

Fig. 1. Normalized Gaussian curve presented reproducibility of yields and reaction conditions

3. Analytical data of isolated products P1-P25

3.1. Esterification of alcohols with α,β-unsaturated aldehydes

Product P1

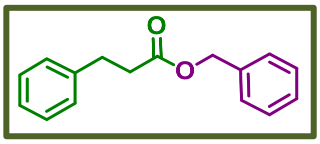

^{1}H NMR (400 MHz, $CDCl_3$, 296K): δ (ppm) 2.69 (t, 2H, J_{HH} = 7.8 Hz, C*H*$_2$), 2.98 (t, 2H, J_{HH} = 7.7 Hz, C*H*$_2$), 5.12 (s, 2H, OC*H*$_2$), 7.17 – 7.23 (m, 3H, C_6*H*$_5$), 7.27 – 7.40 (m, 7H, C_6*H*$_5$); ^{13}C NMR (100 MHz, $CDCl_3$, 296K): δ (ppm) 30.91 (*C*H$_2$), 35.87 (*C*H$_2$), 66.26 (O*C*H$_2$), 126.23, 128.18, 128.27, 128.47, 128.51, 135.86, 140.36, 172.70 (*C*O); MS m/z (rel, intensity): 49.9 (12), 50.9 (11), 64.8 (27), 76.8 (28), 78.8 (29), 90.8 (82), 91.8 (10), 102.7 (10), 104.8 (15), 106.7 (100, $C_6H_5CH_2O$), 179.8 (30), 240 (1, M^+). These data are in accordance with the literature.[1]

Product P2

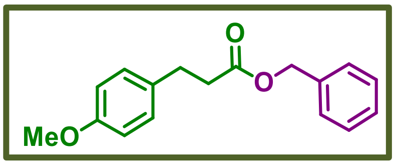

^{1}H NMR (400 MHz, $CDCl_3$, 296K): δ (ppm) 2.62 (t, 2H, J_{HH} = 7.7 Hz, C*H*$_2$), 2.92 (t, 2H, J_{HH} = 7.7 Hz, C*H*$_2$), 3.79 (s, 3H, OC*H*$_3$), 5.11 (s, 2H, OC*H*$_2$), 6.82 (d, 2H, J_{HH} = 8.7 Hz, C_6*H*$_4$-OMe), 7.11 (d, 2H, J_{HH} = 8.7 Hz, C_6*H*$_4$-OMe), 7.28 – 7.31 (m, 5H, C_6*H*$_5$); ^{13}C NMR (100 MHz, $CDCl_3$, 296K): δ (ppm) 30.07 (*C*H$_2$), 36.18 (*C*H$_2$), 55.21 (O*C*H$_3$), 66.20 (O*C*H$_2$), 113.85, 128.16, 128.50, 129.22, 132.41, 135.89, 158.01, 172.77 (*C*O); MS m/z (rel, intensity): MS: 91.0 (20), 121.0 (13), 137.0 (94), 138.0 (9), 178.9 (100, M^+- $C_6H_5CH_2$), 179.9 (11), 192.9 (10), 269.7 (9, M^+). These data are in accordance with the literature.[2]

Product P3

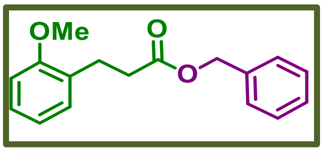

^{1}H NMR (400 MHz, $CDCl_3$, 296K): δ (ppm) 2.68 (t, 2H, J_{HH} = 7.8 Hz, C*H*$_2$), 2.97 (t, 2H, J_{HH} = 7.8 Hz, C*H*$_2$), 3.81 (s, 3H, OC*H*$_3$), 5.12 (s, 2H, OC*H*$_2$), 6.82 – 6.89 (m, 2H, C_6*H*$_4$-OMe), 7.12 – 7.15 (m, 1H, C_6*H*$_4$-OMe), 7.18 – 7.23 (m, 1H, C_6*H*$_4$-OMe), 7.29 – 7.41 (m, 5H, C_6*H*$_5$); ^{13}C NMR (100 MHz, $CDCl_3$, 296K): δ (ppm) 26.10 (*C*H$_2$), 34.16 (*C*H$_2$), 55.12 (O*C*H$_3$), 66.09 (O*C*H$_2$), 110.13, 120.37, 127.56, 128.09, 128.15, 128.48, 136.04, 157.43, 173.19 (*C*O); MS m/z (rel, intensity): 91.2 (17), 105.2 (13), 133.5 (13), 135.2 (38), 136.2 (13), 160.1 (23), 161.0 (52), 162.0 (15), 193.2 (19), 246.0 (14), 247.8 (15), 249.2 (18), 251.2 (14), 252.8 (100, M^+-CH_3), 254.0 (19), 269.1 (22), 269.7 (31, M^+). These data are in accordance with the literature.[3]

Product P4

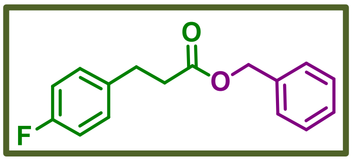

^{1}H NMR (400 MHz, $CDCl_3$, 296K): δ (ppm) 2.66 (t, 2H, J_{HH} = 7.6 Hz, CH_2), 2.94 (t, 2H, J_{HH} = 7.6 Hz, CH_2), 5.11 (s, 2H, OCH_2), 6.92 – 6.98 (m, 2H, C_6H_4-F), 7.11 – 7.16 (m, 2H, C_6H_4-F), 7.28 – 7.39 (m, 5H, C_6H_5); ^{13}C NMR (100 MHz, $CDCl_3$, 296K): δ (ppm) 30.09 (CH_2), 35.94 (CH_2), 66.28 (OCH_2), 115.20 (d, *J* = 21.1 Hz), 128.19 (br s), 128.51 (br s), 129.69 (d, *J* = 7.9 Hz), 135.78, 135.94 (d, *J* = 3.2 Hz), 161.43 (d, *J* = 244.0 Hz), 172.47 (*C*O); MS m/z (rel, intensity): 65.0 (14), 77.0 (12), 91.1 (70), 92.1 (10), 103.0 (10), 109.1 (19), 125.0 (100, F-$C_6H_5CH_2CH_2$), 166.0 (11), 166.9 (27), 197.9 (31), 241.0 (28), 258.0 (4, M^+).

Product P5

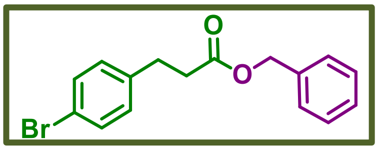

^{1}H NMR (400 MHz, $CDCl_3$, 296K): δ (ppm) 2.66 (t, 2H, J_{HH} = 7.6 Hz, CH_2), 2.92 (t, 2H, J_{HH} = 7.6 Hz, CH_2), 5.10 (s, 2H, OCH_2), 7.05 (d, 2H, J_{HH} = 8.6 Hz, C_6H_4-Br), 7.27 – 7.31 (m, 2H, C_6H_4-Br), 7.32 – 7.42 (m, 5H, C_6H_5); ^{13}C NMR (100 MHz, $CDCl_3$, 296K): δ (ppm) 30.29 (CH_2), 35.60 (CH_2), 66.34 (OCH_2), 120.05, 128.22, 128.25, 128.53, 130.07, 131.51, 135.73, 139.27, 172.35 (*C*O); MS m/z (rel, intensity): 89.5 (16), 91.3 (54), 92.2 (25), 103.2 (16), 104.0 (16), 258.2 (81), 259.1 (23), 260.0 (100, M^+- C_4H_4), 261.0 (17), 301.0 (35), 302.8 (34), 318.9 (1, M^+). These data are in accordance with the literature.[4]

Product P6

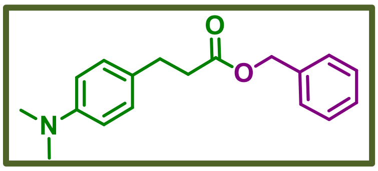

^{1}H NMR (400 MHz, $CDCl_3$, 296K): δ (ppm) 2.64 (t, 2H, J_{HH} = 7.8 Hz, CH_2), 2.88 (t, 2H, J_{HH} = 7.8 Hz, CH_2), 2.92 (s, 6H, CH_3), 5.11 (s, 2H, OCH_2), 6.65 – 6.77 (m, 2H, C_6H_4-NMe_2), 7.08 (d, 2H, J_{HH} = 8.6 Hz, C_6H_4-NMe_2), 7.30 – 7.39 (m, 5H, C_6H_5); ^{13}C NMR (100 MHz, $CDCl_3$, 296K): δ (ppm) 30.02 (CH_2), 36.26 (CH_2), 41.07 (CH_3), 66.17 (OCH_2), 113.28, 128.13, 128.17, 128.34, 128.49, 128.97, 129.16, 135.95, 172.94 (*C*O); MS m/z (rel, intensity): 64.8 (11), 77.0 (11), 91.0 (25), 118.0 (9), 134.0 (92), 135.0 (11), 146.0 (13), 146.9 (12), 148.0 (9), 149.9 (27), 191.9 (65), 283.0 (100, M^+).

Product P7

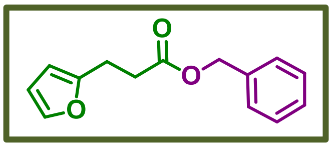

^{1}H NMR (400 MHz, $CDCl_3$, 296K): δ (ppm) 2.72 (t, 2H, J_{HH} = 7.6 Hz, CH_2), 3.00 (t, 2H, J_{HH} = 7.6 Hz, CH_2), 5.14 (s, 2H, OCH_2), 6.01 (dd, 1H, J_{HH} = 3.2, 0.9 Hz, C*H* from furan), 6.27 (dd, 1H, J_{HH} = 3.2, 1.9 Hz, C*H* from furan), 7.28 – 7.31 (m, 1H, C*H* from furan), 7.32 – 7.40 (m, 5H, C_6H_5); ^{13}C NMR (100 MHz, $CDCl_3$, 296K): δ (ppm) 23.40 (CH_2), 32.68 (CH_2), 66.35 (OCH_2), 105.31, 110.14, 128.18, 128.20, 128.51, 135.80, 141.18, 153.59, 172.30 (*C*O); MS m/z (rel, intensity): 65.0 (13), 80.9 (11), 91.2 (33), 92.1 (10), 97.0 (16), 138.0 (15), 139.0 (100, M^+- $C_6H_5CH_2$), 139.9 (10), 162.0 (10), 171.1 (16), 230.0 (25, M^+). These data are in accordance with the literature.[4]

Product P8

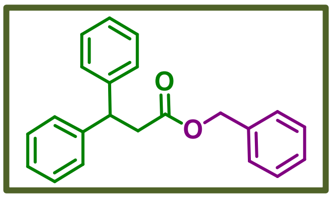

^{1}H NMR (400 MHz, $CDCl_3$, 296K): δ (ppm) 3.12 (d, 2H, J_{HH} = 8.1 Hz, CH_2), 4.57 (t, 1H, J_{HH} = 8.3 Hz, C*H*), 5.02 (s, 2H, OCH_2), 7.12 – 7.25 (m, 10H, C_6H_5), 7.27 – 7.28 (m, 2H, C_6H_5), 7.29 – 7.31 (m, 3H, C_6H_5); ^{13}C NMR (100 MHz, $CDCl_3$, 296K): δ (ppm) 40.78 (CH_2), 47.07 (*C*H), 66.29 (OCH_2), 126.54, 127.64, 127.93, 128.04, 128.07, 128.18, 128.31, 128.44, 128.55, 129.11, 135.71, 143.30, 171.93 (*C*O); MS m/z (rel, intensity): 105.0 (13), 165.0 (16), 167.1 (16), 182.2 (13), 183.2 (59), 184.1 (17), 224.3 (17), 225.2 (100, M^+- $C_6H_5CH_2$), 226.0 (23), 316.0 (2, M^+). These data are in accordance with the literature.[2]

Product P9

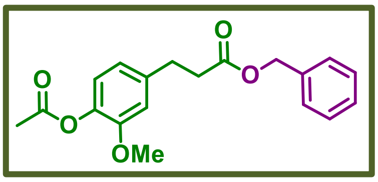

^{1}H NMR (400 MHz, $CDCl_3$, 296K): δ (ppm) 2.30 (s, 3H, CH_3), 2.69 (t, 2H, J_{HH} = 7.8 Hz, CH_2), 2.96 (t, 2H, J_{HH} = 7.8 Hz, CH_2), 3.78 (s, 3H, OCH_3), 5.12 (s, 2H, OCH_2), 6.74 – 6.80 (m, 2H, C_6H_3-), 6.93 (2, 1H, J_{HH} = 8.0 Hz, C_6H_3-), 7.29 – 7.39 (m, 5H, C_6H_5); ^{13}C NMR (100 MHz, $CDCl_3$, 296K): δ (ppm) 20.66 (CH_3), 30.79 (CH_2), 35.79 (CH_2), 55.76 (OCH_3), 66.35 (OCH_2), 112.53, 120.32, 122.62, 128.21, 128.54, 135.81, 138.07, 139.35, 150.84, 169.15 (*C*O), 172.55 (*C*O); MS m/z (rel, intensity): 91.2 (13), 153.2 (25), 194.2 (13), 195.2 (53), 285.9 (100, M^+ - CH_3CO), 286.8 (17), 328.0 (6, M^+).

Product P10

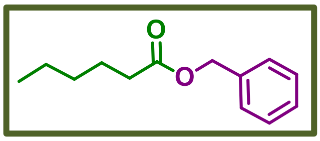

^{1}H NMR (400 MHz, $CDCl_3$, 296K): δ (ppm) 0.88 (t, 3H, J_{HH} = 7.0 Hz, CH_3), 1.07 – 1.35 (m, 4H, CH_2), 1.61 – 1.69 (m, 2H, CH_2), 2.35 (t, 2H, J_{HH} = 7.8 Hz, CH_2), 5.12 (s, 2H, OCH_2), 7.30 – 7.39 (m, 5H, C_6H_5); ^{13}C NMR (100 MHz, $CDCl_3$, 296K): δ (ppm) 13.89 (CH_2), 22.28 (CH_3), 24.62 (CH_2), 31.26 (CH_2), 34.28 (CH_2), 66.04 (OCH_2), 128.14, 128.51, 136.09, 173.71 (*C*O); MS m/z (rel, intensity): 64.8 (14), 91.0 (100, $C_6H_6CH_2$), 91.9 (14), 98.9 (9), 106.8 (10), 107.8 (47), 114.8 (15), 205.7 (3, M^+). These data are in accordance with the literature.[5]

Product P11

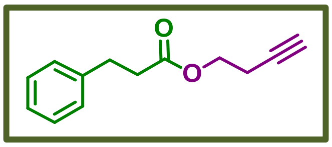

^{1}H NMR (400 MHz, $CDCl_3$, 296K): δ (ppm) 1.99 (t, 1H, J_{HH} = 2.7 Hz, C*H*), 2.50 (dt, 1H, J_{HH} = 6.8, 2.7 Hz, CH_2), 2.66 (t, 2H, J_{HH} = 7.8 Hz, CH_2), 2.97 (t, 2H, J_{HH} = 7.8 Hz, CH_2), 4.19 (t, 2H, J_{HH} = 6.8 Hz, OCH_2), 7.18 – 7.23 (m, 3H, C_6H_5), 7.27 – 7.32 (m, 2H, C_6H_5); ^{13}C NMR (100 MHz, $CDCl_3$, 296K): δ (ppm) 18.92 (CH_2), 30.85 (CH_2), 35.70 (CH_2), 62.06 (OCH_2), 69.86 (C*H*), 79.99 (*C*≡), 126.26, 128.26, 128.47, 140.33, 172.59 (*C*O); MS m/z (rel, intensity): 50.0 (16), 51.1 (18), 65.0 (15), 77.0 (17), 79.0 (17), 91.0 (52), 103.2 (25), 104.0 (95), 105.0 (100, $C_6H_5CH_2CH_2$), 106.8 (30), 132.9 (23), 173.9 (21), 202.2 (24, M^+). These data are in accordance with the literature.[6]

Product P12

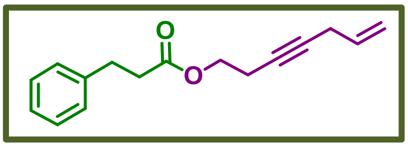

^{1}H NMR (400 MHz, $CDCl_3$, 296K): δ (ppm) 2.46 – 2.56 (m, 2H, CH_2), 2.64 – 2.70 (m, 2H, CH_2), 2.92 – 3.00 (m, 4H, CH_2), 4.17 (t, 2H, J_{HH} = 6.8 Hz, OCH_2), 5.08 – 5.13 (m, 1H, =C*H*), 5.28 – 5.34 (m, 1H, =C*H*), 5.76 – 5.87 (m, 1H, =C*H*), 7.18 – 7.29 (m, 3H, C_6H_5), 7.27 – 7.32 (m, 2H, C_6H_5); ^{13}C NMR (100 MHz, $CDCl_3$, 296K): δ (ppm) 19.23 (CH_2), 22.99 (CH_2), 30.88 (CH_2), 35.77 (CH_2), 62.68 (OCH_2), 78.13 (*C*≡), 78.36(*C*≡), 115.81 (=*C*H), 126.22, 128.24, 128.4, 132.79 (=CH_2), 140.39, 172.63 (*C*O); MS m/z (rel, intensity): 91.0 (19), 91.9 (13), 93.0 (12), 103.0 (13), 104.2 (33), 105.0 (69), 233.2 (10), 241.9 (10), 242.9 (100, M^+).

Product P13

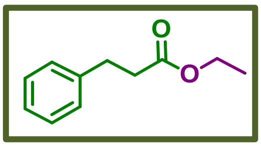

^{1}H NMR (400 MHz, $CDCl_3$, 296K): δ (ppm) 1.24 (t, 3H, J_{HH} = 7.1 Hz, CH_3), 2.60 – 2.65 (m, 2H, CH_2), 2.93 – 2.98 (m, 2H, CH_2), 4.13 (q, 2H, J_{HH} = 7.1 Hz, OCH_2), 7.17 – 7.25 (m, 3H, C_6H_5), 7.26 – 7.31 (m, 2H, C_6H_5);^{13}C NMR (100 MHz, $CDCl_3$, 296K): δ (ppm) 14.17 (CH_3), 30.94 (CH_2), 35.92 (CH_2), 60.38 (OCH_2), 126.18, 128.26, 128.43, 140.53, 172.89 (CO); MS m/z (rel, intensity): 102.2 (5), 103.2 (12), 104.2 (55), 105.2 (28), 132.9 (8), 176.0 (5), 176.9 (9), 177.8 (20), 178.8 (100, M^+).These data are in accordance with the literature.[7]

Product P14

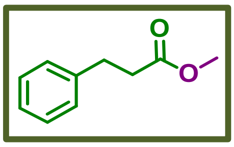

^{1}H NMR (400 MHz, $CDCl_3$, 296K): δ (ppm) 2.64 (t, 2H, J_{HH} = 7.9 Hz, CH_2), 2.93 – 2.98 (m, 2H, OCH_2), 3.68 (s, 3H, CH_3), 7.18 – 7.24 (m, 3H, C_6H_5), 7.27 – 7.32 (m, 2H, C_6H_5); ^{13}C NMR (100 MHz, $CDCl_3$, 296K): δ (ppm) 30.91 (CH_3), 35.68 (CH_2), 51.61 (OCH_2), 126.23, 128.24, 128.47, 140.47, 173.32 (CO); MS m/z (rel, intensity): 65.0 (4), 91.0 (17), 91.9 (5), 103.0 (12), 104.0 (100, $C_6H_5CH_2CH_2$), 105.0 (38), 131.0 (4), 132.9 (7), 162.8 (6), 163.8 (18), 164.7 (27, M^+). These data are in accordance with the literature.[18]

Product P15

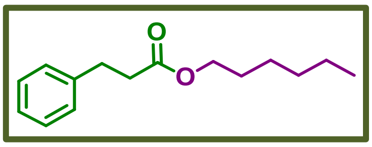

^{1}H NMR (400 MHz, $CDCl_3$, 296K): δ (ppm) 0.89 (t, 3H, J_{HH} = 6.8 Hz, CH_3), 1.25 – 1.33 (m, 6H, CH_2), 1.55 – 1.61 (m, 4H, CH_2), 2.63 (t, 2H, J_{HH} = 7.8 Hz, CH_2), 2.92 – 2.98 (m, 2H, CH_2), 4.06 (t, 2H, J_{HH} = 6.7 Hz, OCH_2), 7.18 – 7.22 (m, 3H, C_6H_5), 7.26 – 7.31 (m, 2H, C_6H_5); ^{13}C NMR (100 MHz, $CDCl_3$, 296K): δ (ppm) 13.99 (CH_2), 22.51 (CH_3), 25.54 (CH_2), 28.54 (CH_2), 30.98 (CH_2), 31.40 (CH_2), 35.92 (CH_2), 64.65 (OCH_2), 126.19, 128.26, 128.44, 140.55, 173.01 (CO); MS m/z (rel, intensity): 91.0 (17), 103.0 (8), 104.0 (100, $C_6H_5CH_2CH_2$), 105.0 (31), 106.8 (12), 106.8 (12), 132.9 (7), 149.8 (28), 150.7 (7), 234.8 (6, M^+). These data are in accordance with the literature.[9]

Product P16

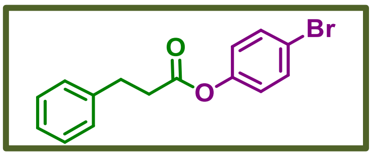

^{1}H NMR (400 MHz, $CDCl_3$, 296K): δ (ppm) 2.86-2.91 (m, 2H, CH_2), 3.07 (t, 2H, J_{HH} = 7.8 Hz, CH_2), 6.90 (d, 2H, J_{HH} = 9.0 Hz, -C_6H_4-Br-4), 7.22 – 7.25 and 7.26-7.28 (m, 3H, C_6H_5-), 7.30-7.35 (m, 2H, C_6H_5-), 7.47 (d, 2H, J_{HH} = 9.0 Hz, -C_6H_4-Br-4); ^{13}C NMR (100 MHz, $CDCl_3$, 296K): δ (ppm) 30.89 (CH_2), 35.91 (CH_2), 118.85, 123.31, 126.50, 128.36, 128.60, 132.41, 139.92, 149.66, 171.02 (CO).

Product P17

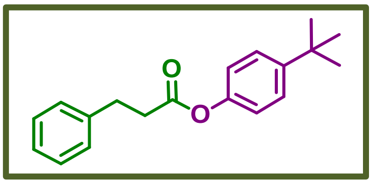

^{1}H NMR (400 MHz, $CDCl_3$, 296K): δ (ppm) 1.33 (s, 9H, CH_3), 2.85-2.93 (m, 2H, CH_2), 3.09 (t, 2H, J_{HH} = 7.7 Hz, CH_2), 6.95 (d, 2H, J_{HH} = 8.9 Hz, -C_6H_4-tBu) 7.23 – 7.25 and 7.26-7.35 (m, 5H, C_6H_5), 7.38 (d, 2H, J_{HH} = 8.9 Hz, -C_6H_4-tBu); ^{13}C NMR (100 MHz, $CDCl_3$, 296K): δ (ppm) 30.98 (CH_2), 31.38 ($C(CH_3)_3$), 34.43 ($C(CH_3)_3$), 35.98 (CH_2), 120.78, 126.24, 126.38, 128.38, 128.54, 140.17, 148.29, 148.55, 171.50 (CO).

3.2. Esterification of polyols with enals

Product P18

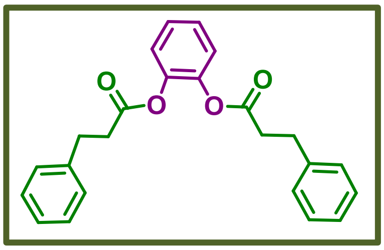

^{1}H NMR (400 MHz, $CDCl_3$, 296K): δ (ppm) 2.78 (t, 4H, J_{HH} = 7.7 Hz, CH_2), 3.03 (t, 4H, J_{HH} = 7.6 Hz, CH_2), 7.10 – 7.15 (m, 2H, C_6H_4), 7.18 – 7.26 (m, 8H, C_6H_4 and C_6H_5), 7.29 – 7.34 (m, 4H, C_6H_5); ^{13}C NMR (100 MHz, $CDCl_3$, 296K): δ (ppm) 30.68 (CH_2), 35.41 (CH_2), 123.63, 126.48 (J = 11.1 Hz), 128.44 (J = 20.1 Hz), 139.91, 142.00, 170.21 (CO); MS (ESI+): m/z 374 $[M+Na]^+$.

Product P19

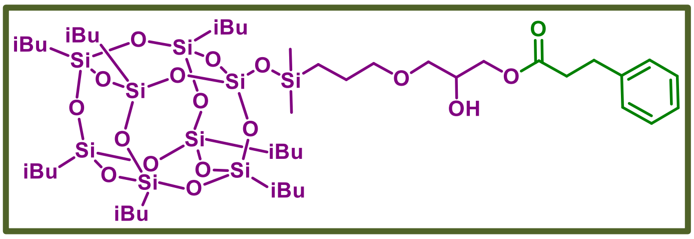

^{1}H NMR (400 MHz, $CDCl_3$, 296K): δ (ppm) 0.12 (s, 6H, Si(CH_3)$_2$), 0.50 - 0.69 (m, 16H, CH_2), 0.85 – 1.10 (m, 42H, CH_3), 1.58 – 1.66 (m, 2H, CH_2), 1.81 – 1.92 (m, 7H, C*H*), 2.41 (br s, 1H, O*H*), 2.69 (t, 2H, J_{HH} = 7.7 Hz, CH_2), 2.97 (t, 2H, J_{HH} = 7.7 Hz, CH_2), 3.35 – 3.47 (m, 4H, CH_2), 3.93 – 3.98 (m, 1H, C*H*), 4.09 – 4.19 (m, 2H, CH_2), 7.18 – 7.23 (m, 3H, C_6H_5), 7.27 – 7.32 (m, 2H, C_6H_5); ^{13}C NMR (100 MHz, $CDCl_3$, 296K): δ (ppm) -0.21 (SiCH_3), 13.39 (SiCH_2), 23.46 (SiCH_2CH_2), 24.23 (CH_2), 24.91 (*C*H), 25.12 (CH_3), 31.58 (CH_2), 36.15 (CH_2), 64.72 (OCH_2), 71.05 (*C*H), 72.74 (OCH_2), 74.33 (OCH_2), 127.67, 128.37, 128.51, 129.25, 129.35, 142.36, 161.47 (*C*O); ^{29}Si NMR (79 MHz, $CDCl_3$, 296K): -11.51 (*Si*CH_3), -60.07, -67.85, -109.64 (core).

Product P20

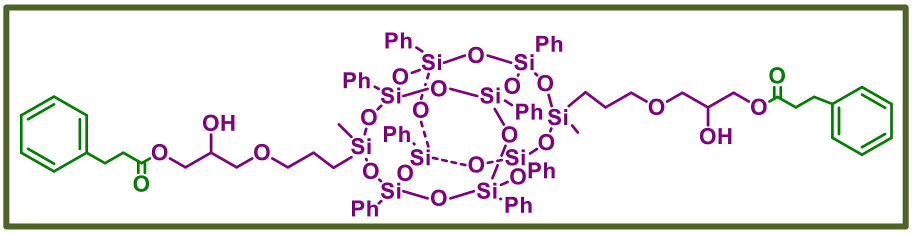

^{1}H NMR (400 MHz, $CDCl_3$, 296K): δ (ppm) 0.32 (s, 6H, Si(CH_3)$_2$), 0.66 - 0.79 (m, 4H, CH_2), 1.26 – 1.31 (m, 2H, CH_2), 1.62 – 1.72 (m, 4H, CH_2), 2.52 – 2.97 (br m, 2H, O*H*), 2.65 (t, 2H, J_{HH} = 7.7 Hz, CH_2), 2.90 – 2.96 (m, 2H, CH_2), 3.11 – 3.35 (m, 8H, CH_2), 3.38 – 3.62 (m, 4H, CH_2), 3.66 – 3.70 (m, 1H, C*H*), 3.76 – 3.83 (m, 1H, C*H*), 3.93 – 4.05 (m, 2H, CH_2), 6.68 – 6.73 (m, 2H, C_6H_5), 6.86 – 6.94 (m, 2H, C_6H_5), 6.99 – 7.24 (m, 20H, C_6H_5), 7.26 – 7.31 (m, 5H, C_6H_5), 7.33 – 7.46 (m, 15H, C_6H_5), 7.51 – 7.55 (m, 6H, C_6H_5); ^{13}C NMR (100 MHz, $CDCl_3$, 296K): δ (ppm) -0.92 (SiCH_3), 1.00 (SiCH_3), 12.68 (SiCH_2), 22.75 (SiCH_2CH_2), 51.51 (CH_2), 55.14 (CH_2), 64.01 (OCH_2), 70.33 (OCH_2), 72.03 (*C*H), 73.62 (OCH_2), 115.58, 123.50, 126.96, 127.66, 127.80, 128.25, 128.47, 128.54, 128.64, 128.88, 129.13, 129.93, 130.40, 131.87, 133.85, 133.99, 141.65, 142.34, 142.61, 160.76 (*C*O); ^{29}Si NMR (79 MHz, $CDCl_3$, 296K): δ (ppm) -17.51 (*Si*CH_3), -78.63, -79.57 (core).

3.3. Hydrosilylation of product P11

Product P21

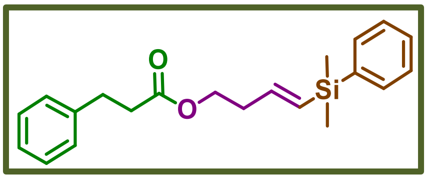

^1H NMR (400 MHz, $CDCl_3$, 296K): δ (ppm) 0.38 (s, 6H, $Si(CH_3)_2$), 2.49 – 2.43 (m, 2H, CH_2), 2.58 – 2.65 (m, 2H, CH_2), 2.91 – 2.95 (m, 2H, CH_2), 4.15 (t, 2H, J_{HH} = 6.8 Hz, OCH_2), 5.87 (dt, 1H, J_{HH} = 18.6, 1.4 Hz, =CH), 6.05 (dt, 1H, J_{HH} = 18.6, 6.2 Hz, =CH), 7.17 – 7.22 (m, 3H, C_6H_5), 7.26 – 7.31 (m, 2H, C_6H_5), 7.32 – 7.37 (m, 3H, C_6H_5), 7.48 – 7.53 (m, 2H, C_6H_5); ^{13}C NMR (100 MHz, $CDCl_3$, 296K): δ (ppm) -2.64 (CH_3), 30.93 (CH_2), 35.81 (CH_2), 35.88 (CH_2), 63.20 (OCH_2), 126.21, 127.72, 128.24, 128.45, 128.93, 131.07, 133.75, 138.67, 140.47, 143.56, 172.83 (CO); ^{29}Si NMR (79 MHz, $CDCl_3$, 296K): δ (ppm) -11.79 ($SiMe_2Ph$); MS m/z (rel, intensity): 91.2 (15), 103.0 (23), 105.0 (76), 105.9 (28), 106.8 (16), 111.2 (15), 128.0 (15), 129.3 (16), 130.1 (15), 131.0 (18), 135.2 (33), 161.0 (21), 188.2 (17), 189.2 (27), 190.3 (15), 191.2 (16), 205.1 (15), 206.2 (19), 207.3 (48), 208.2 (17), 233.3 (42), 234.3 (17), 264.2 (16), 269.0 (100, M^+-C_5H_5), 270.0 (25), 294.9 (40), 338.2 (5, M^+).

Product P22

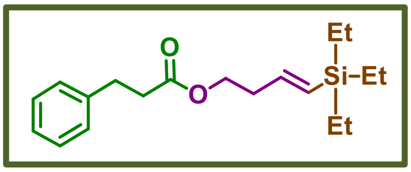

^1H NMR (400 MHz, $CDCl_3$, 296K): δ (ppm) 0.49 – 0.61 (m, 6H, CH_3CH_2), 0.93 (t, 9H, J_{HH} = 7.9 Hz, CH_3CH_2), 2.38 – 2.49 (m, 2H, CH_2), 2.59 – 2.65 (m, 2H, CH_2), 2.92 – 2.98 (m, 2H, CH_2), 4.15 (t, 2H, J_{HH} = 6.8 Hz, OCH_2), 5.67 (dt, 1H, J_{HH} = 18.7, 1.5 Hz, =CH), 5.97 (dt, 1H, J_{HH} = 18.8, 6.3 Hz, =CH), 7.17 – 7.23 (m, 3H, C_6H_5), 7.27 – 7.31 (m, 2H, C_6H_5); ^{13}C NMR (100 MHz, $CDCl_3$, 296K): δ (ppm) 3.36 (CH_3CH_2), 7.31 (CH_3CH_2), 30.94 (CH_2), 35.89 (CH_2), 36.06 (CH_2), 63.42 (OCH_2), 126.20, 128.23, 128.45, 129.46, 140.51, 142.93, 172.83 (CO); ^{29}Si NMR (79 MHz, $CDCl_3$, 296K): δ (ppm) -1.43 ($SiEt_3$); MS m/z (rel, intensity): 81.2 (30), 95.2 (18), 99.7 (41), 101.0 (86), 102.2 (43), 103.2 (34), 105.2 (55), 106.2 (36), 106.8 (32), 107.7 (29), 108.3 (30), 109.3 (32), 110.1 (33), 110.7 (32), 111.5 (34), 112.2 (36), 113.5 (50), 114.1 (53), 115.2 (100, M^+ - $SiEt_3$), 116.2 (32), 289.2 (62), 301.2 (21), 318.2 (11, M^+).

Product P23

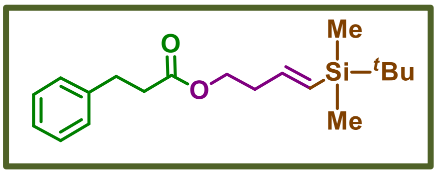

^{1}H NMR (400 MHz, $CDCl_3$, 296K): δ (ppm) 0.01 and 0.0 (s, 6H, Si(CH_3)$_2$), 0.85 (s, 9H, C(CH_3)$_3$), 2.38 – 2.47 (m, 2H, CH_2), 2.58 – 2.65 (m, 2H, CH_2), 2.91 – 2.97 (m, 2H, CH_2), 4.14 (t, 2H, J_{HH} = 6.8 Hz, OCH_2), 5.73 (dt, 1H, J_{HH} = 18.6, 1.4 Hz, =CH), 5.97 (dt, 1H, J_{HH} = 18.6, 6.3 Hz, =CH), 7.18 – 7.23 (m, 3H, C_6H_5), 7.27 – 7.32 (m, 2H, C_6H_5); ^{13}C NMR (100 MHz, $CDCl_3$, 296K): δ (ppm) 6.18 (Si(CH_3)$_2$), 25.76 (C(CH_3)$_3$), 26.36 (C(CH_3)$_3$), 30.95 (CH_2), 35.91 (CH_2), 35.96(CH_2), 63.39 (OCH_2), 126.22, 128.25, 128.46, 130.45, 142.94, 149.62; MS m/z (rel, intensity):73.1 (16), 75.0 (39), 77.1 (7), 91.0 (19), 105.0 (18), 111.0 (7), 207.0 (100,), 208.0 (16), 209.0 (6), 249.0 (5), 261.0 (7), 318.0 (6, M^+).

Product P24

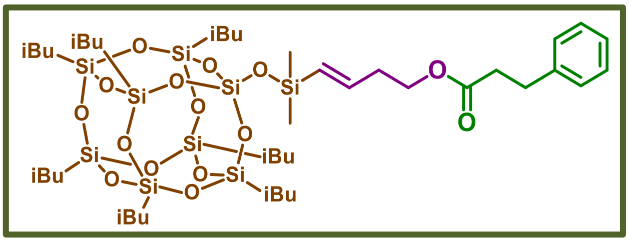

^{1}H NMR (400 MHz, $CDCl_3$, 296K): δ (ppm) 0.07 and 0.16 (s, 6H, Si(CH_3)$_2$), 0.56 – 0.64 (m, 14H, CH_2), 0.91 – 1.01 (m, 42H, CH_3), 1.81 – 1.90 (m, 7H, CH), 2.37 – 2.46 (m, 2H, CH_2), 2.60 – 2.65 (m, 2H, CH_2), 2.91 – 2.97 (m, 2H, CH_2), 4.13 (t, 2H, J_{HH} = 7.0 Hz, OCH_2), 5.72 (dt, 1H, J_{HH} = 18.7, 1.4 Hz, =CH), 6.07 (dt, 1H, J_{HH} = 18.8, 6.2 Hz, =CH), 7.16 – 7.23 (m, 3H, C_6H_5), 7.26 – 7.31 (m, 2H, C_6H_5); ^{13}C NMR (100 MHz, $CDCl_3$, 296K): δ (ppm) 0.07 (CH_3), 1.01 (CH_3), 22.36 (CH_2), 22.40 (CH_2), 23.79 (CH), 23.83 (CH), 25.67 (br s, CH_3), 30.94 (CH_2), 35.50 (CH_2), 35.88 (CH_2), 63.18 (OCH_2), 126.22, 128.25, 128.46, 131.71, 140.47, 142.78, 172.79 (CO); ^{29}Si NMR (79 MHz, $CDCl_3$, 296K): δ (ppm) -1.08 (Si(CH_3)$_2$), -67.07, -67.86, -109.58 (core); MS (ESI+): m/z 1131 $[M+K]^+$.

Product P25

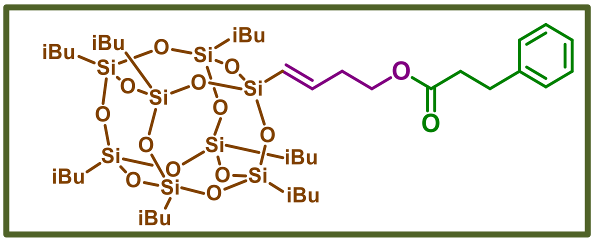

^{1}H NMR (400 MHz, $CDCl_3$, 296K): δ (ppm) 0.49 – 0.73 (m, 14H, CH_2), 0.78 – 1.11 (m, 42H, CH_3), 1.79 – 1.91 (m, 7H, CH), 2.41 – 2.48 (m, 2H, CH_2), 2.62 (t, 2H, J_{HH} = 7.9, CH_2), 2.94 (t, 2H, J_{HH} = 7.9, CH_2), 4.14 (t, 2H, J_{HH} = 6.8 Hz, OCH_2), 5.50 (dt, 1H, J_{HH} = 18.7, 1.5 Hz, =CH), 6.32 (dt, 1H, J_{HH} = 18.7, 6.3 Hz, =CH), 7.17 – 7.23 (m, 3H, C_6H_5), 7.27 – 7.31 (m, 2H, C_6H_5); ^{13}C NMR (100 MHz, $CDCl_3$, 296K): δ (ppm) 22.40 (CH_2), 22.48 (CH_2), 23.83 (CH), 25.67 (CH_3), 25.59 (CH_3), 30.91 (CH_2), 35.25 (CH_2), 35.83 (CH_2), 62.96 (OCH_2), 123.32, 126.22, 128.25, 128.46, 140.43, 146.87, 172.81 (CO); ^{29}Si NMR (79 MHz, $CDCl_3$, 296K): δ (ppm) -67.50, -67.87 (core), -81.31 (=CH*Si*); MS (ESI+): m/z 1042 $[M+Na]^+$.

4. NMR spectra of isolated products P1-P25

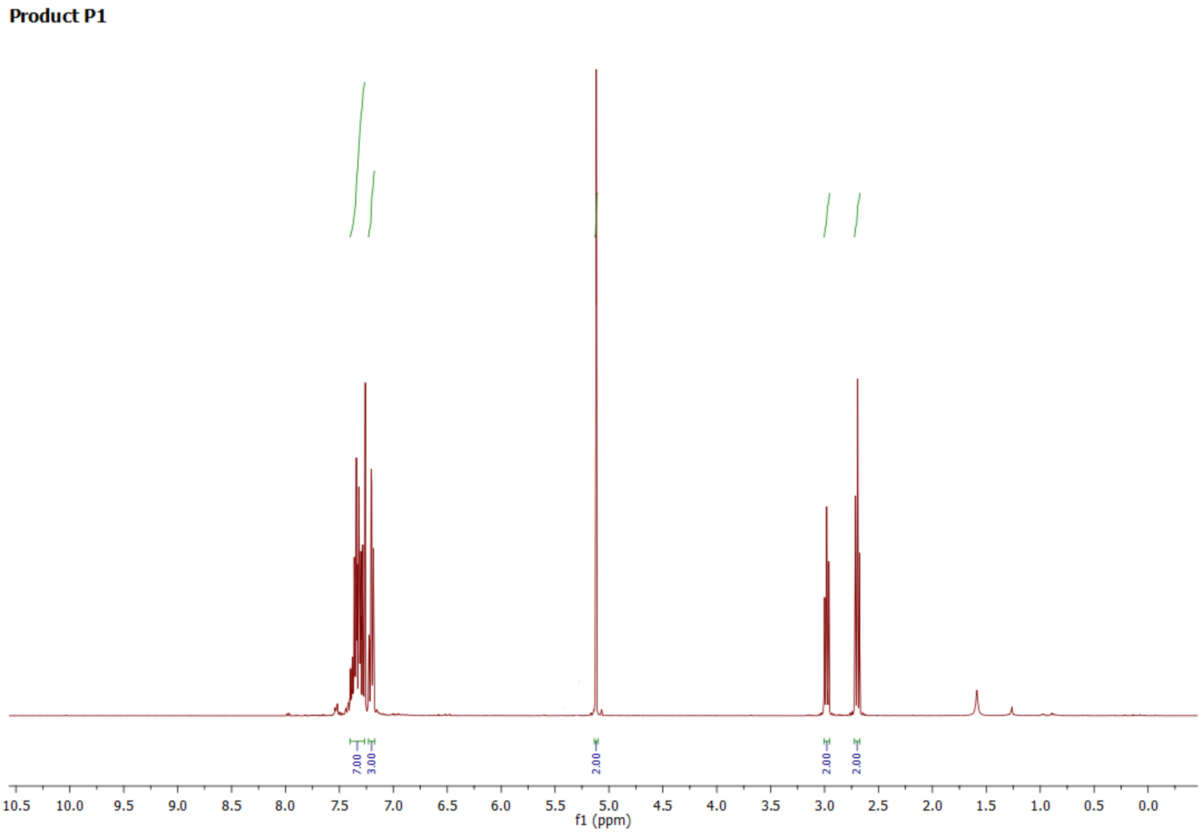

Figure S2. ^{1}H NMR (400 MHz, $CDCl_3$) of product **P1**

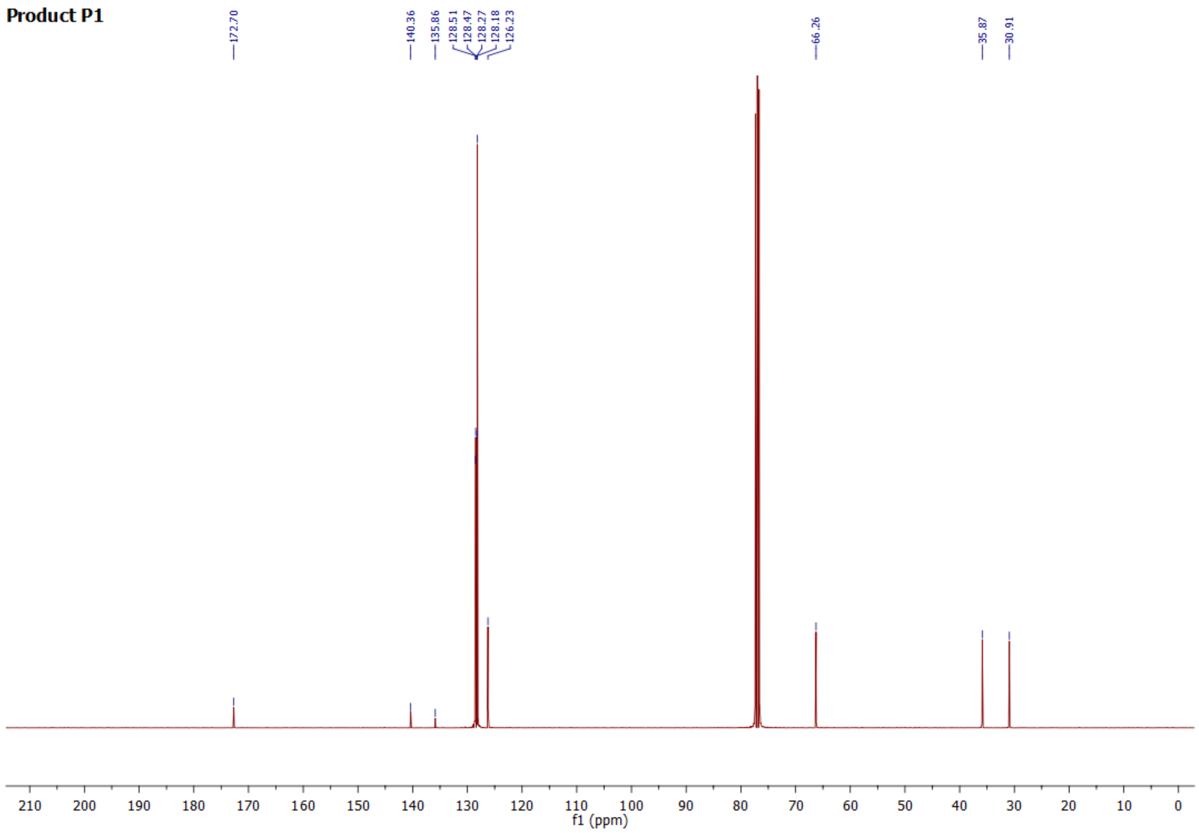

Figure S3. ^{13}C NMR (101 MHz, $CDCl_3$) of product **P1**

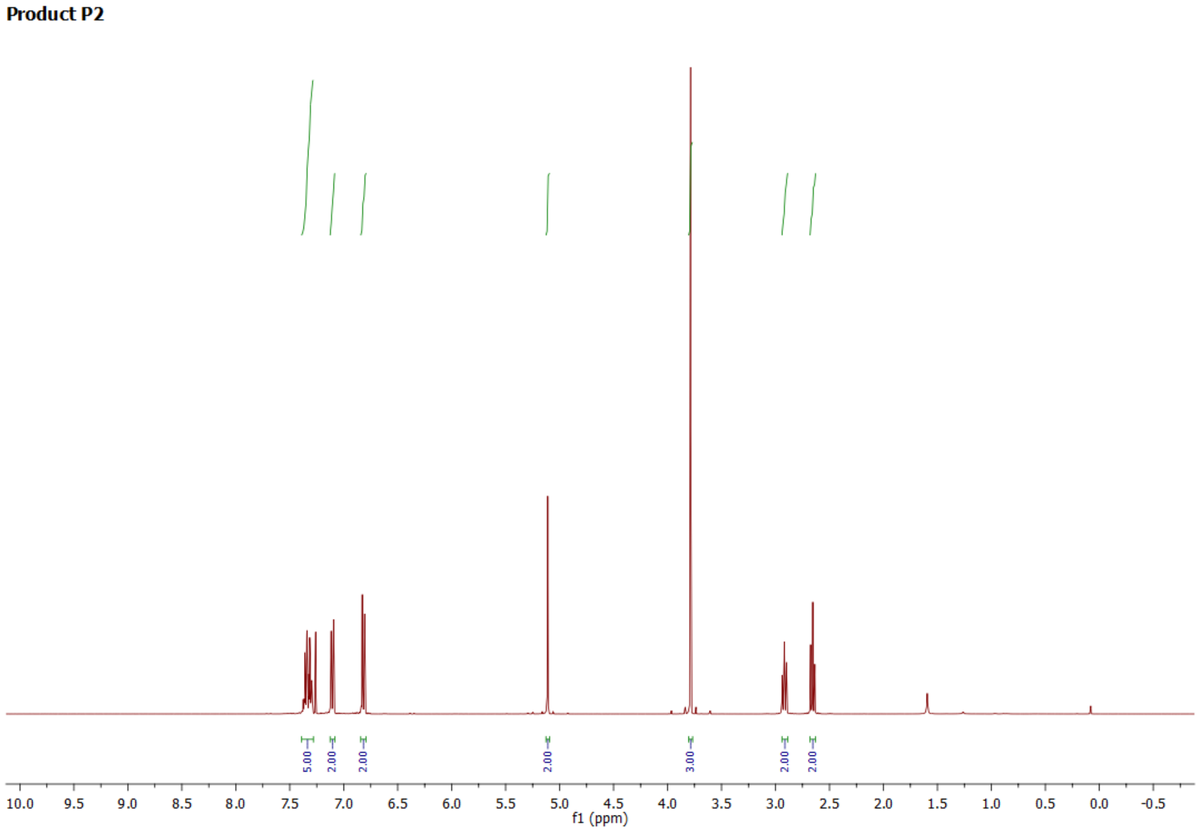

Figure S4. ^{1}H NMR (400 MHz, $CDCl_3$) of product **P2**

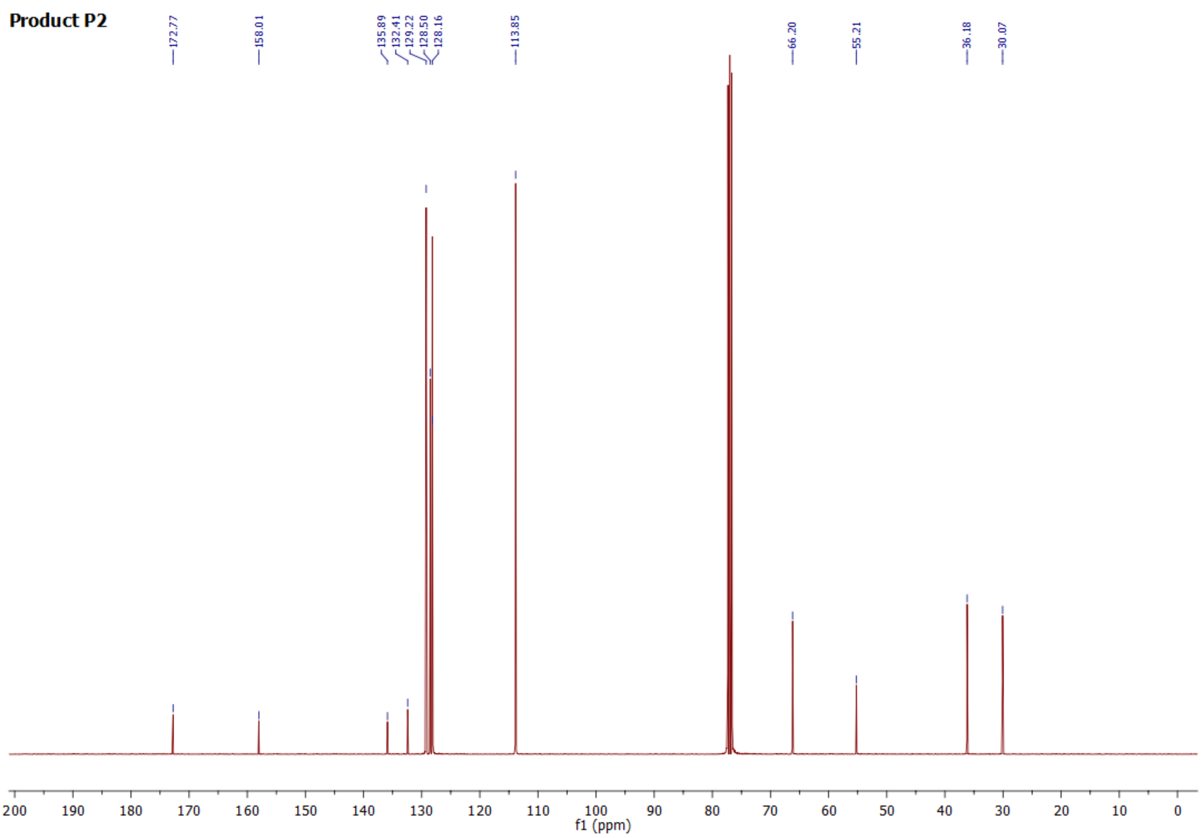

Figure S5. ^{13}C NMR (101 MHz, $CDCl_3$) of product **P2**

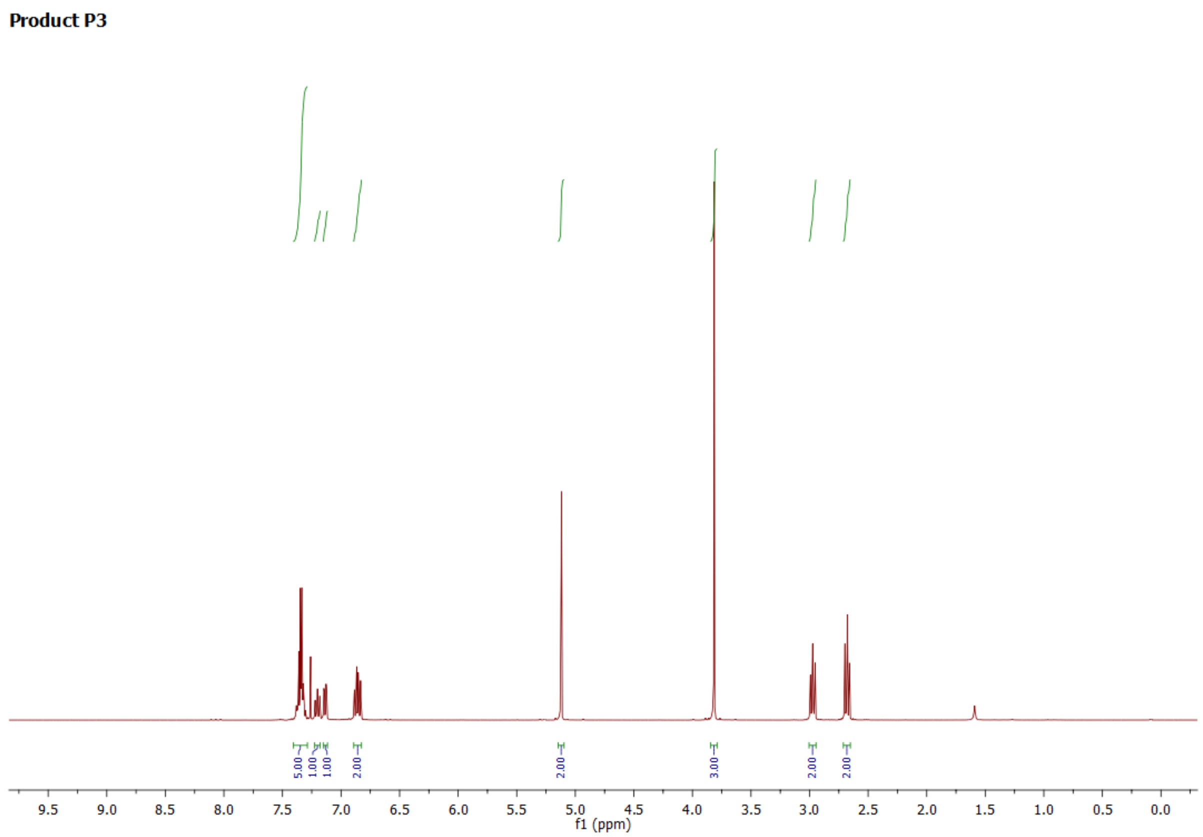

Figure S6. ^{1}H NMR (400 MHz, $CDCl_3$) of product **P3**

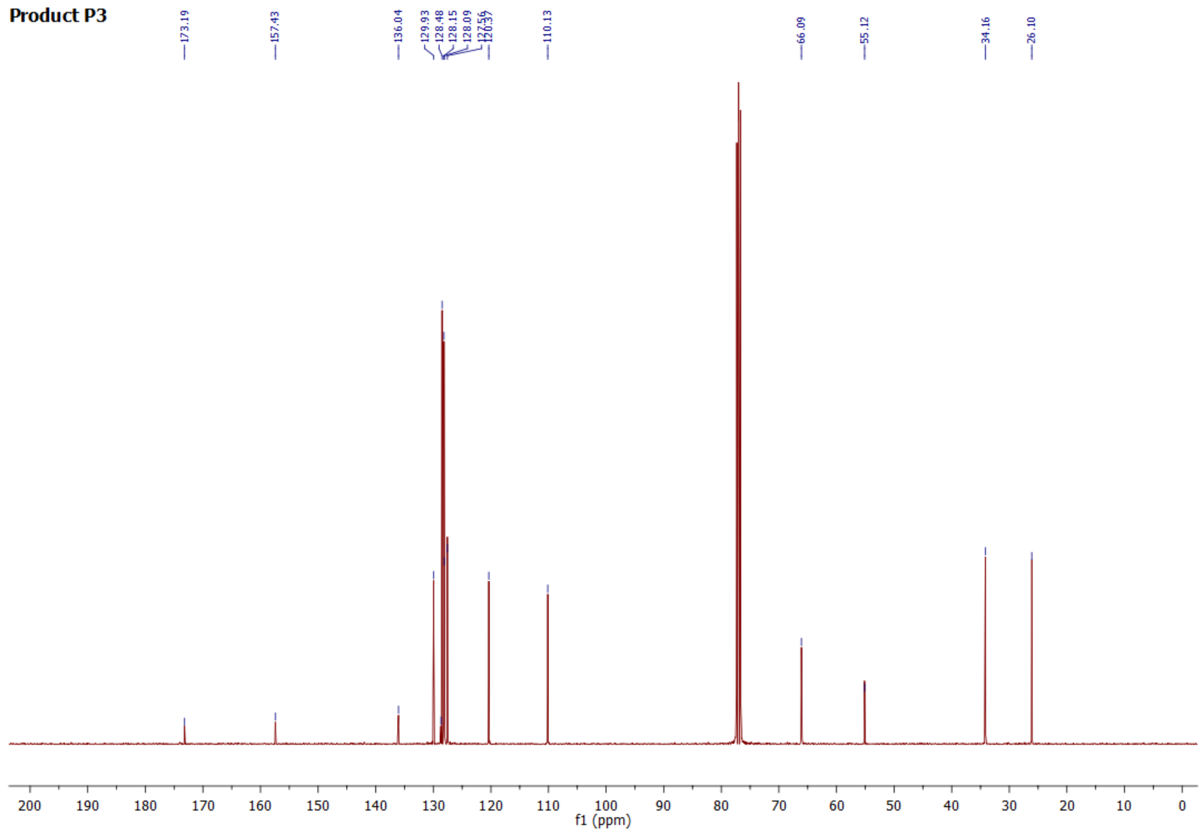

Figure S7. ^{13}C NMR (101 MHz, $CDCl_3$) of product **P3**

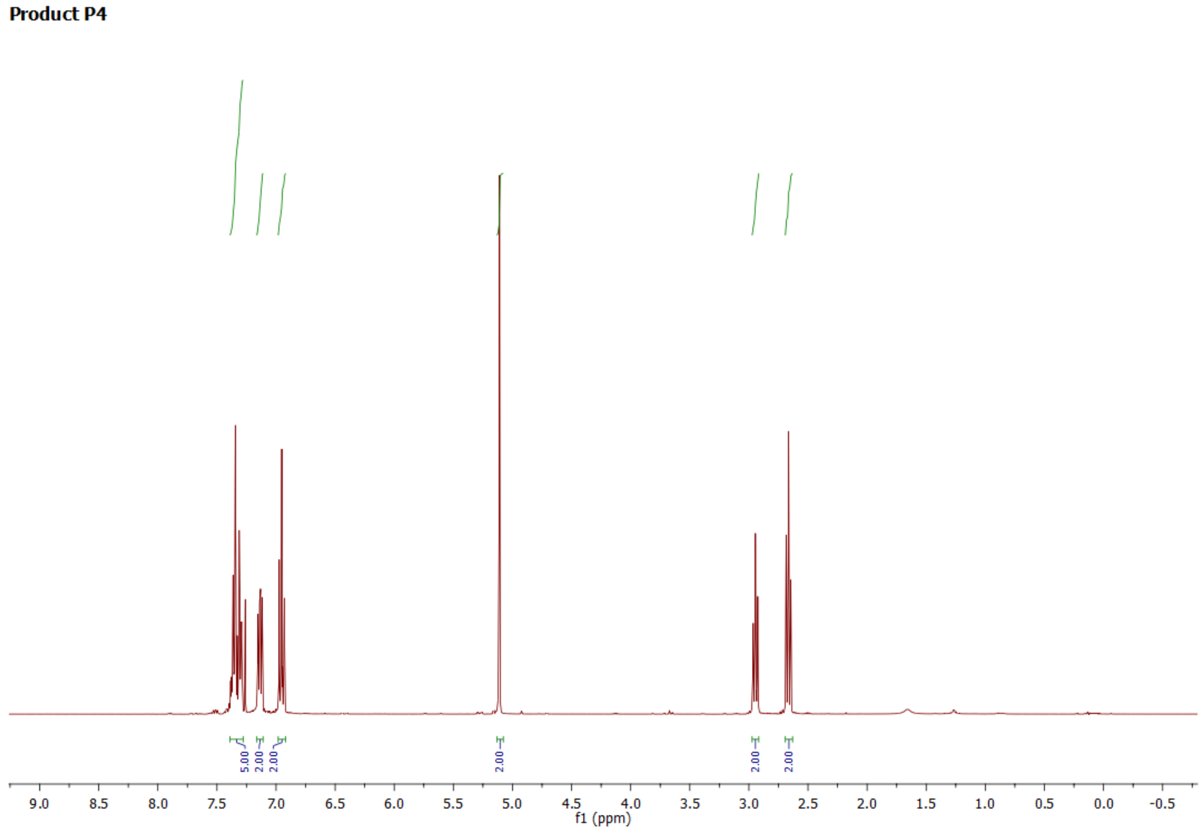

Figure S8. ^{1}H NMR (400 MHz, $CDCl_3$) of product **P4**

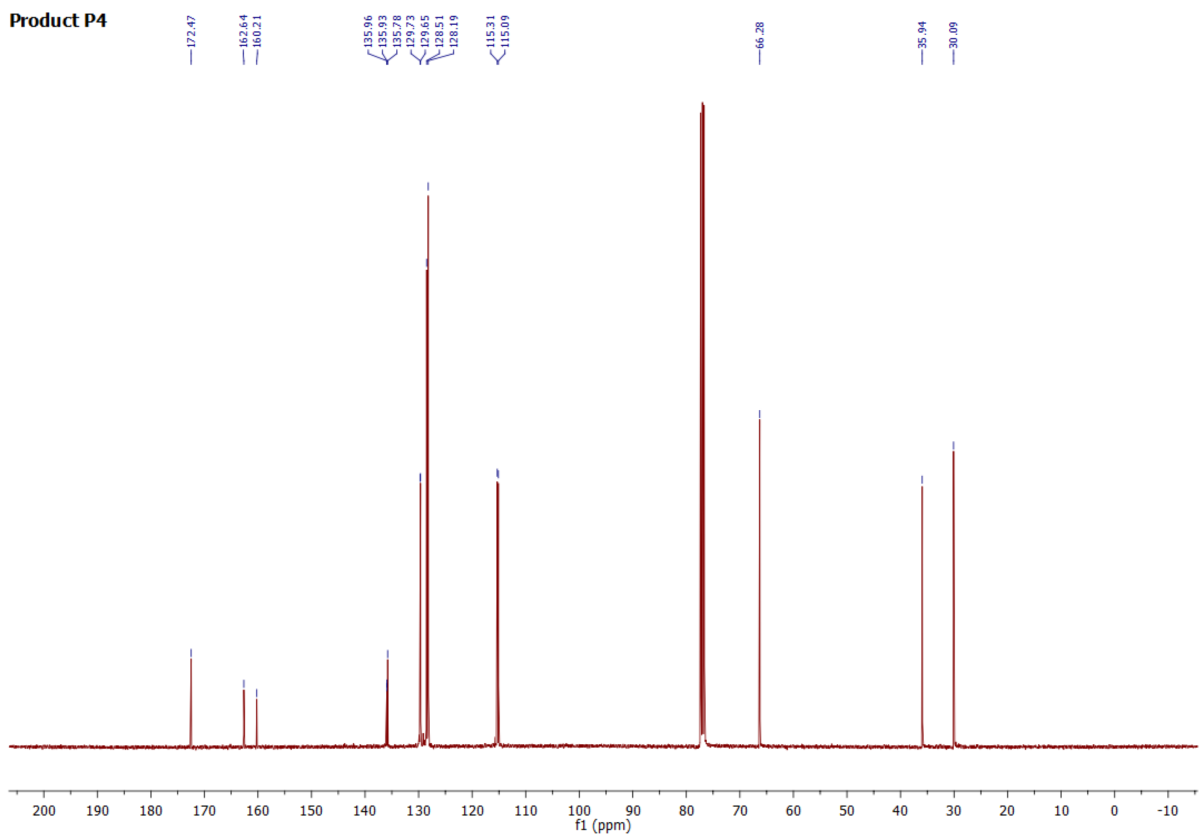

Figure S9. ^{13}C NMR (101 MHz, $CDCl_3$) of product **P4**

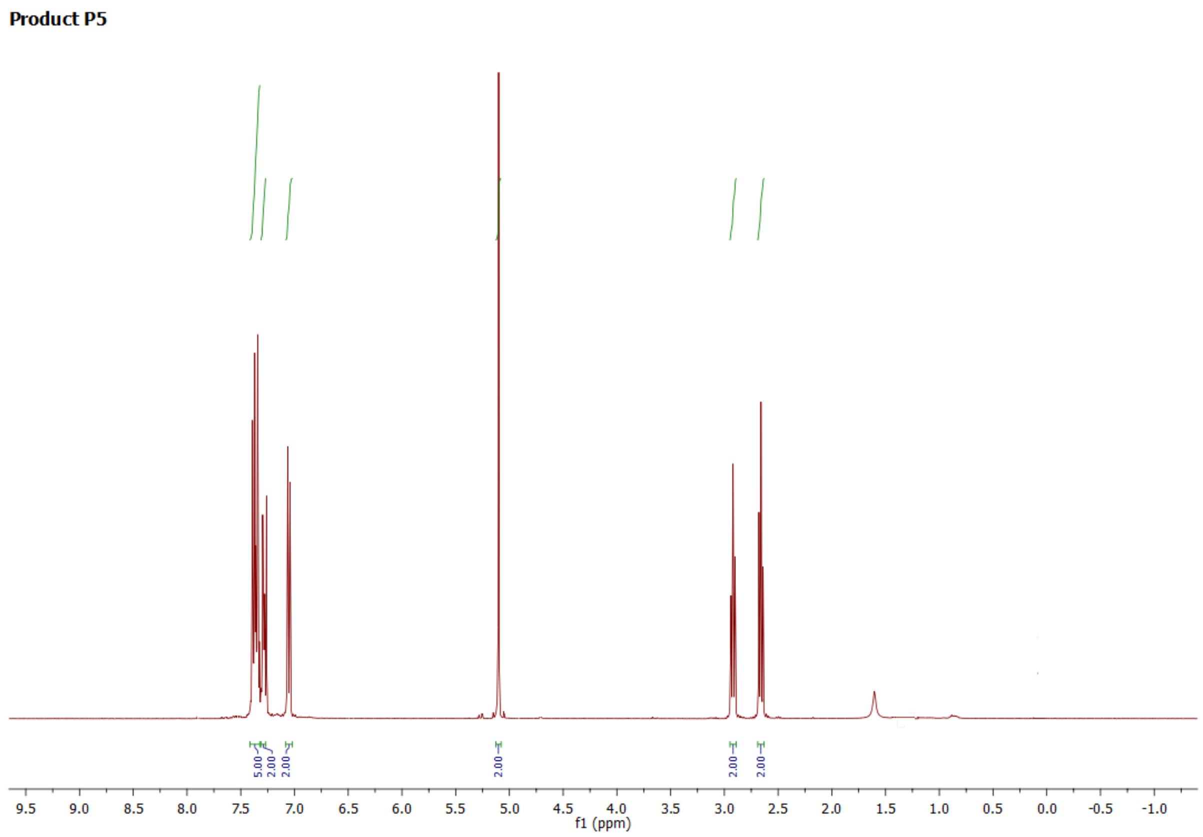

Figure S10. ^{1}H NMR (400 MHz, $CDCl_3$) of product **P5**

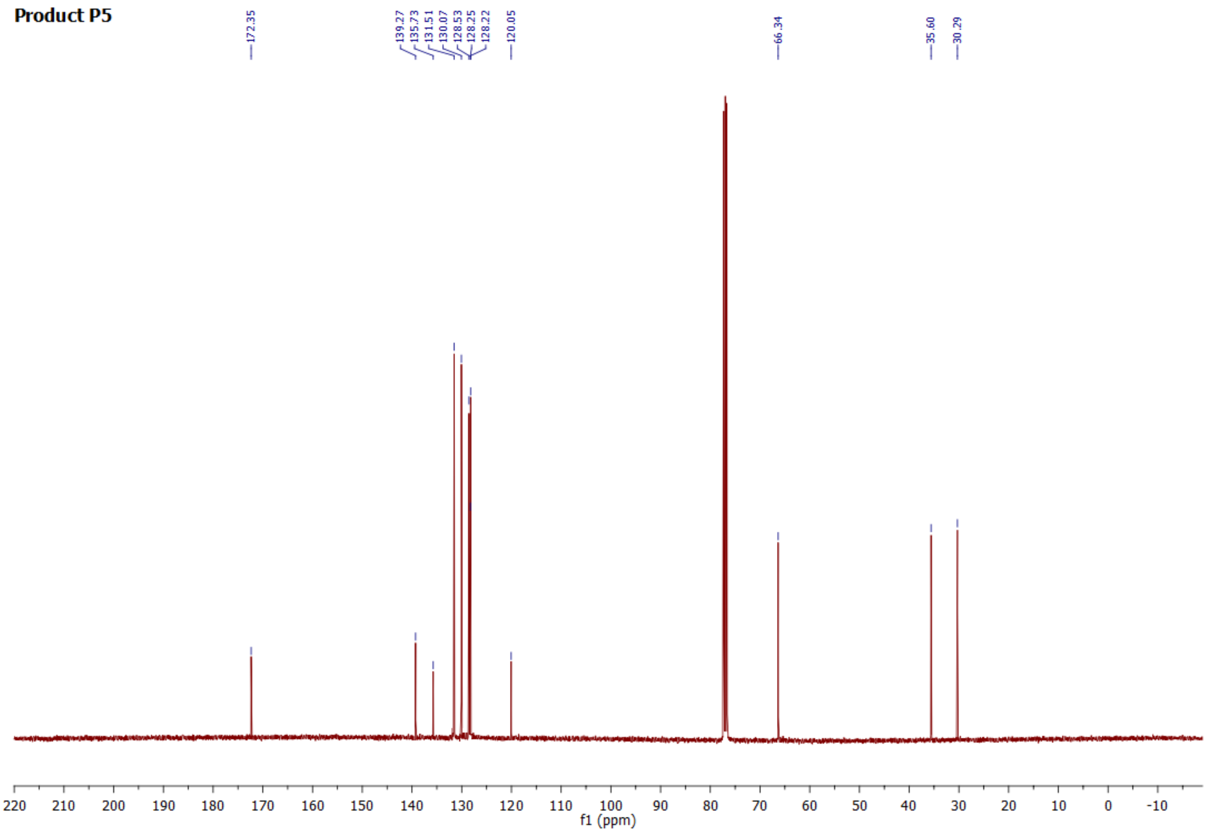

Figure S11. ^{13}C NMR (101 MHz, $CDCl_3$) of product **P5**

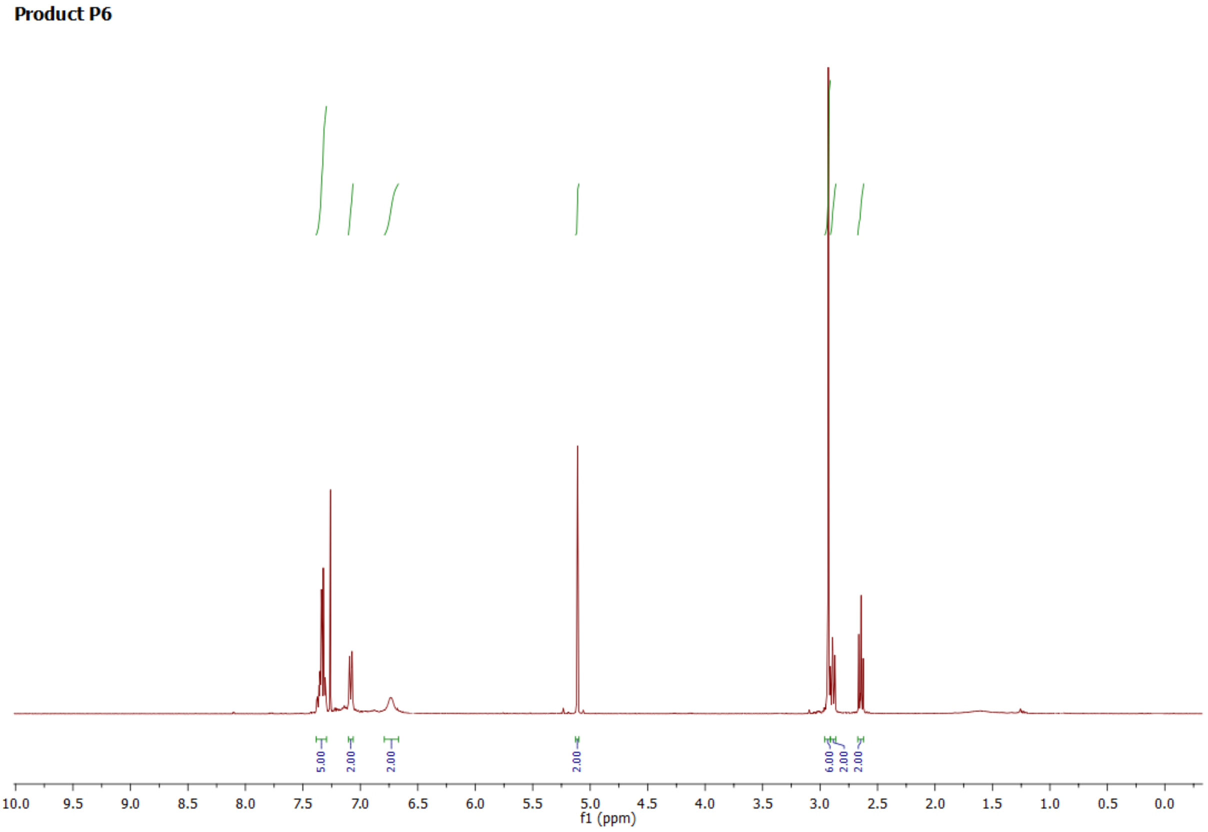

Figure S12. ^{1}H NMR (400 MHz, $CDCl_3$) of product **P6**

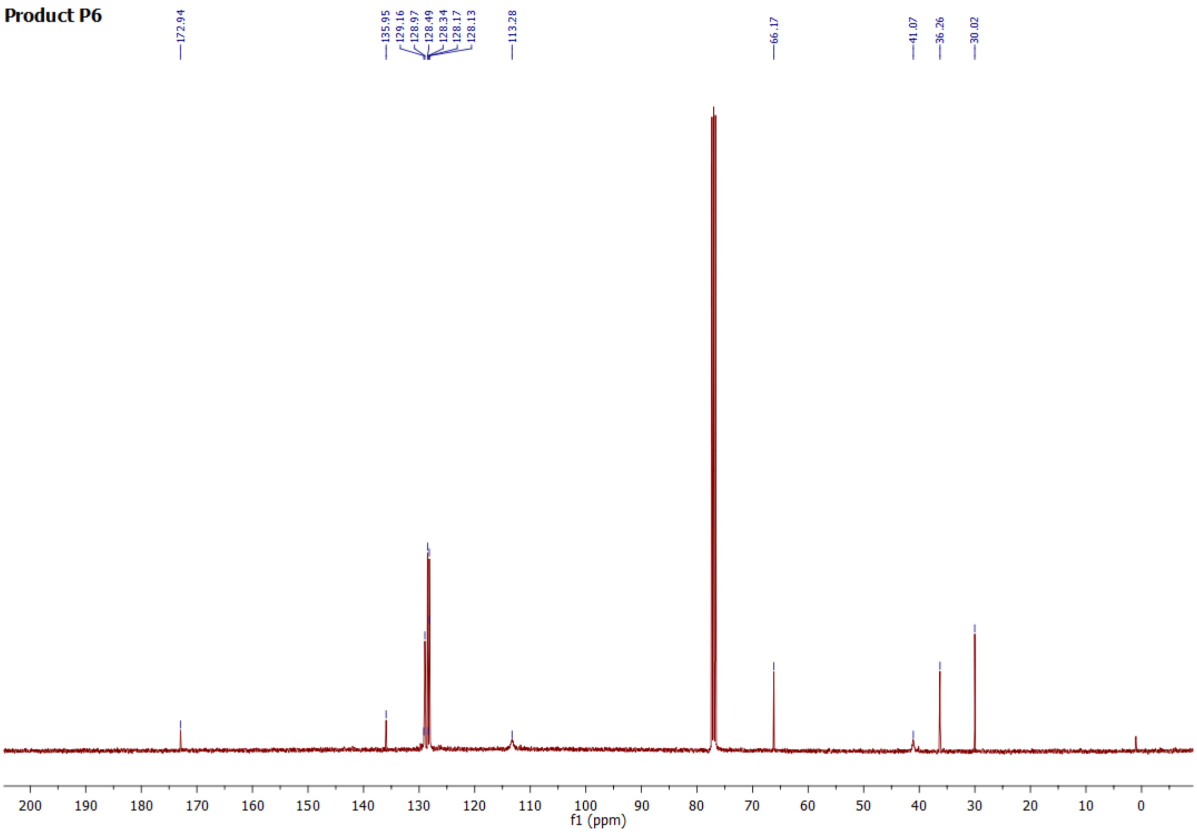

Figure S13. ^{13}C NMR (101 MHz, $CDCl_3$) of product **P6**

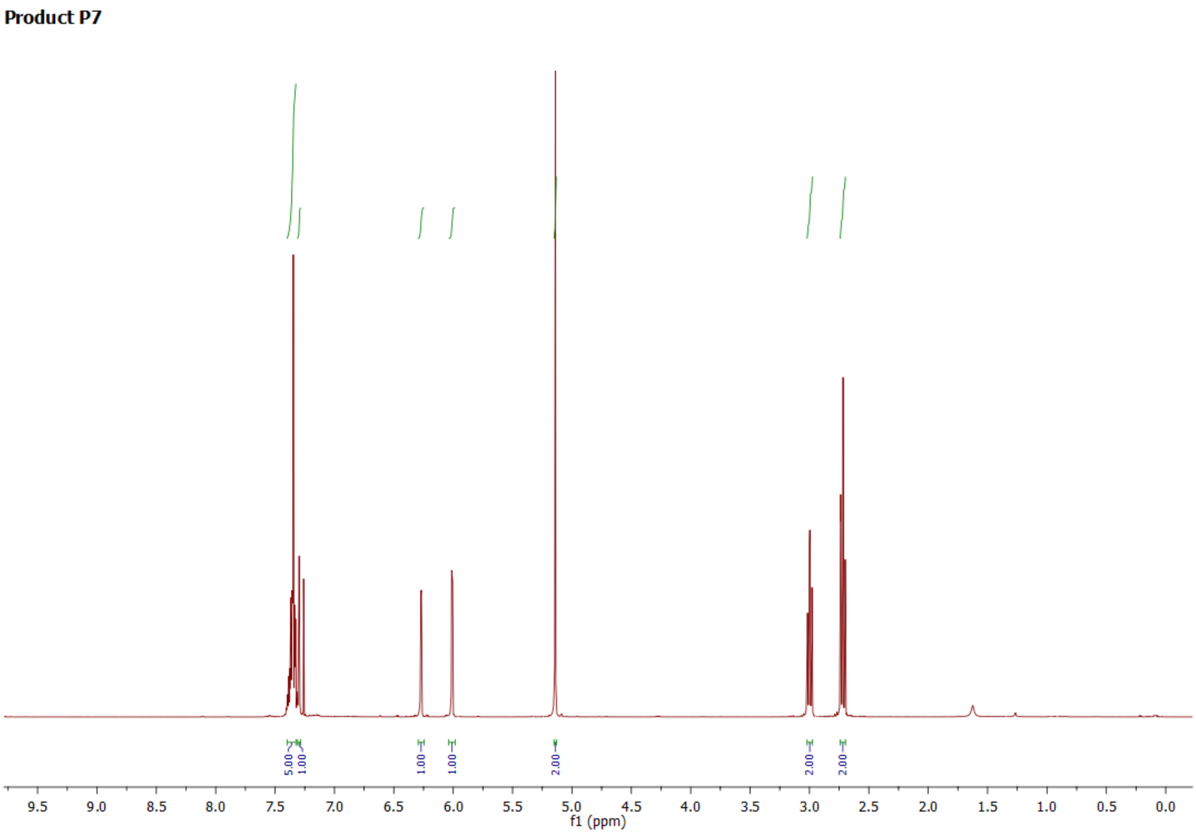

Figure S14. ^{1}H NMR (400 MHz, $CDCl_3$) of product **P7**

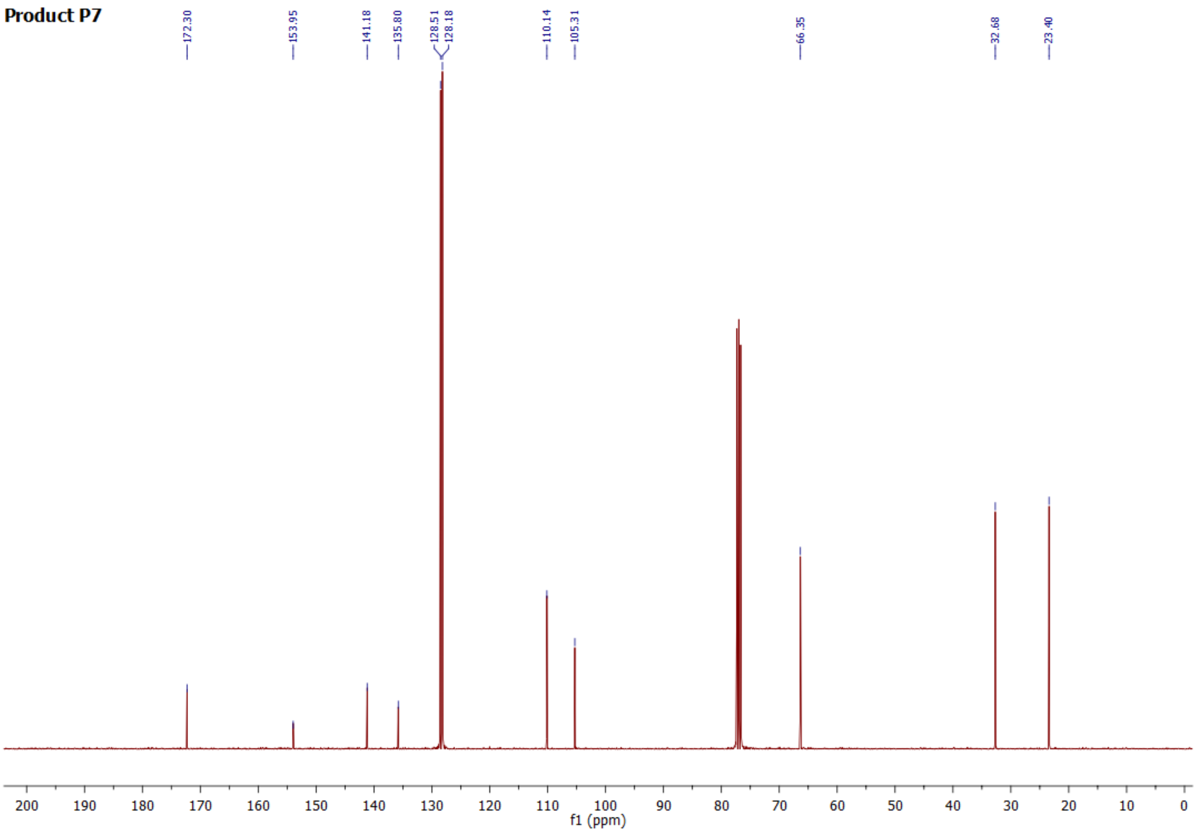

Figure S15. ^{13}C NMR (101 MHz, $CDCl_3$) of product **P7**

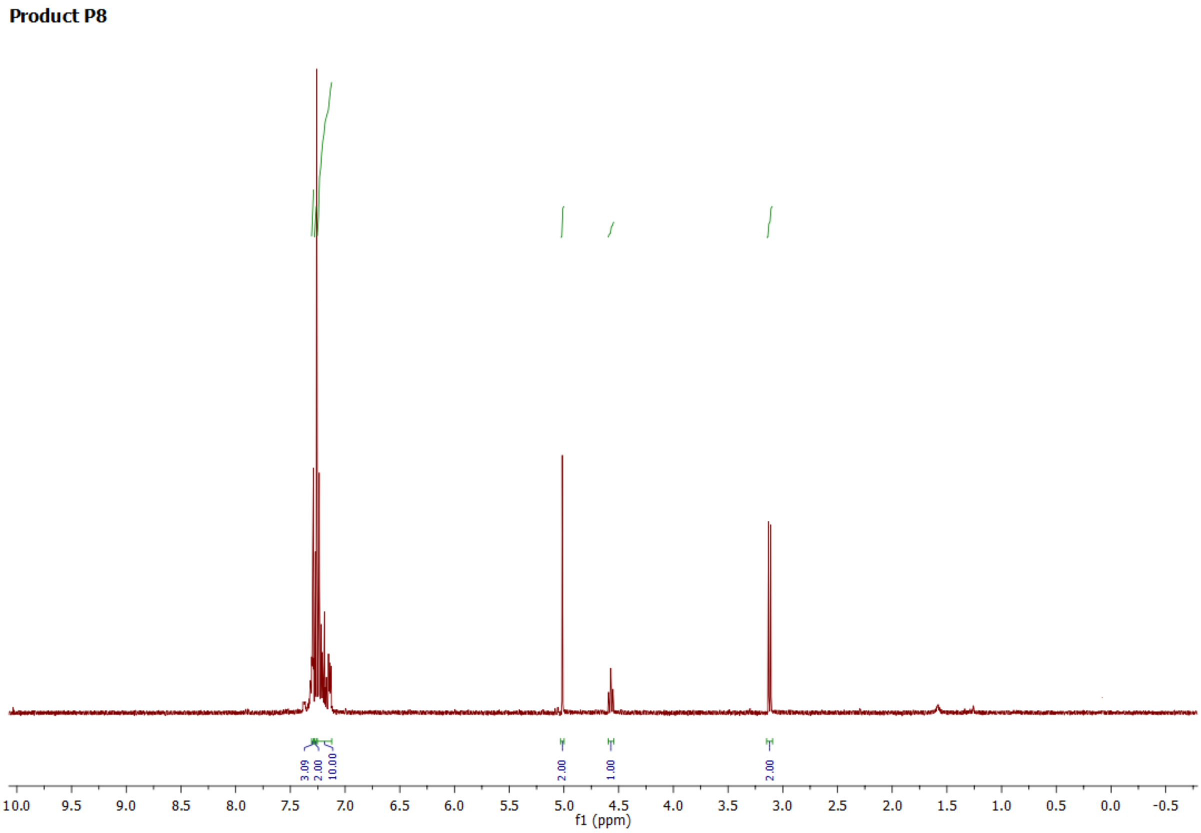

Figure S16. ^{1}H NMR (400 MHz, $CDCl_3$) of product **P8**

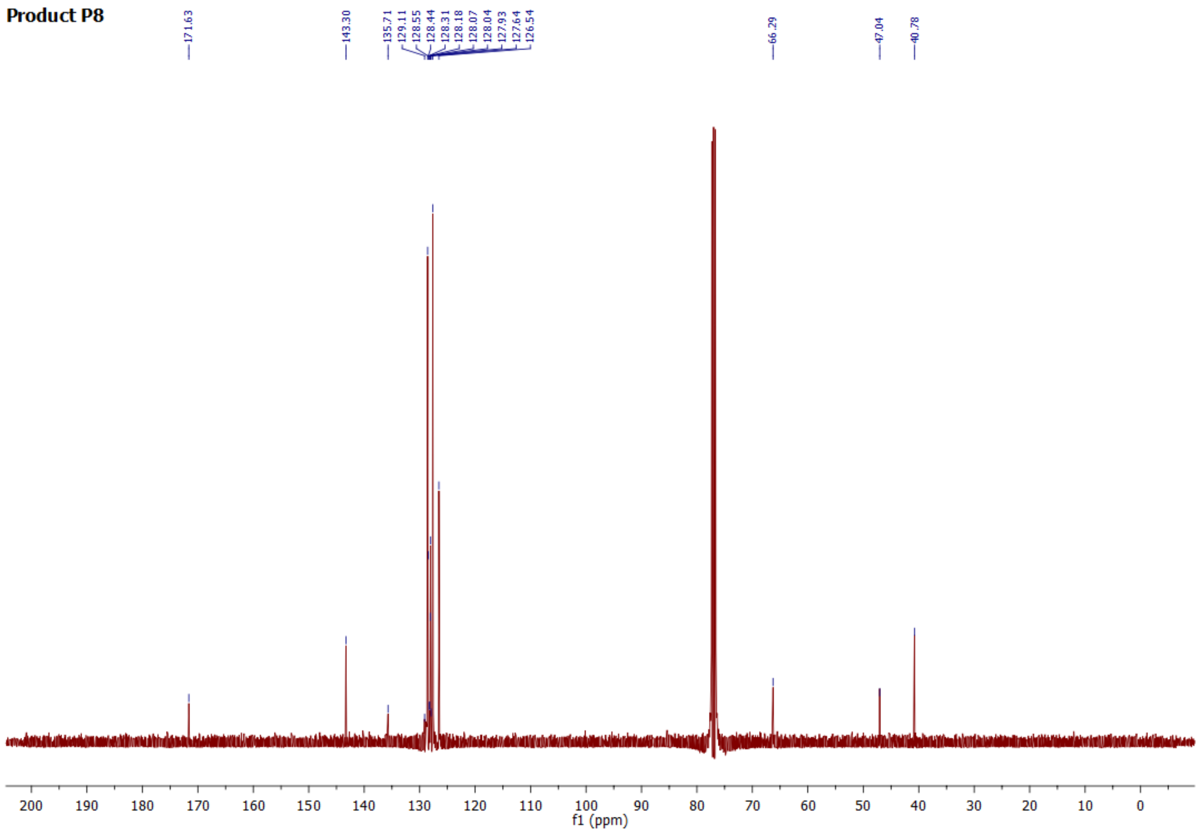

Figure S17. ^{13}C NMR (101 MHz, $CDCl_3$) of product **P8**

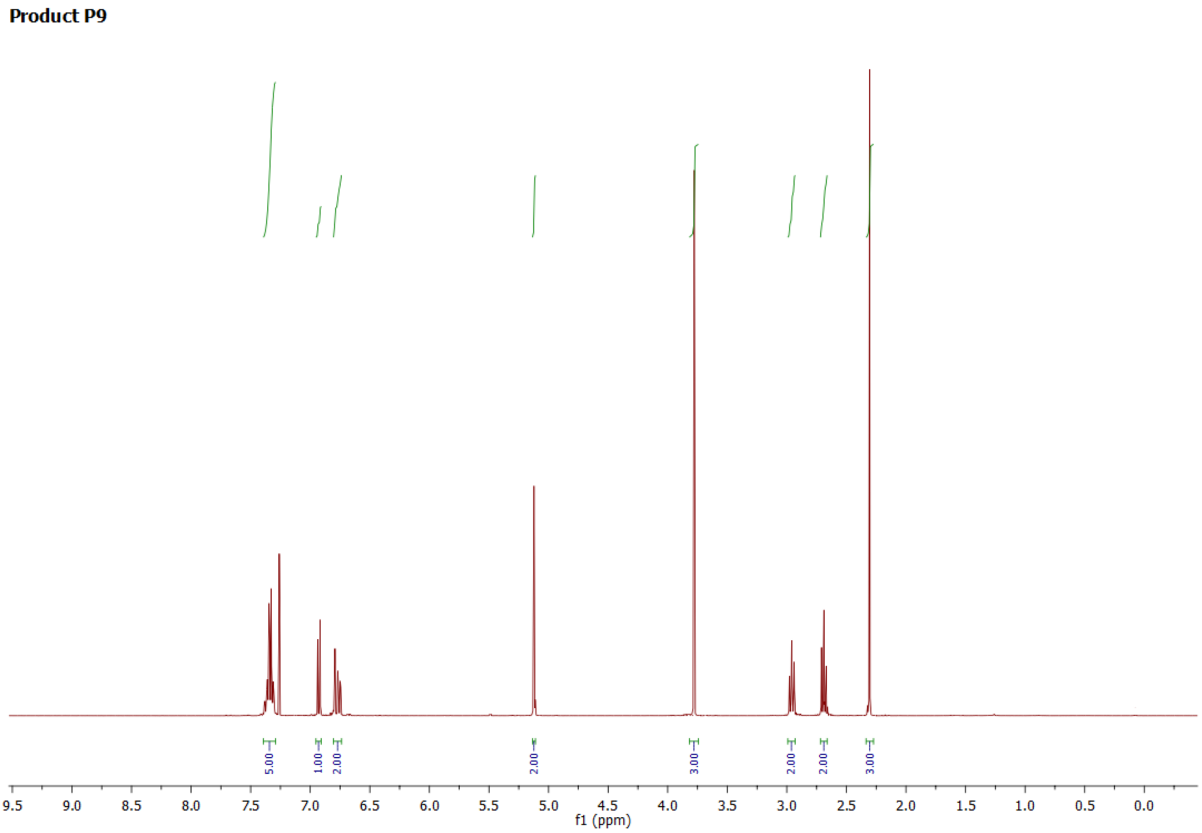

Figure S18. ^{1}H NMR (400 MHz, $CDCl_3$) of product **P9**

Product P9

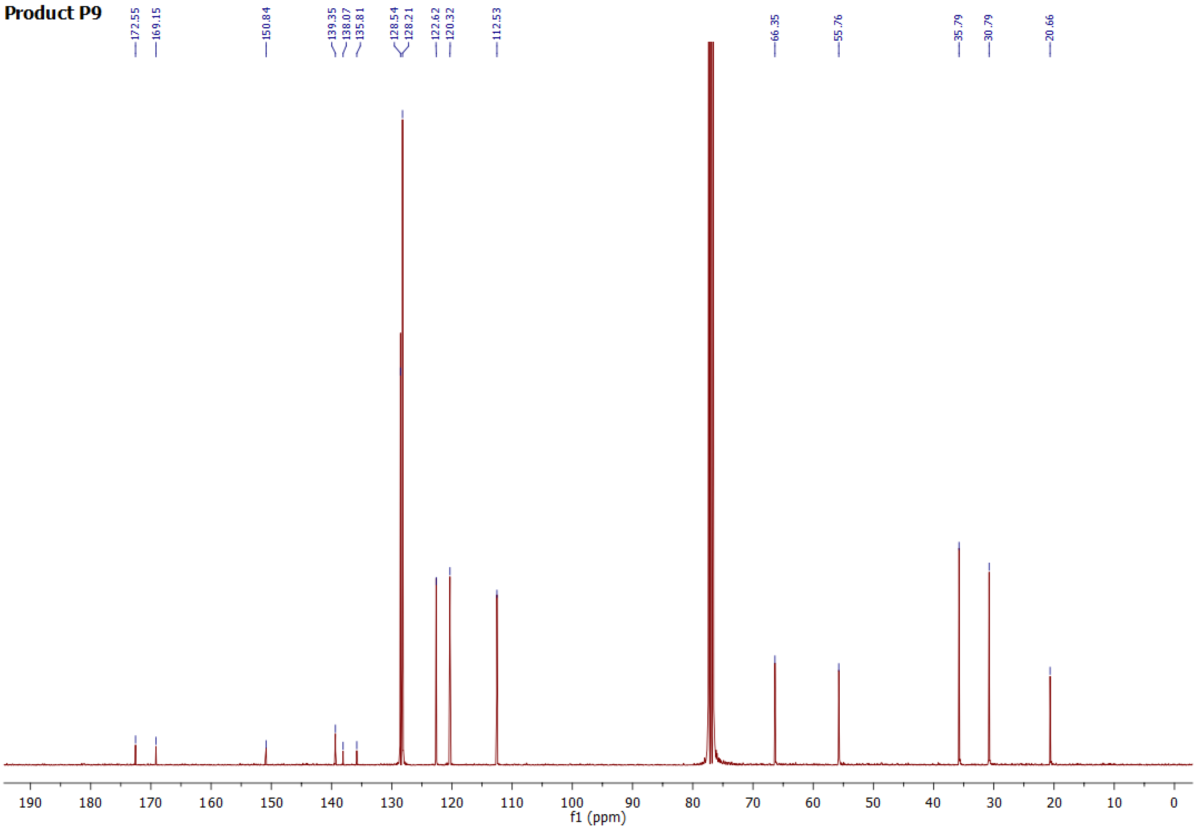

Figure S19. ^{13}C NMR (101 MHz, $CDCl_3$) of product **P9**

Product P10

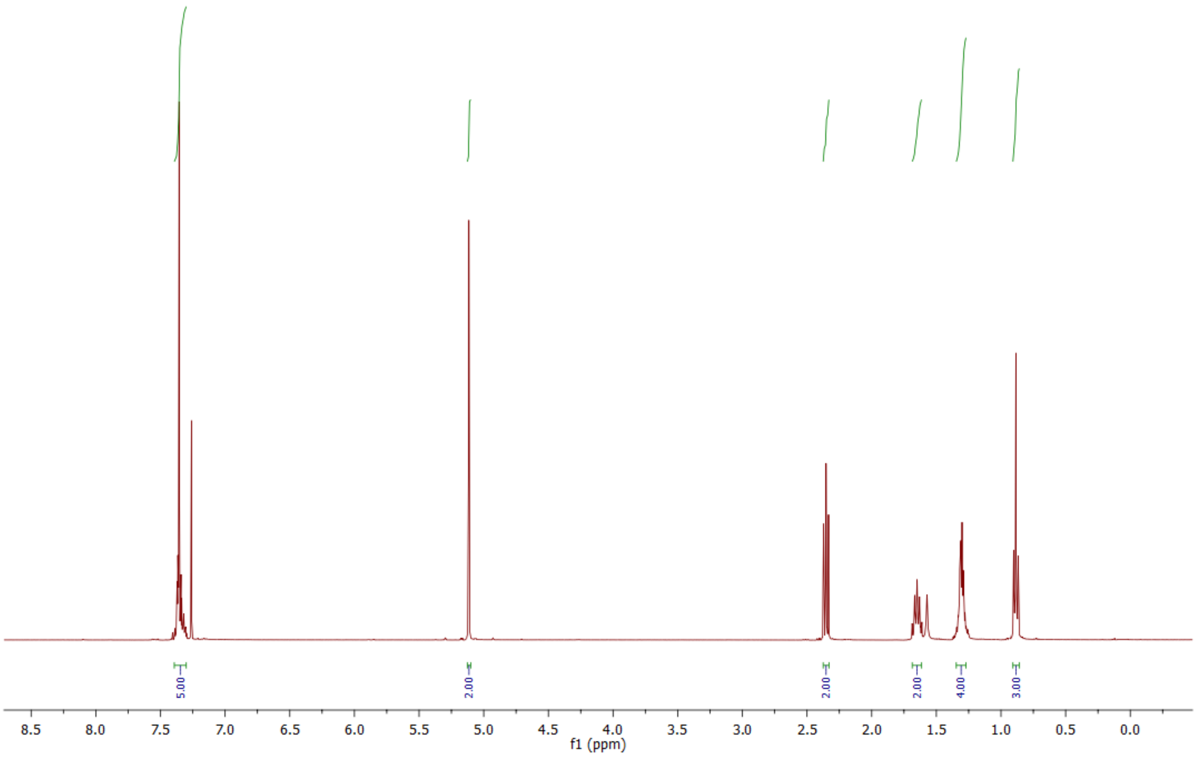

Figure S20. ^{1}H NMR (400 MHz, $CDCl_3$) of product **P10**

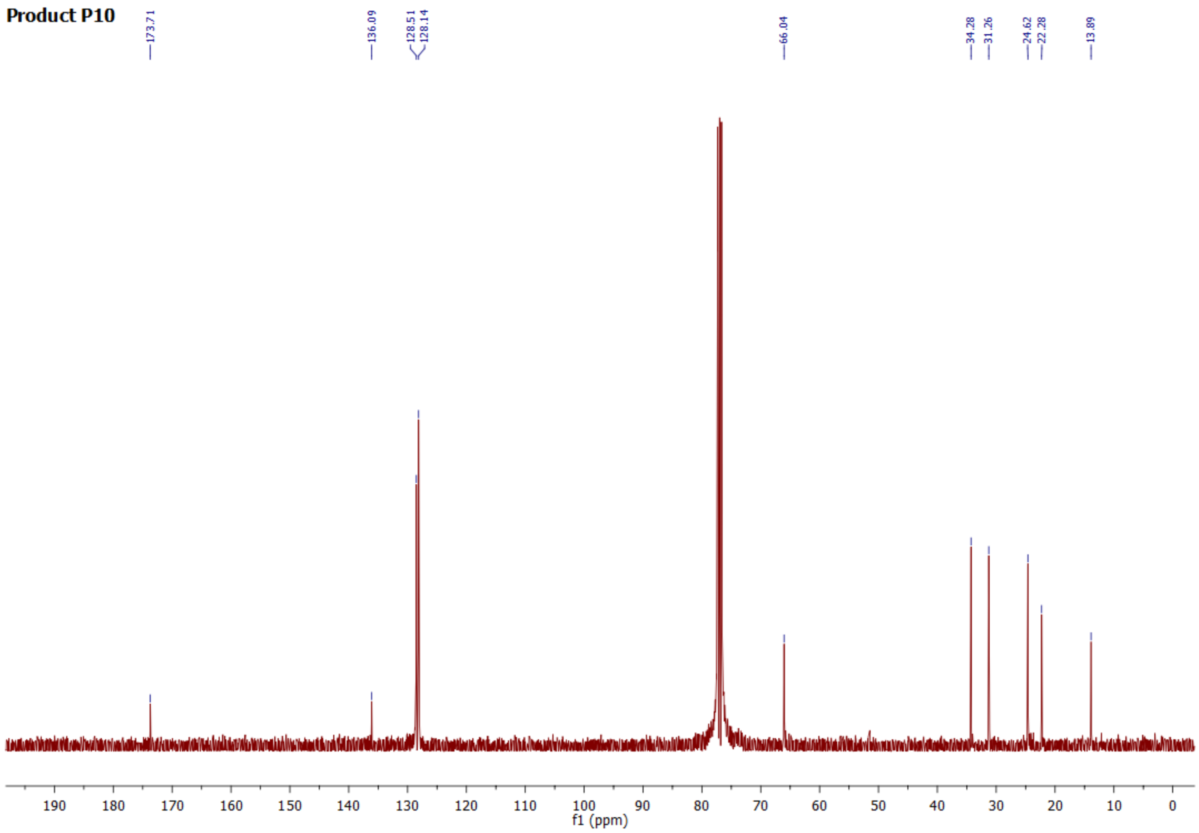

Figure S21. ^{13}C NMR (101 MHz, $CDCl_3$) of product **P10**

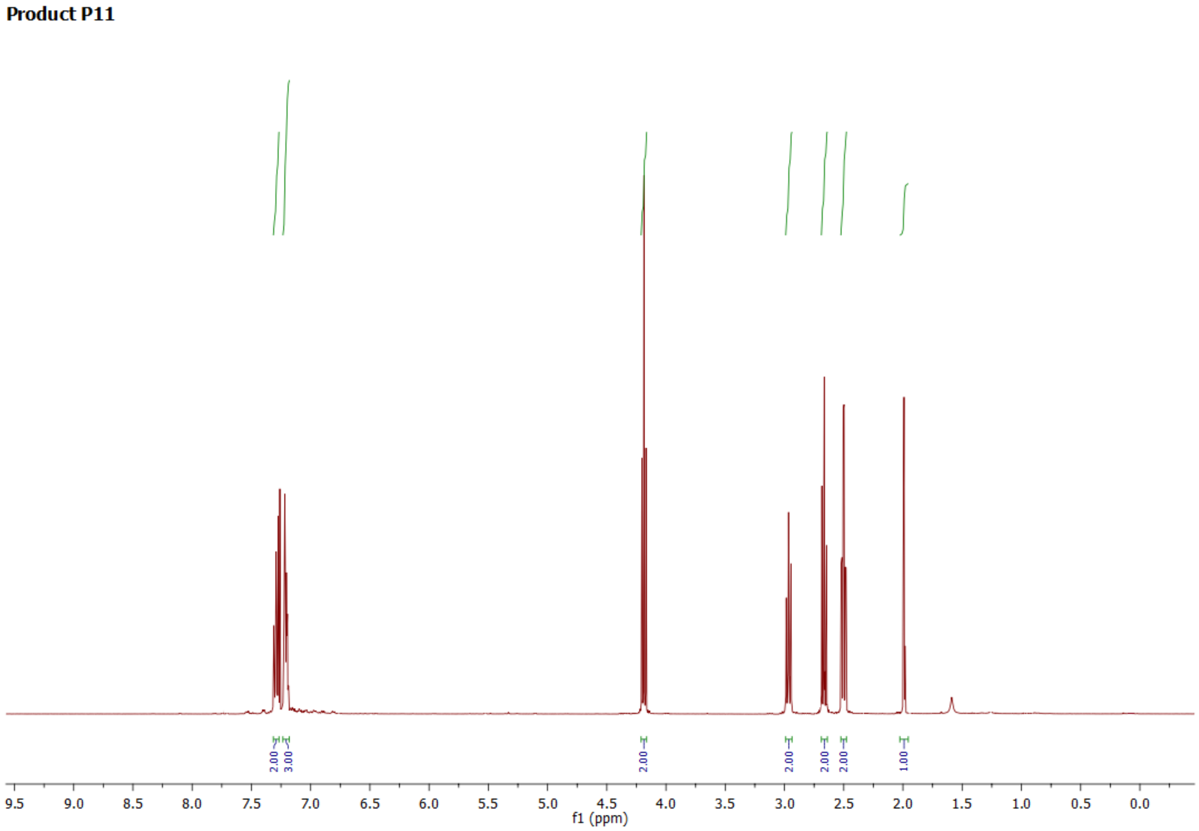

Figure S22. ^{1}H NMR (400 MHz, $CDCl_3$) of product **P11**

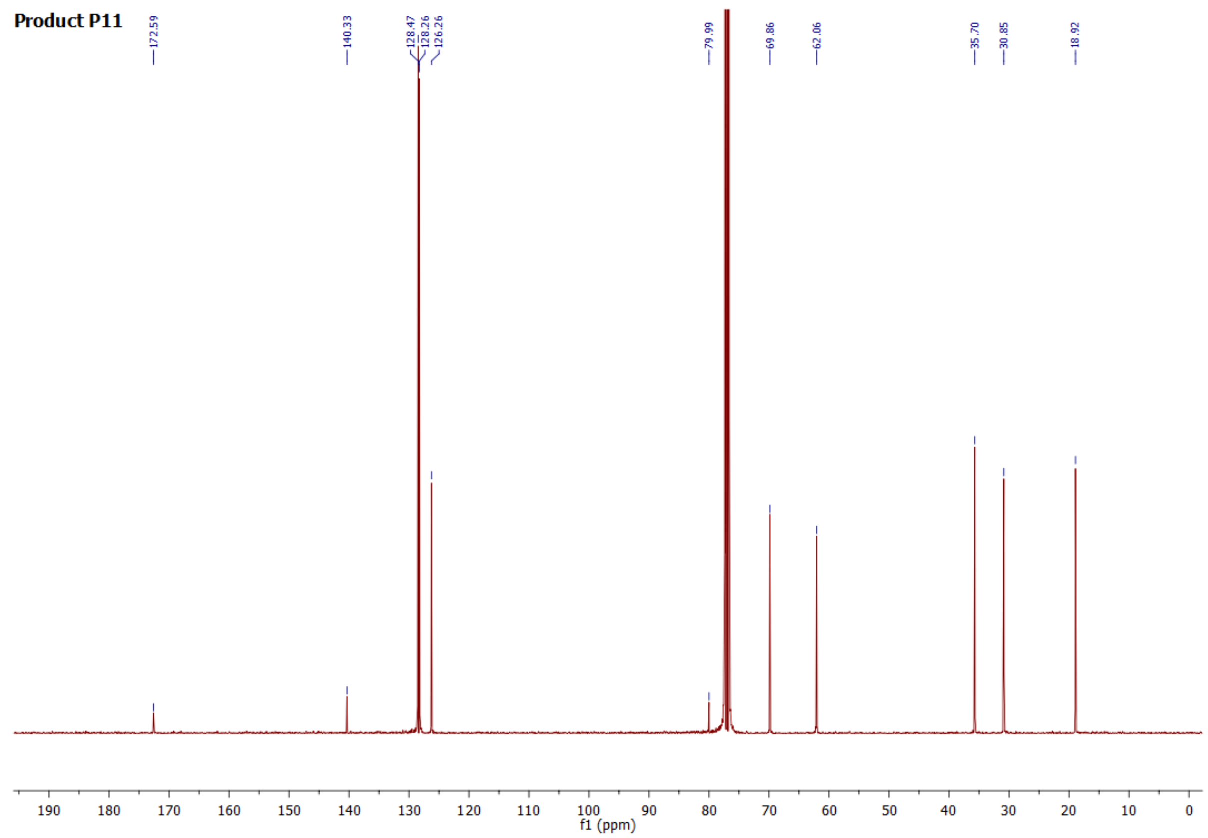

Figure S23. ^{13}C NMR (101 MHz, $CDCl_3$) of product **P11**

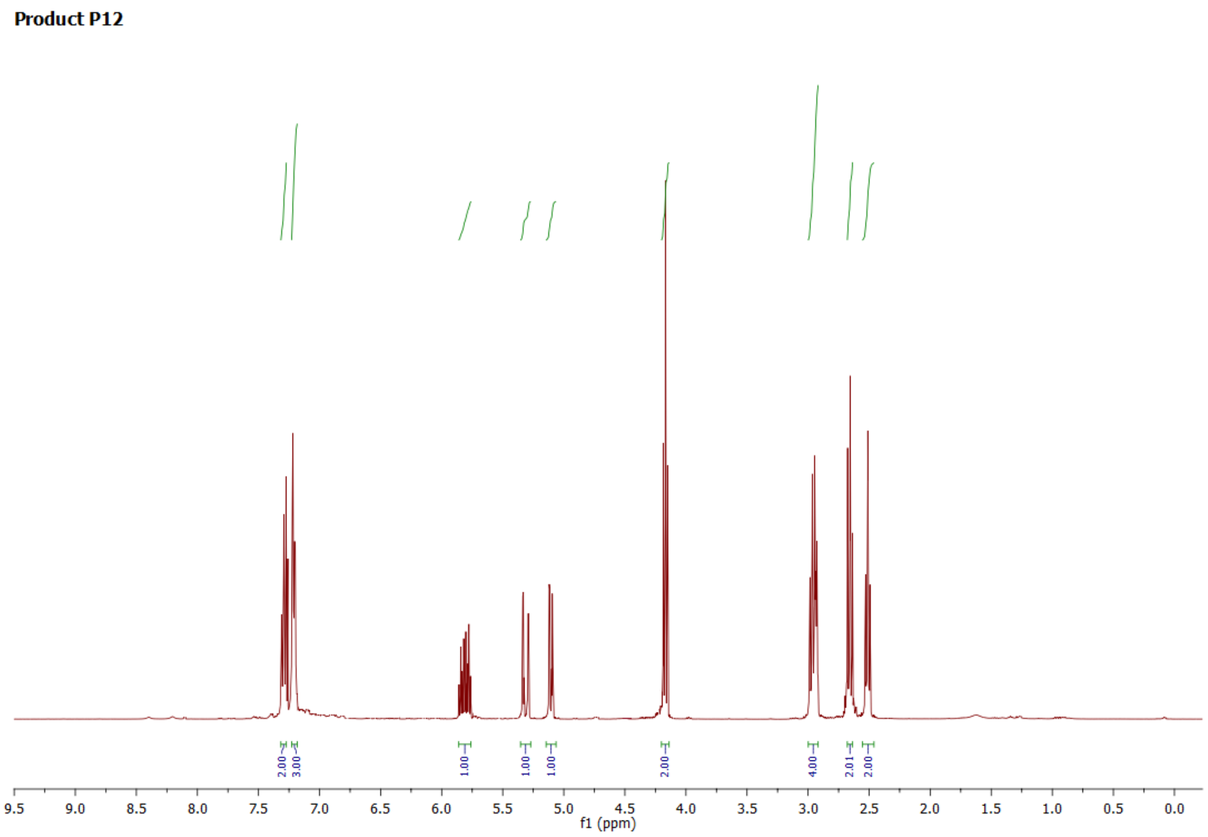

Figure S24. ^{1}H NMR (400 MHz, $CDCl_3$) of product **P12**

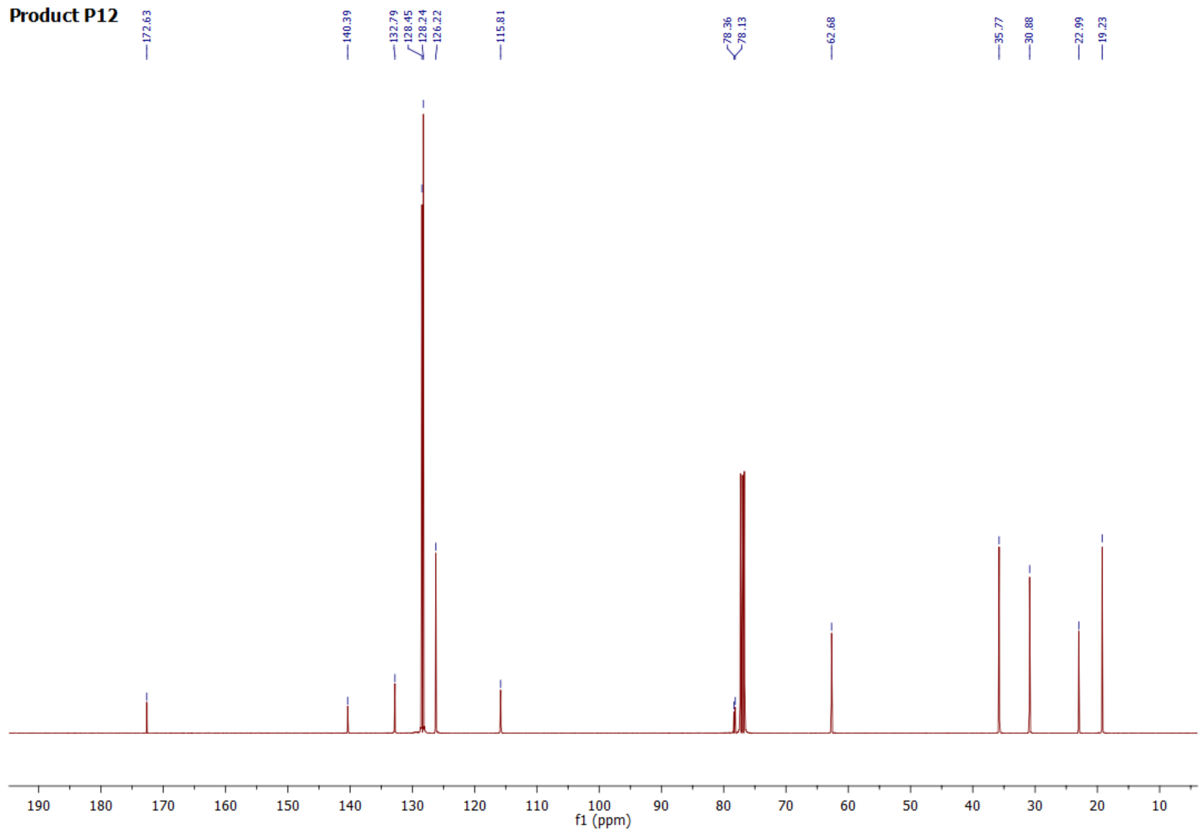

Figure S25. ^{13}C NMR (101 MHz, $CDCl_3$) of product **P12**

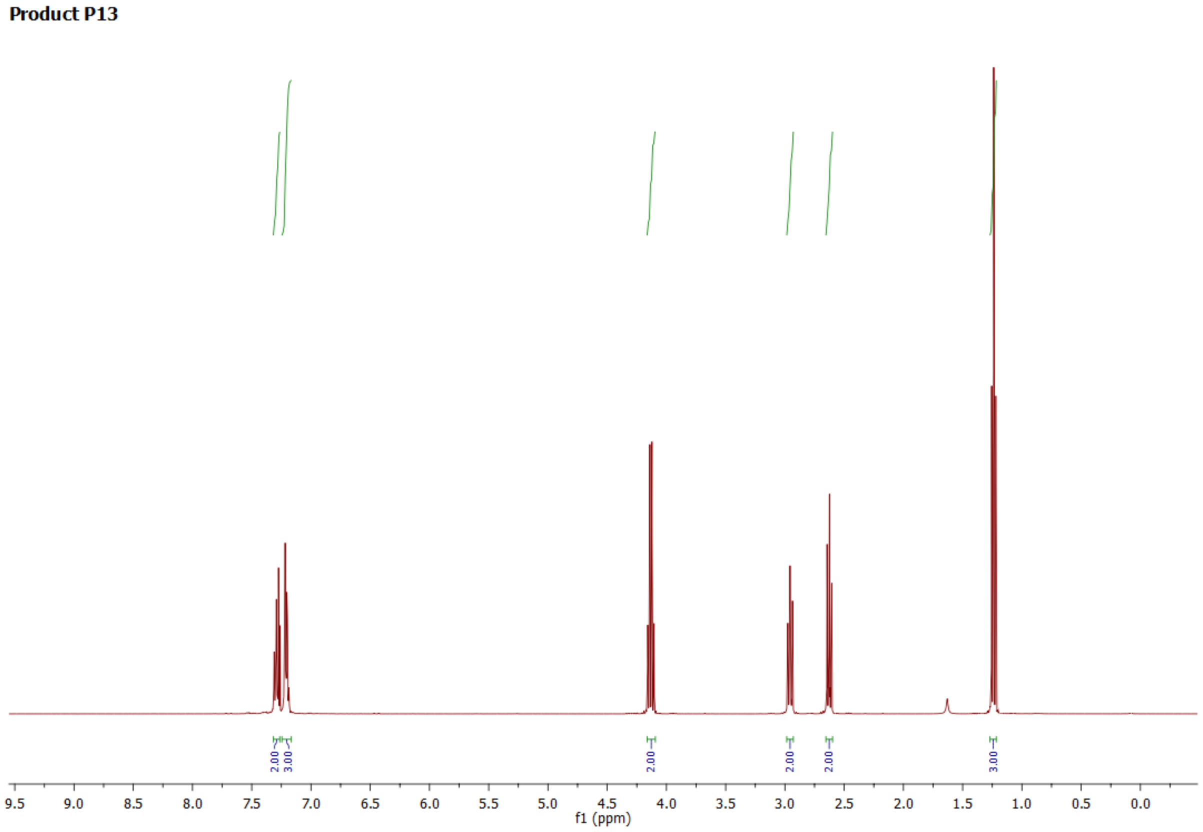

Figure S26. ^{1}H NMR (400 MHz, $CDCl_3$) of product **P13**

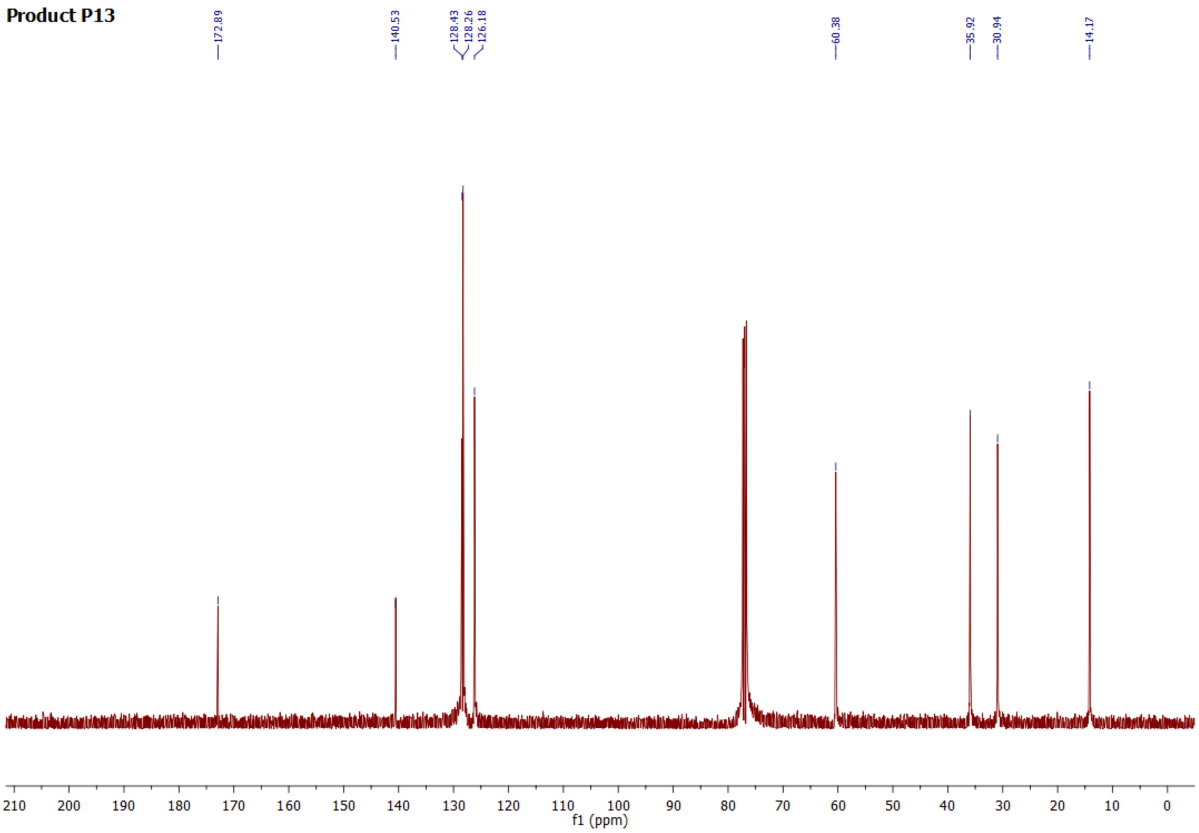

Figure S27. ^{13}C NMR (101 MHz, $CDCl_3$) of product **P13**

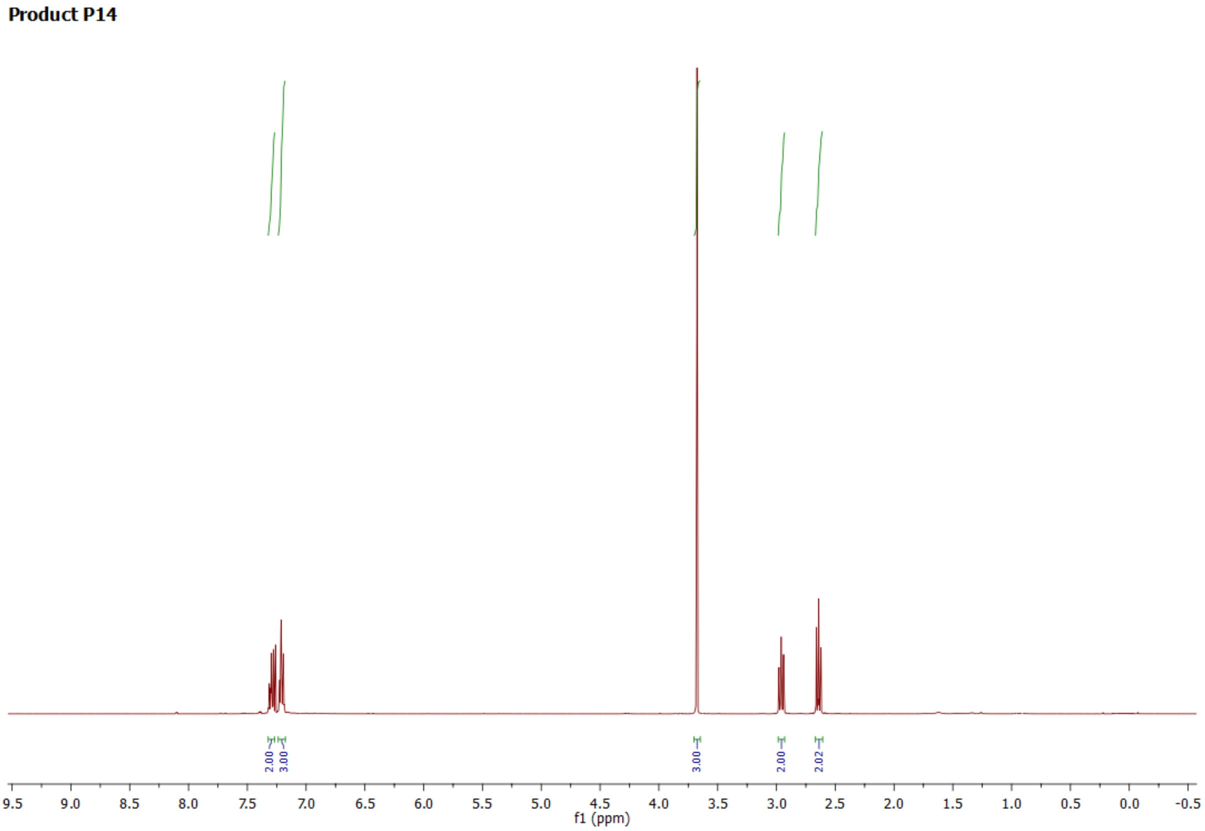

Figure S28. ^{1}H NMR (400 MHz, $CDCl_3$) of product **P14**

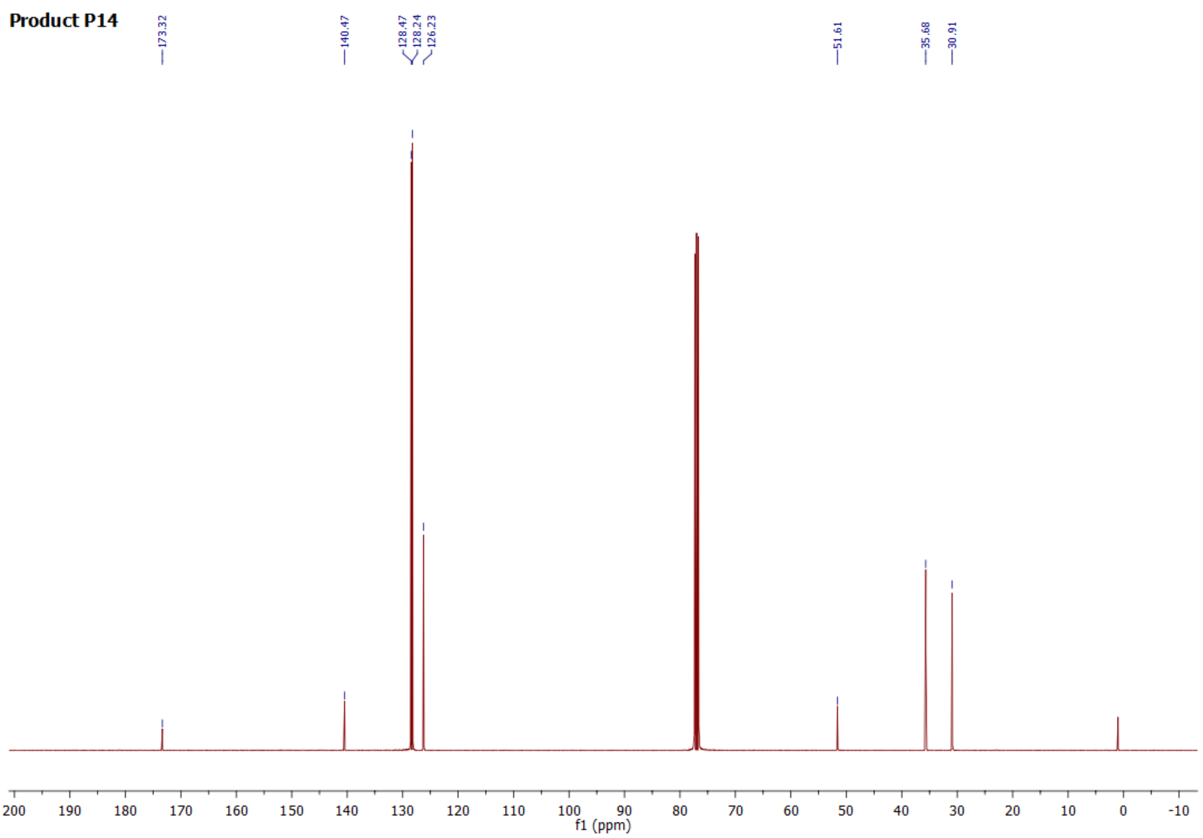

Figure S29. ^{13}C NMR (101 MHz, $CDCl_3$) of product **P14**

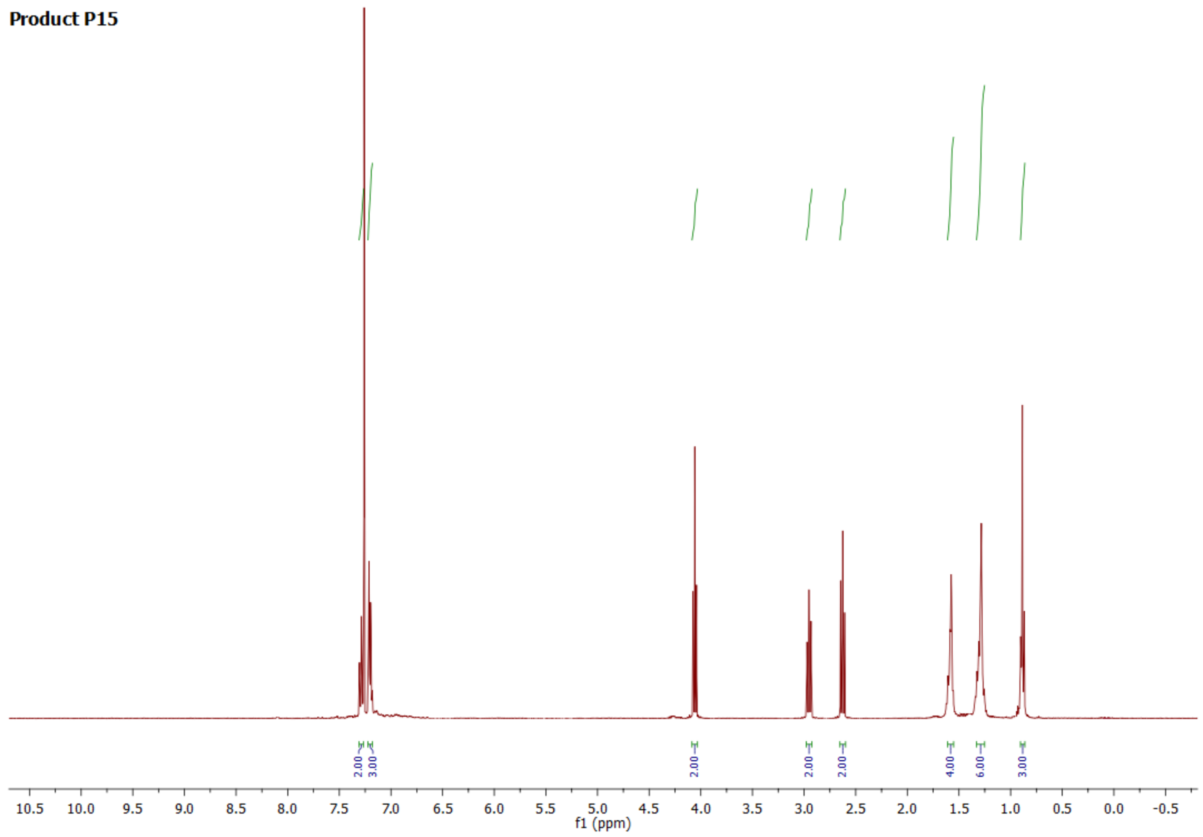

Figure S30. ^{1}H NMR (400 MHz, $CDCl_3$) of product **P15**

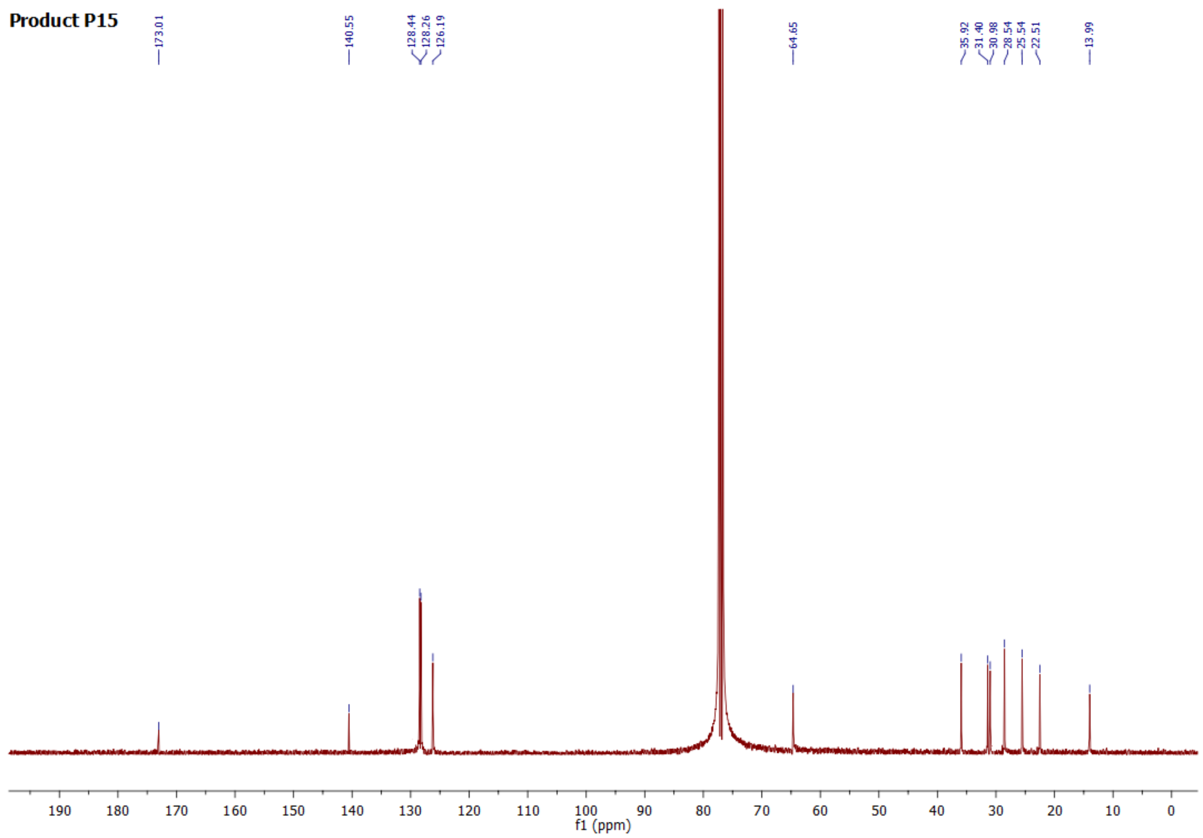

Figure S31. ^{13}C NMR (101 MHz, $CDCl_3$) of product **P15**

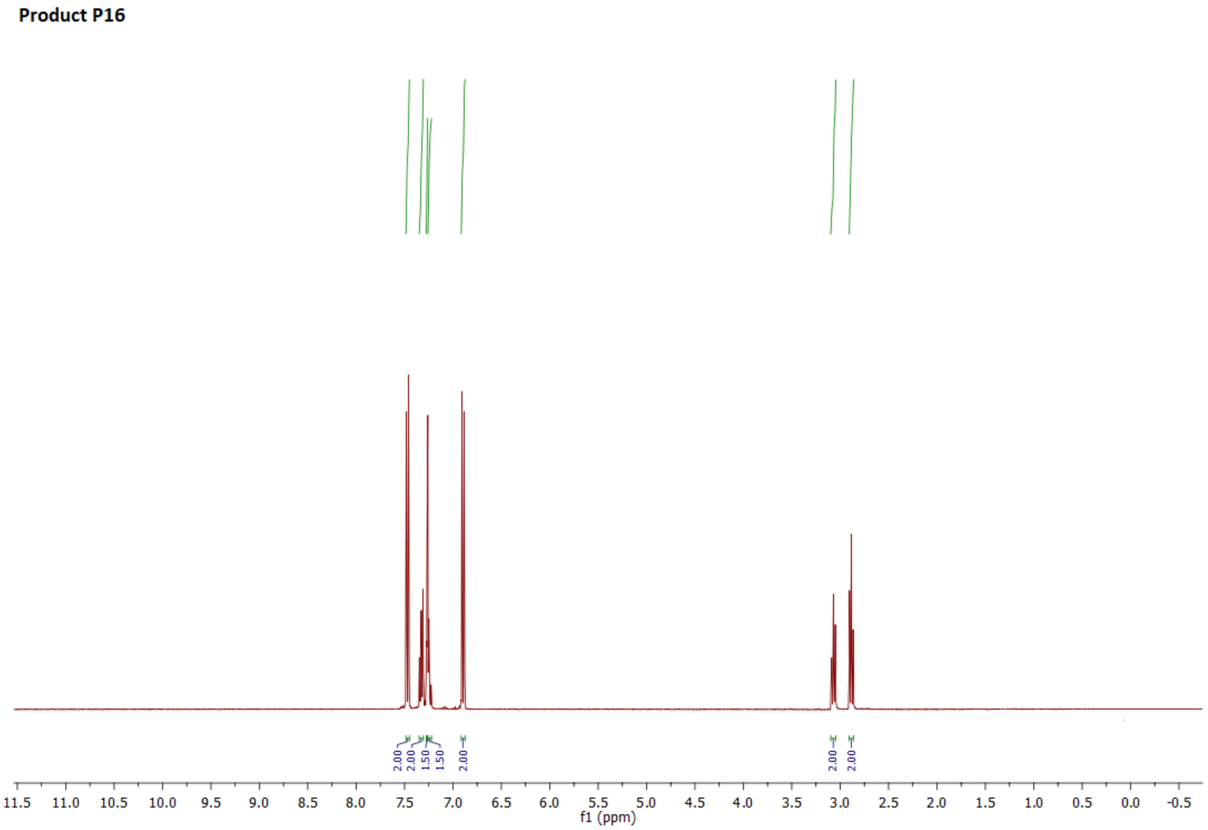

Figure S32. ^{1}H NMR (400 MHz, $CDCl_3$) of product **P16**

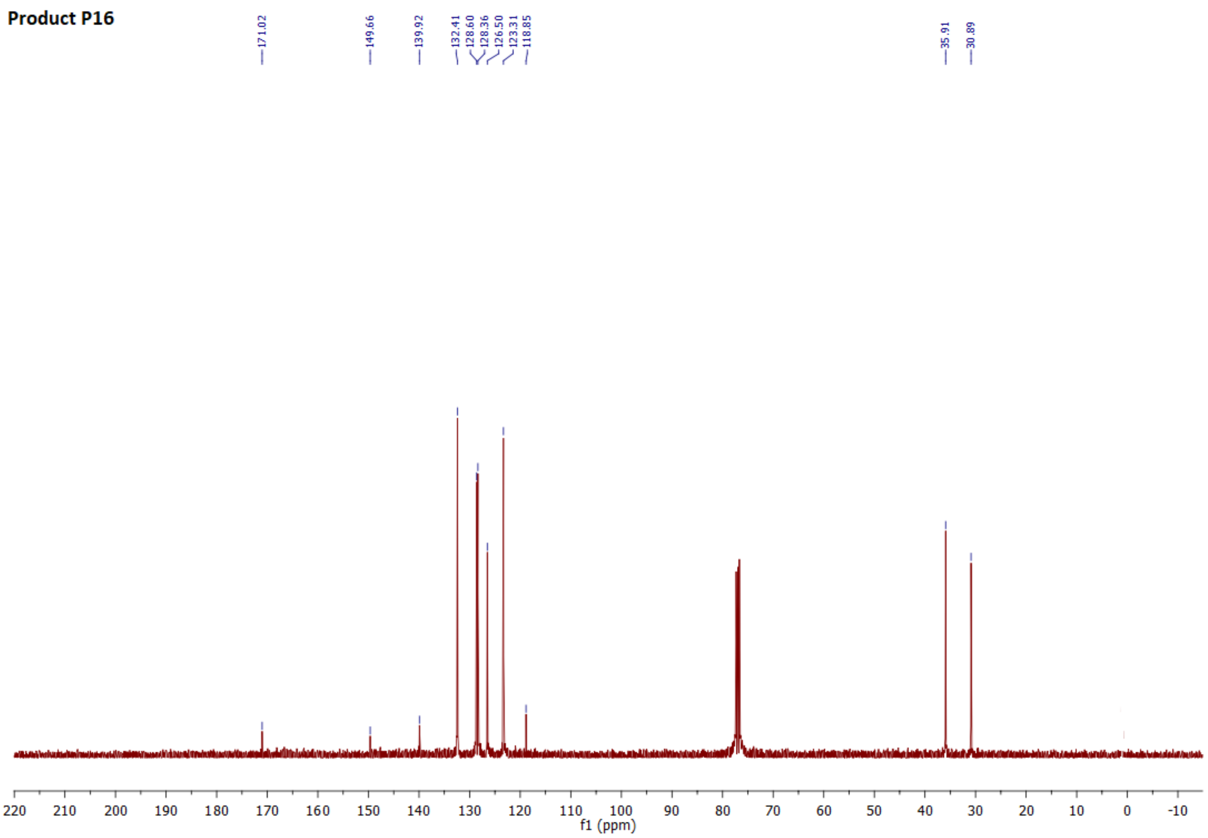

Figure S33. ^{13}C NMR (101 MHz, $CDCl_3$) of product **P16**

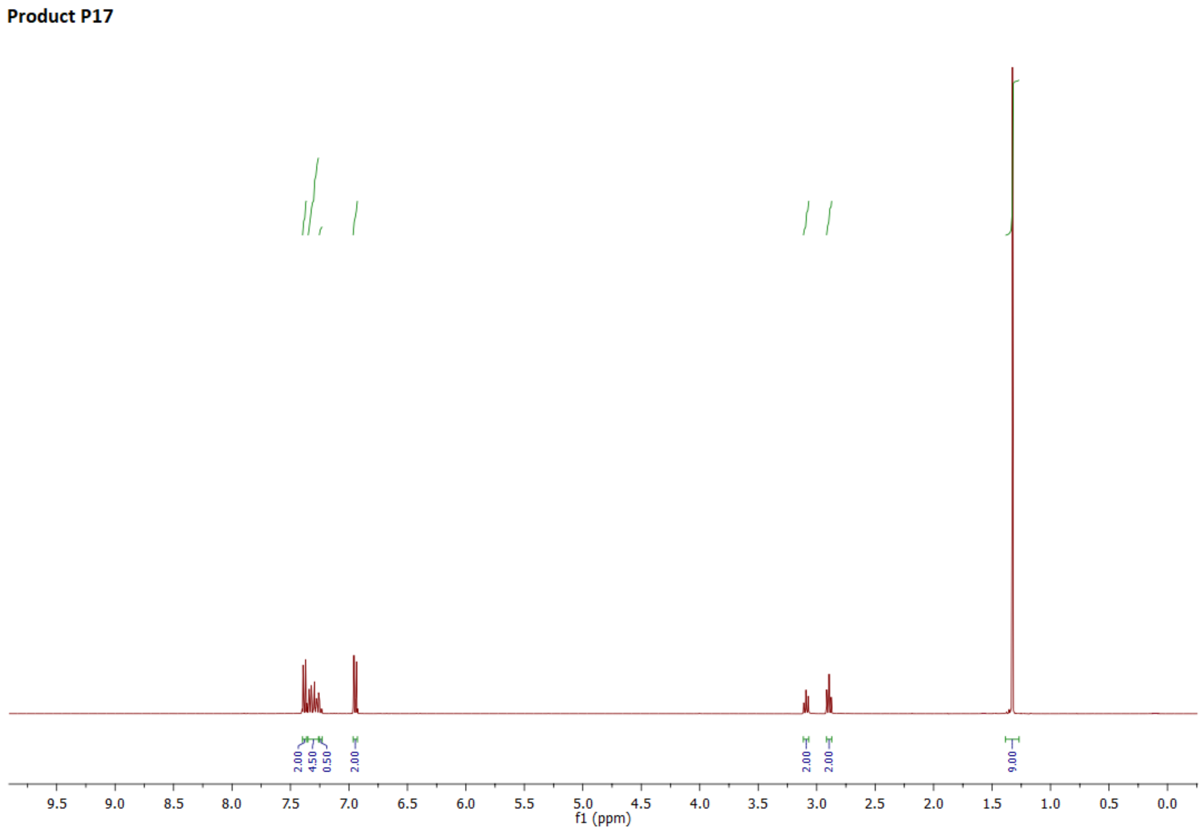

Figure S34. ^{1}H NMR (400 MHz, $CDCl_3$) of product **P17**

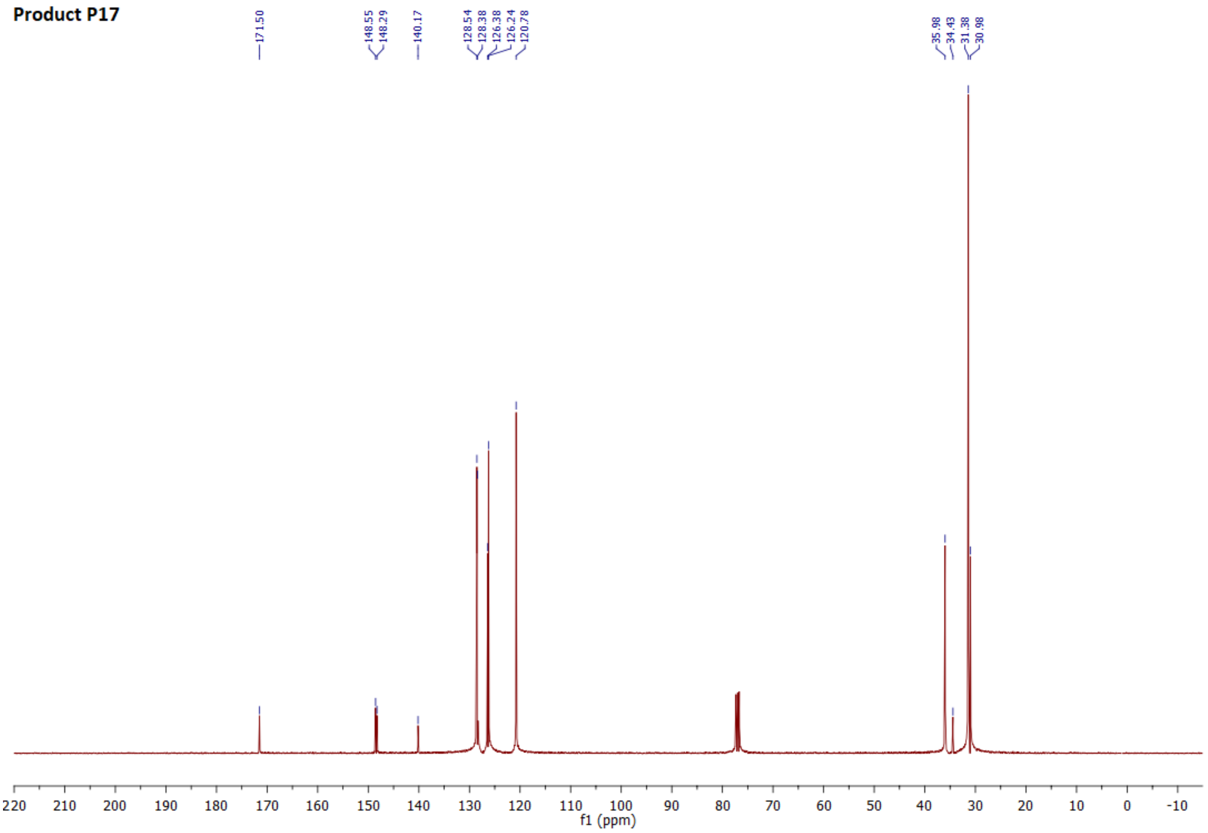

Figure S35. ^{13}C NMR (101 MHz, $CDCl_3$) of product **P17**

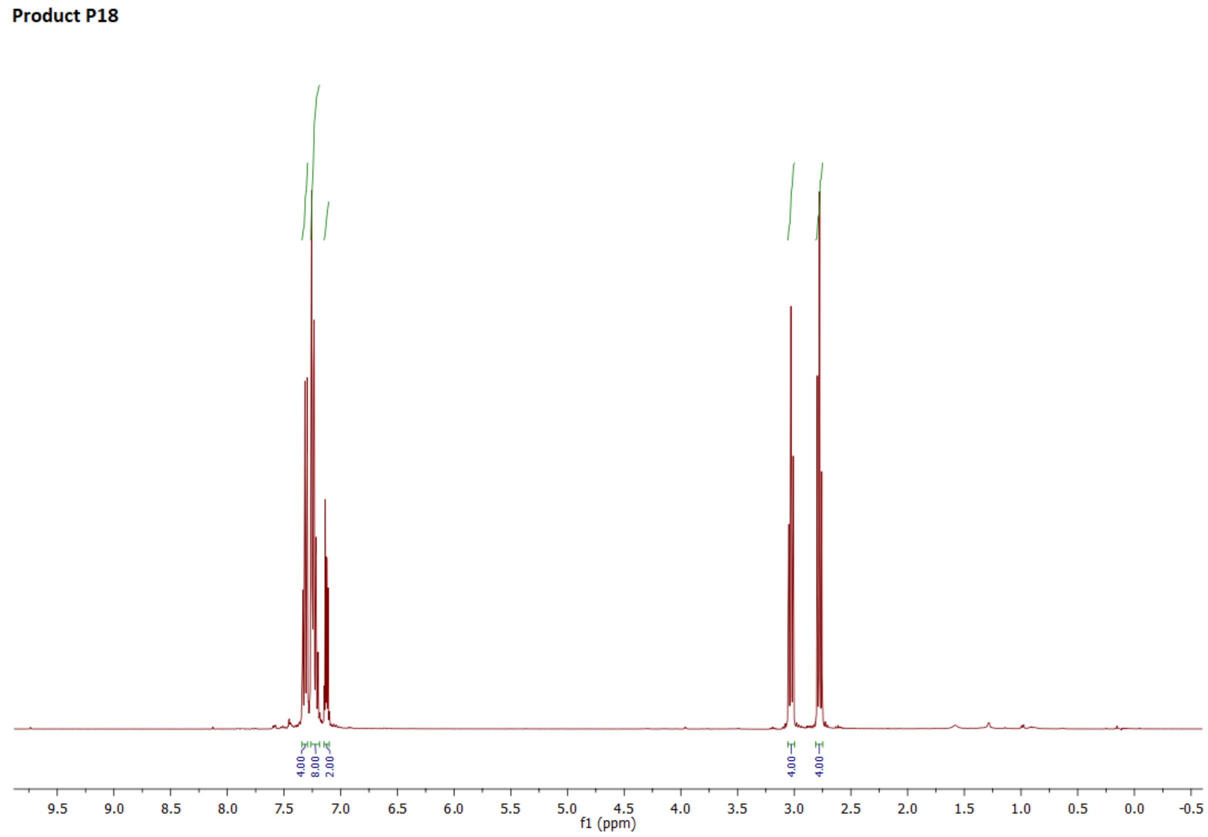

Figure S36. ^{1}H NMR (400 MHz, $CDCl_3$) of product **P18**

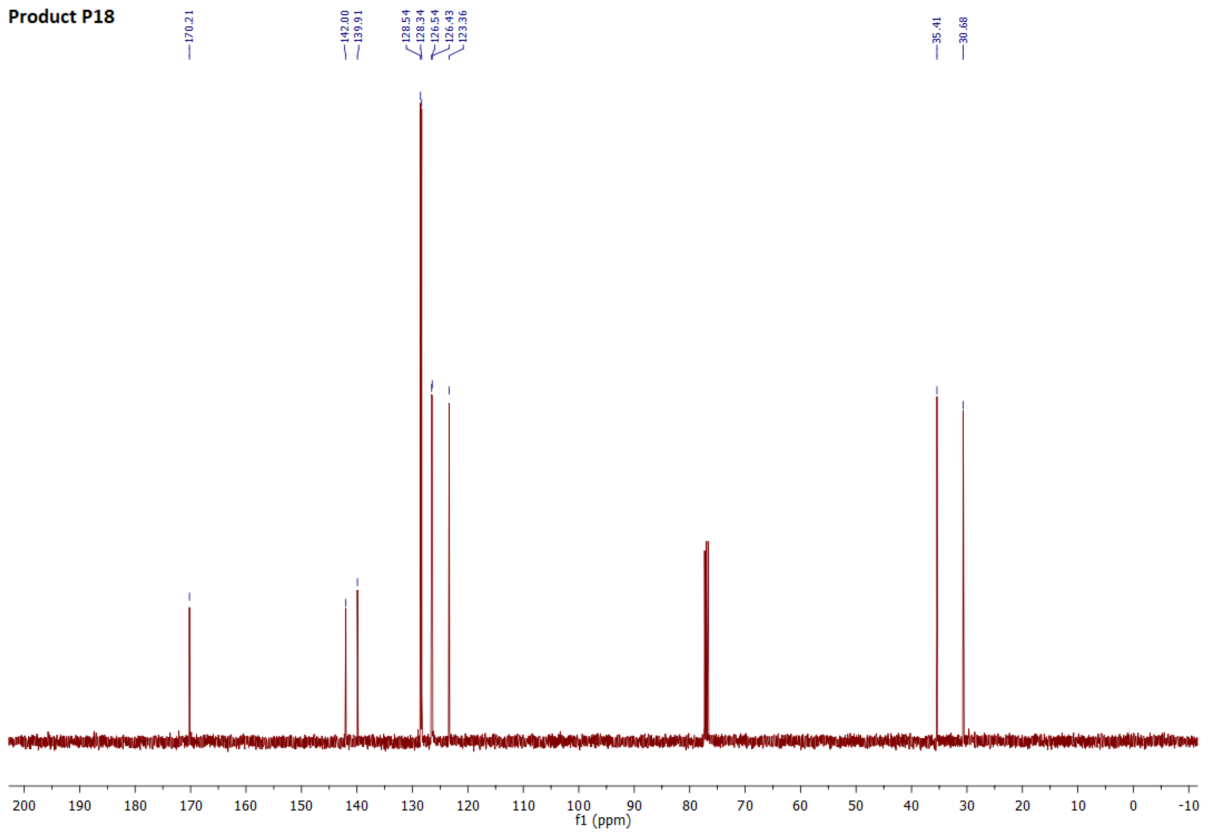

Figure S37. ^{13}C NMR (101 MHz, $CDCl_3$) of product **P18**

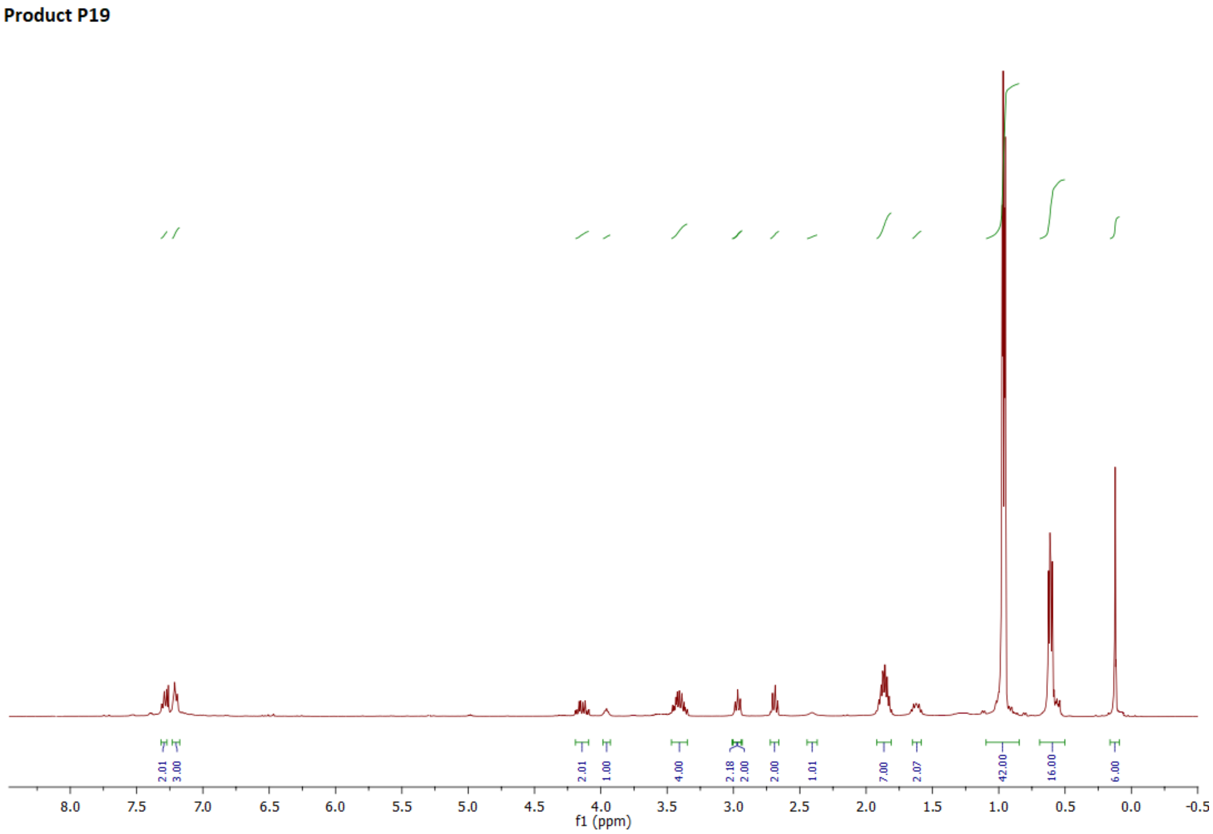

Figure S38. ^{1}H NMR (400 MHz, $CDCl_3$) of product **P19**

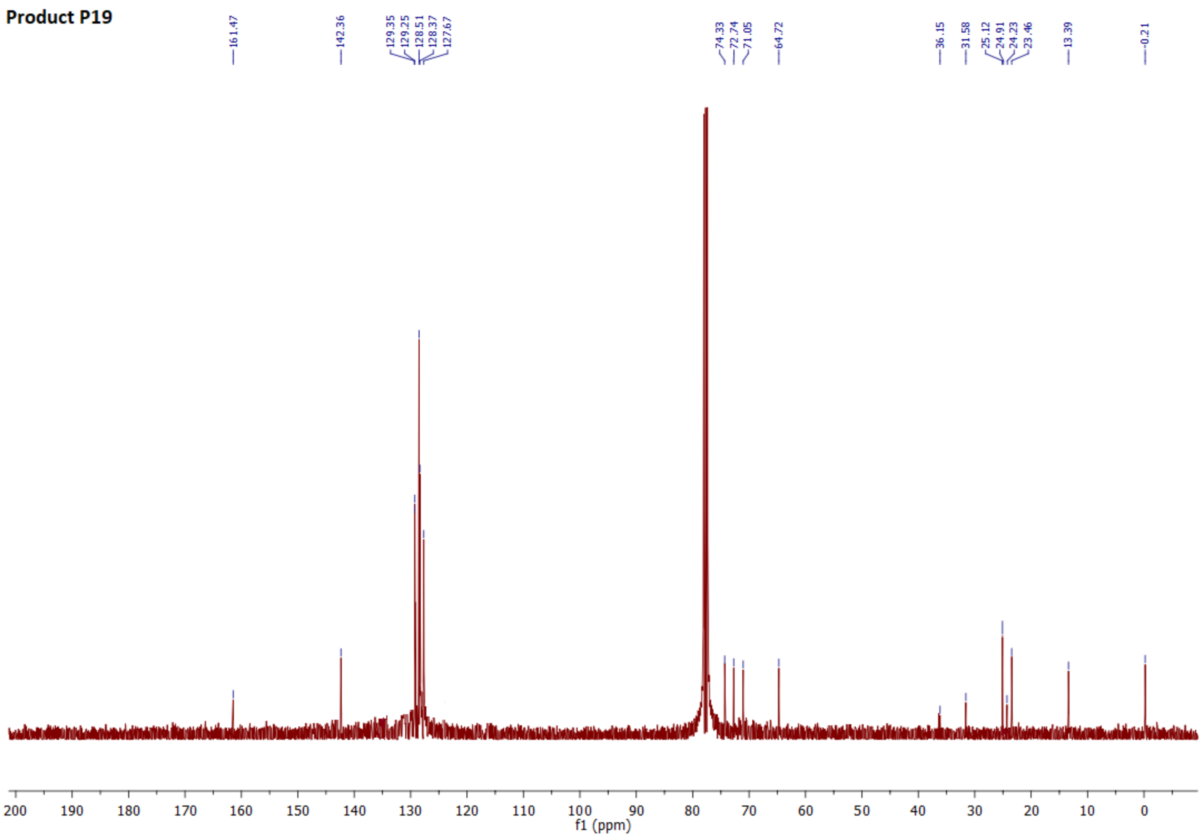

Figure S39. ^{13}C NMR (101 MHz, $CDCl_3$) of product **P19**

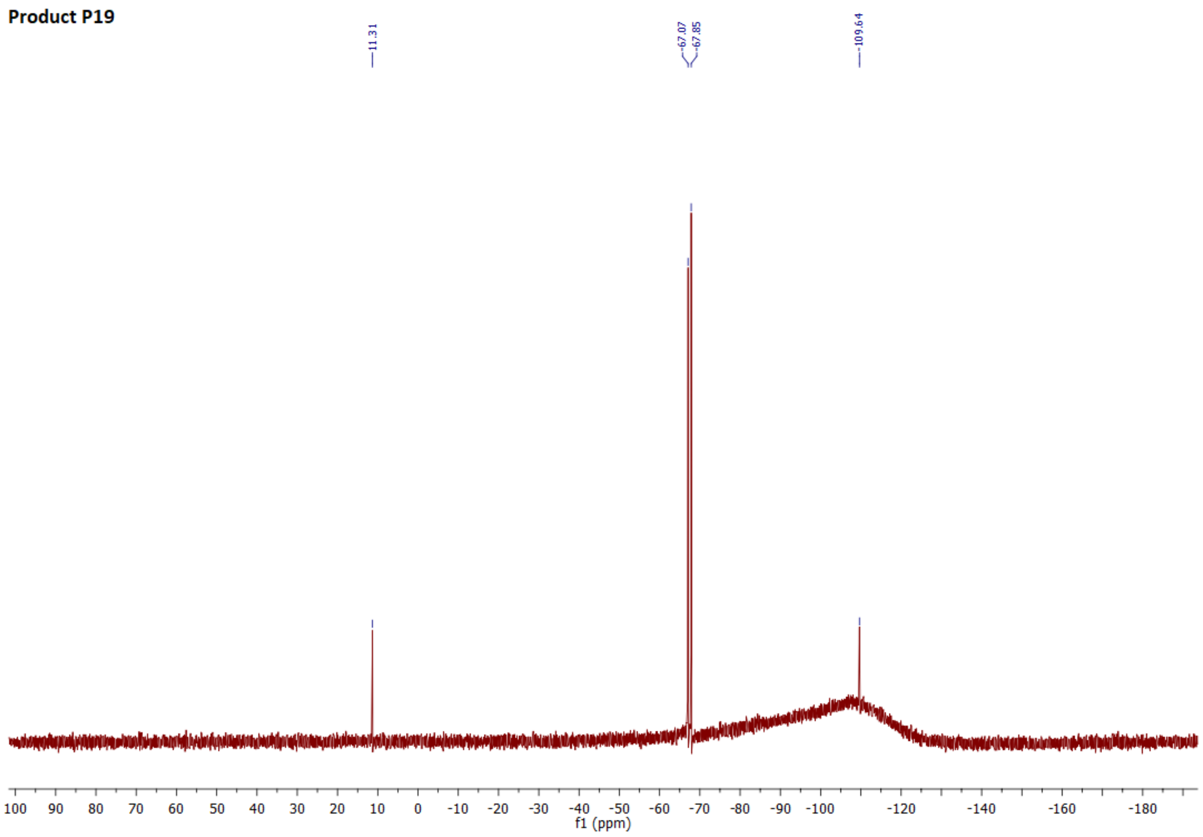

Figure S40. ^{29}Si NMR (79 MHz, $CDCl_3$) of product **P19**

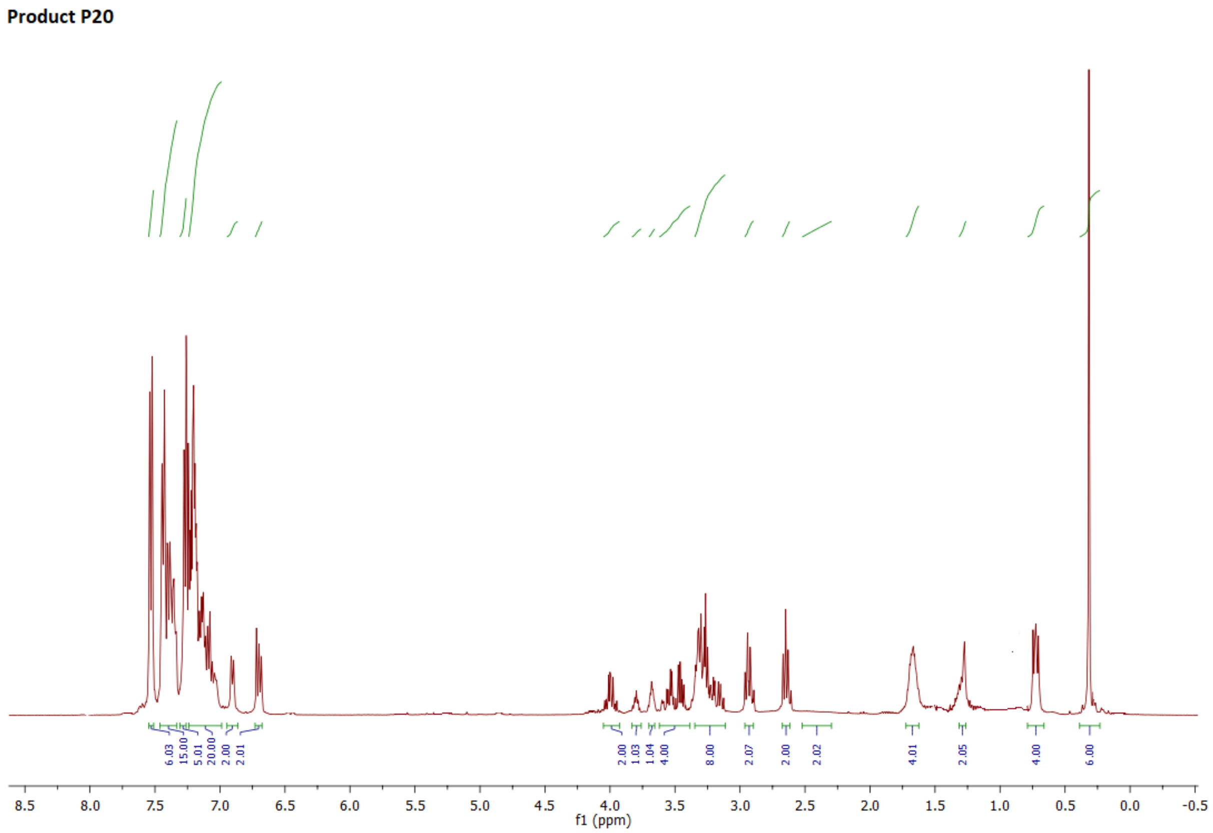

Figure S41. ^{1}H NMR (400 MHz, $CDCl_3$) of product **P20**

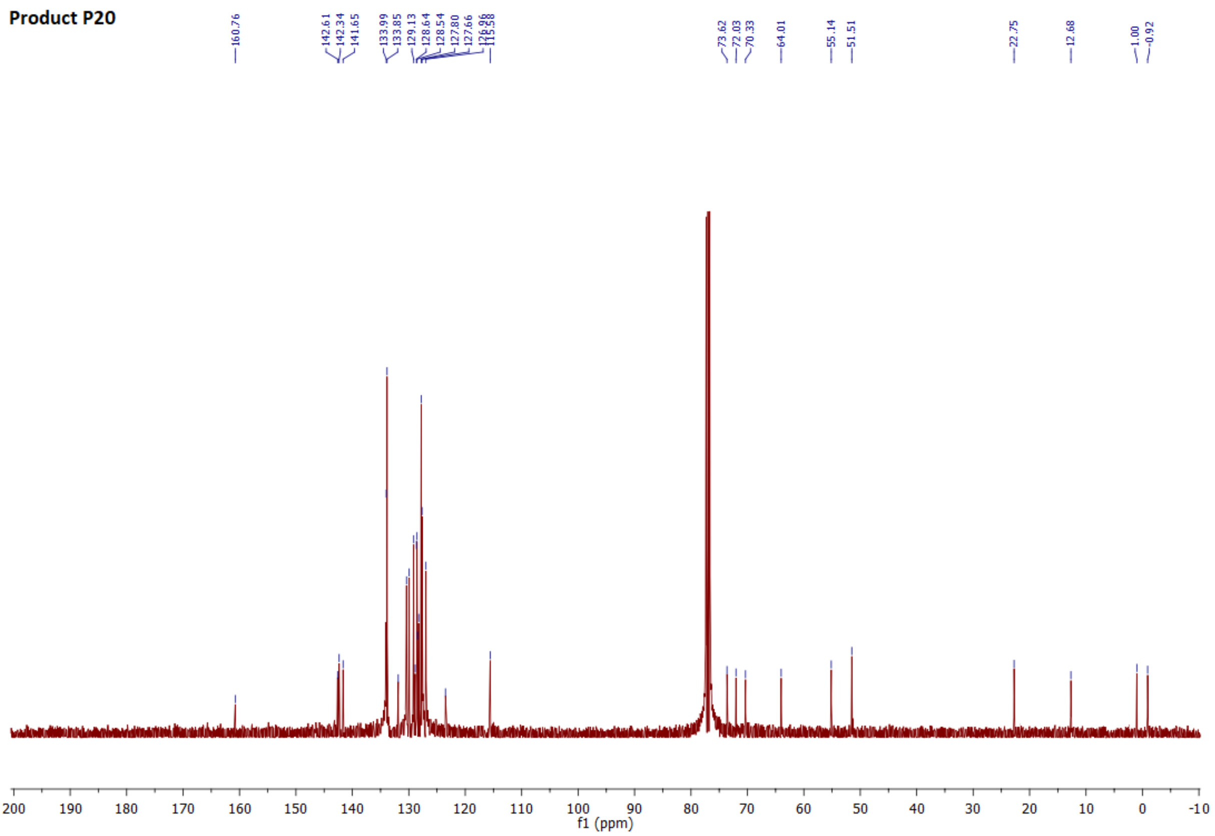

Figure S42. ^{13}C NMR (101 MHz, $CDCl_3$) of product **P20**

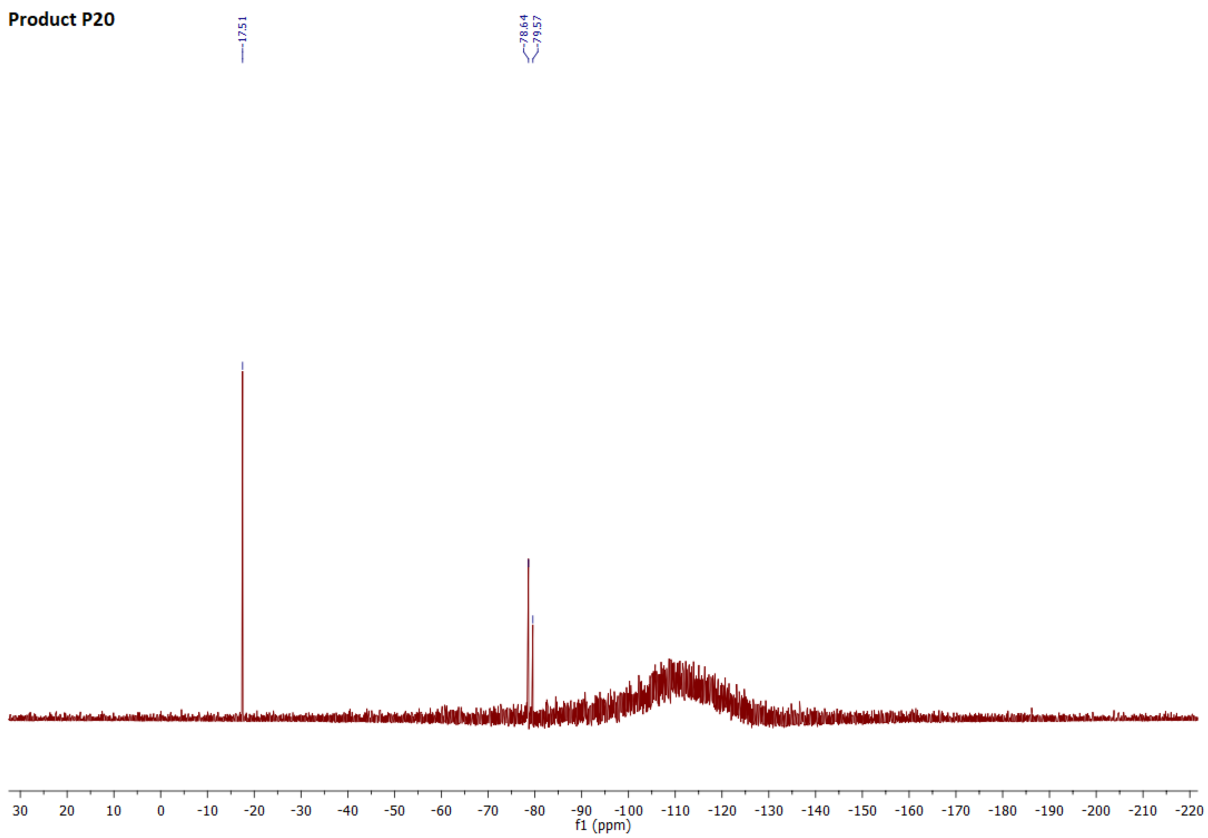

Figure S43. ^{29}Si NMR (79 MHz, $CDCl_3$) of product **P20**

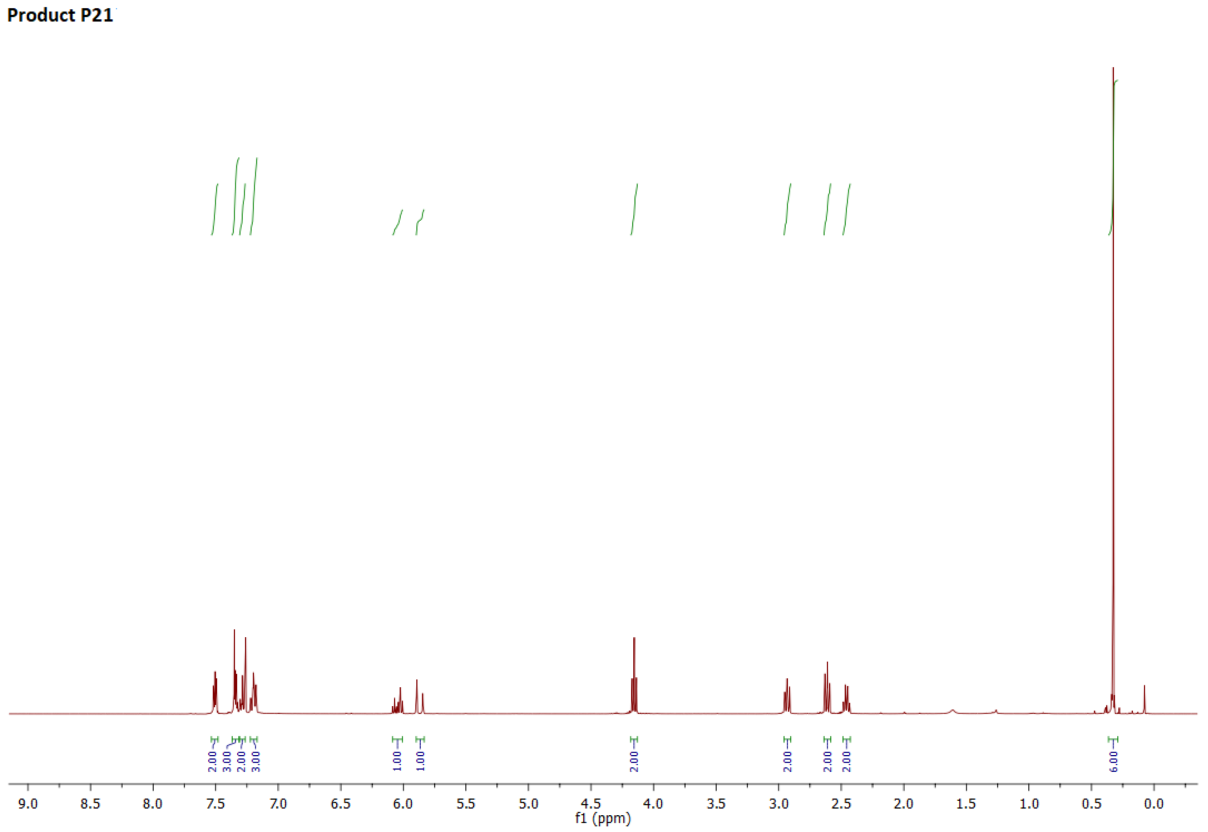

Figure S44. ^{1}H NMR (400 MHz, $CDCl_3$) of product **P21**

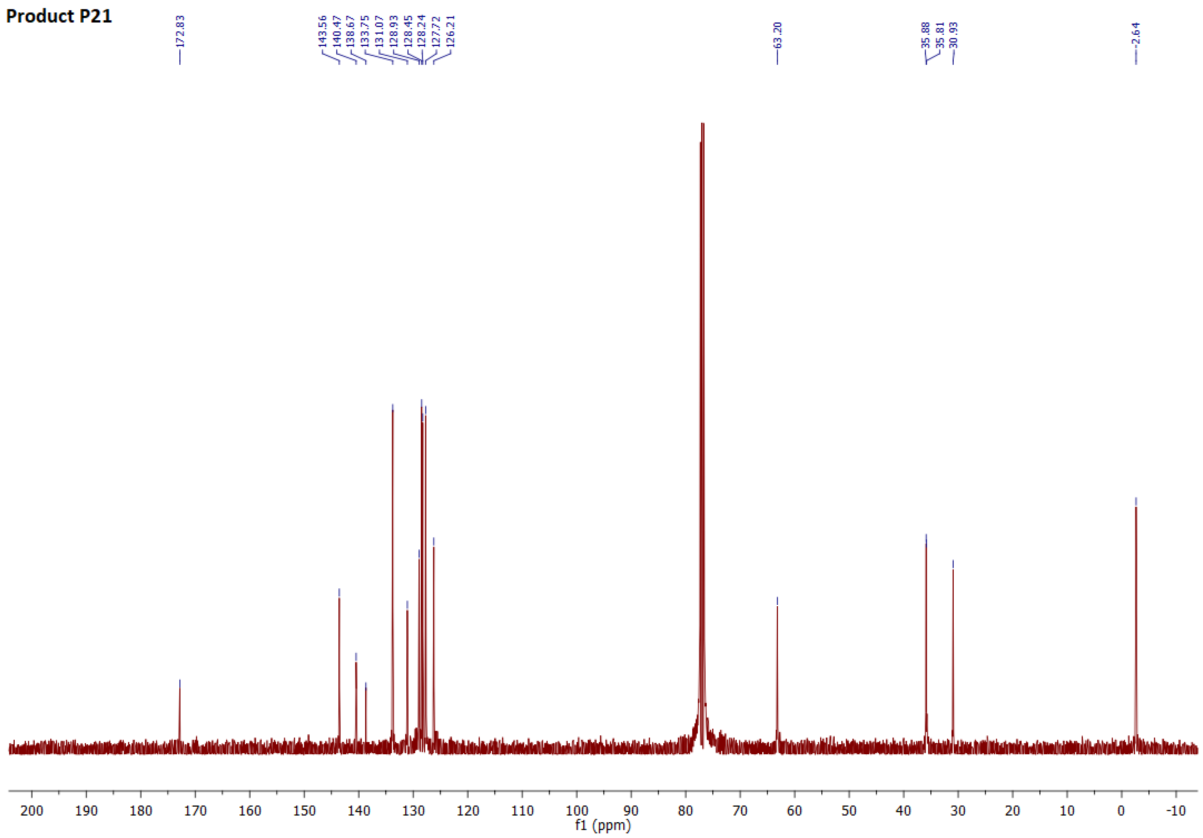

Figure S45. ^{13}C NMR (101 MHz, $CDCl_3$) of product **P21**

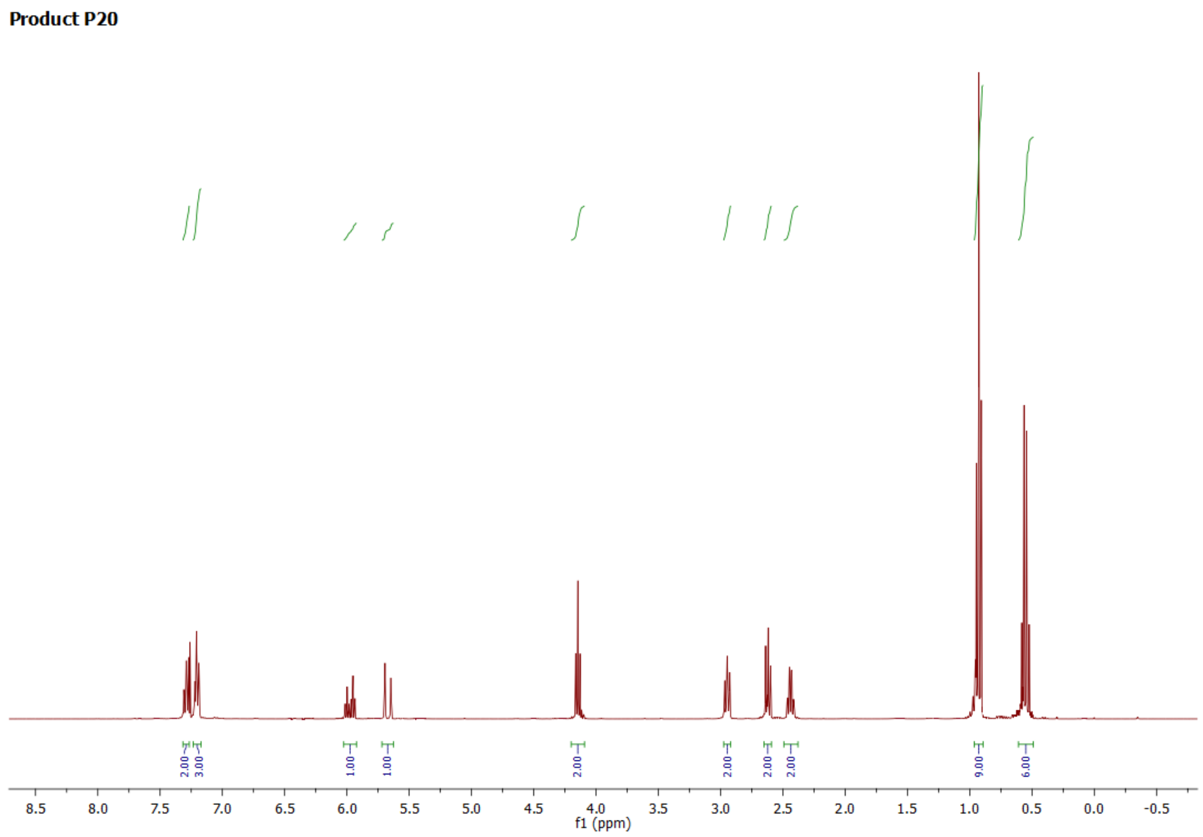

Figure S46. ^{1}H NMR (400 MHz, $CDCl_3$) of product **P22**

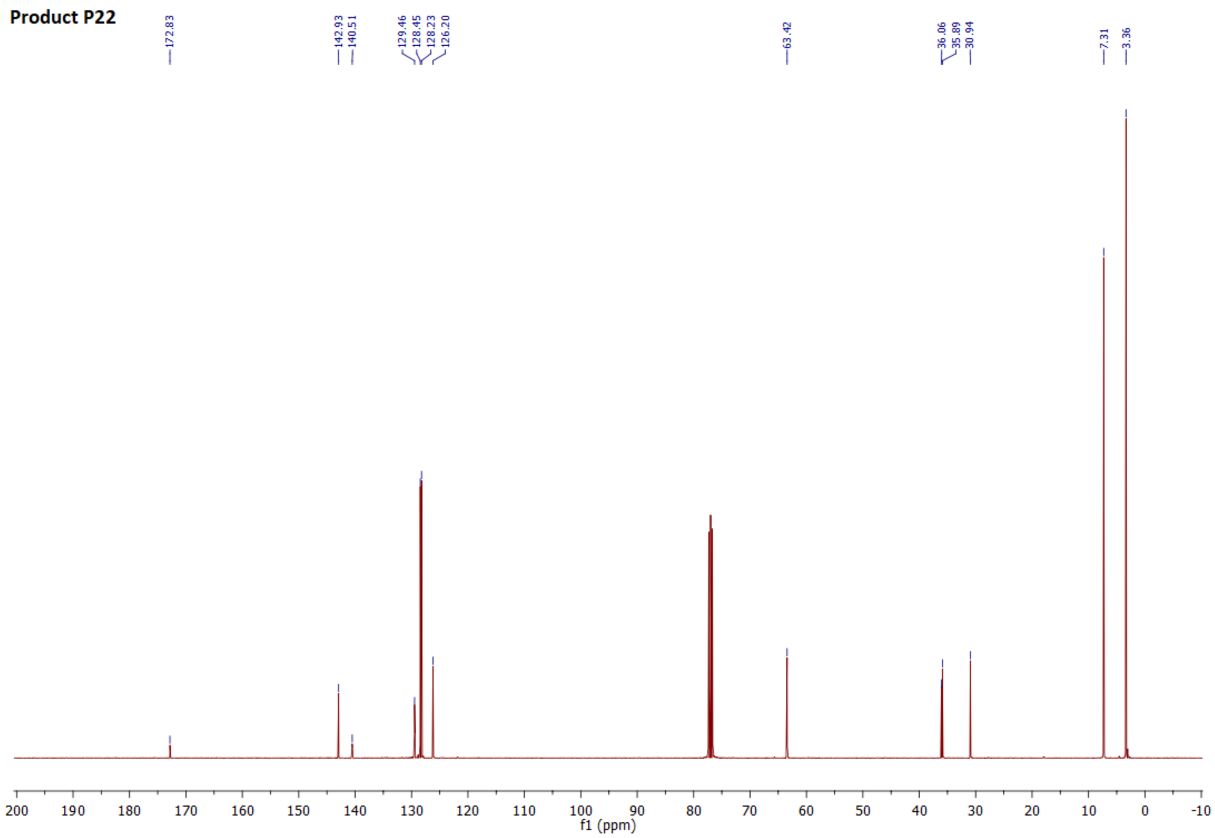

Figure S47. ^{13}C NMR (101 MHz, $CDCl_3$) of product **P22**

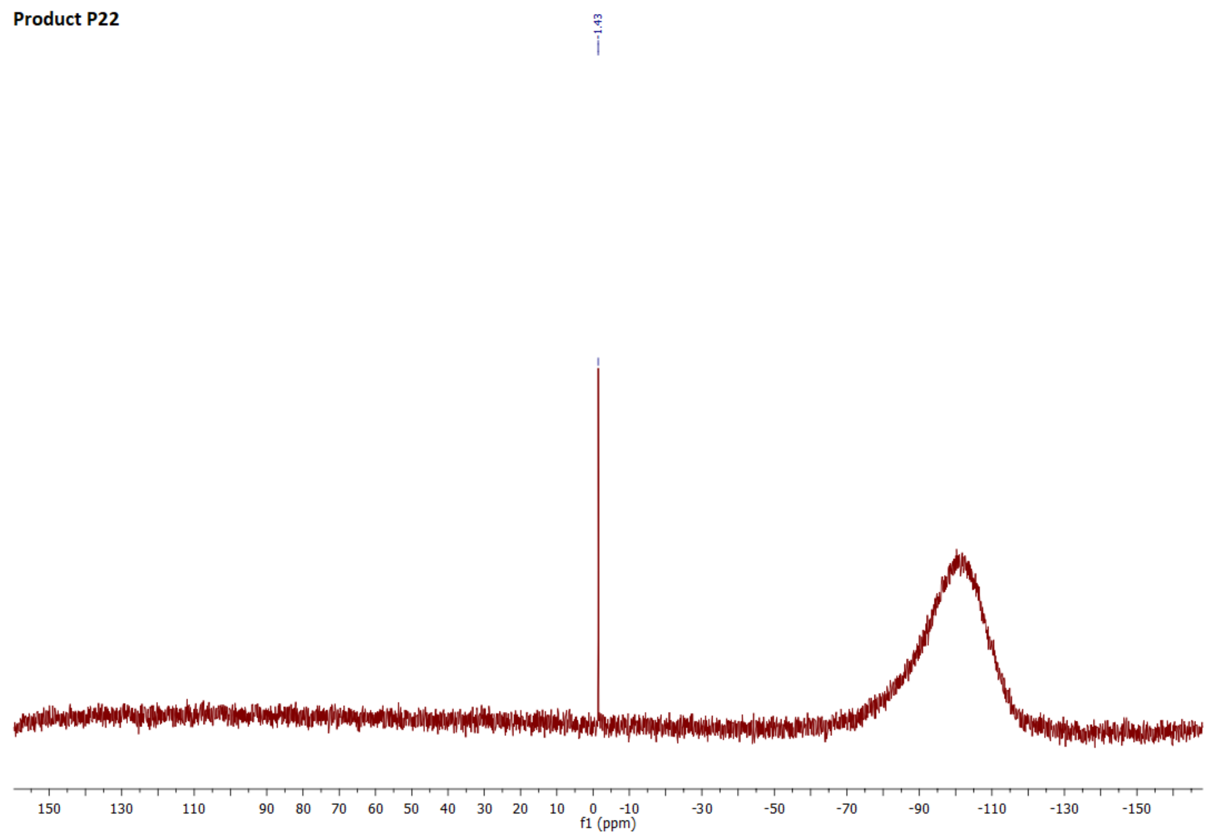

Figure S48. ^{29}Si NMR (79 MHz, $CDCl_3$) of product **P22**

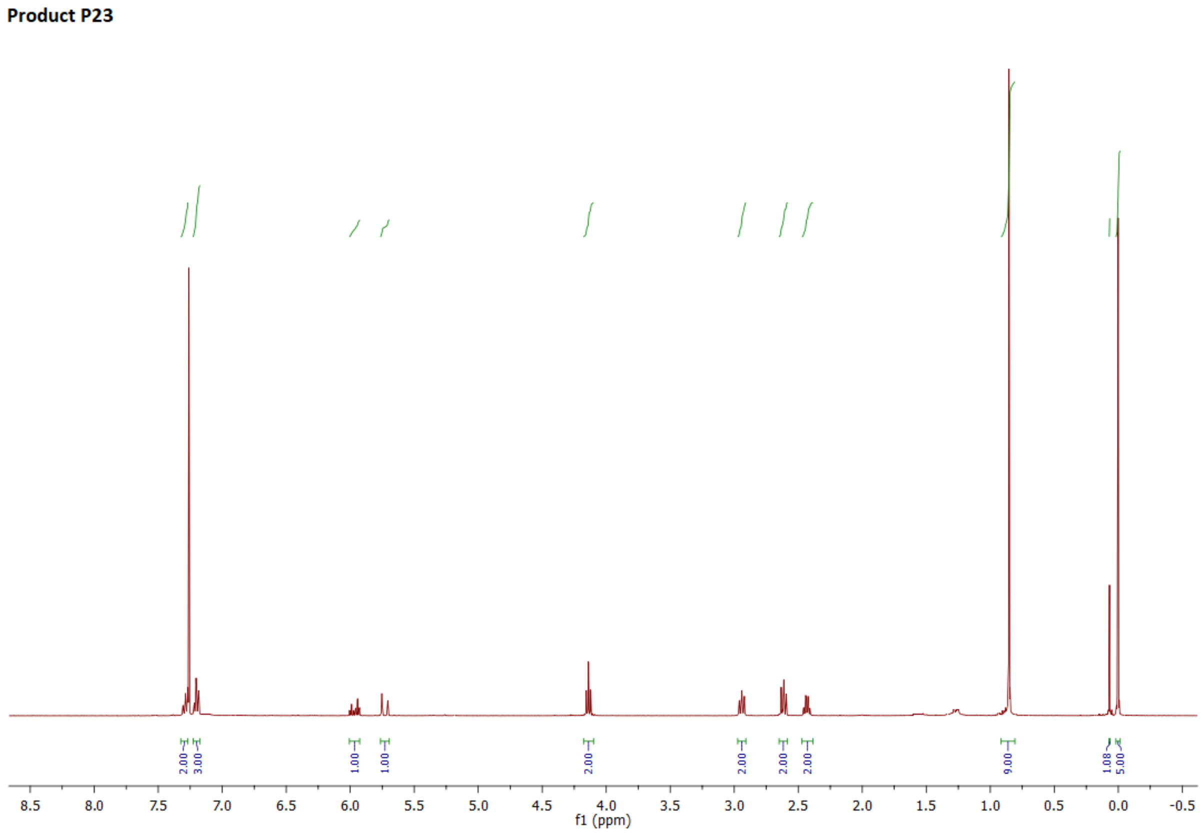

Figure S49. ^{1}H NMR (400 MHz, $CDCl_3$) of product **P23**

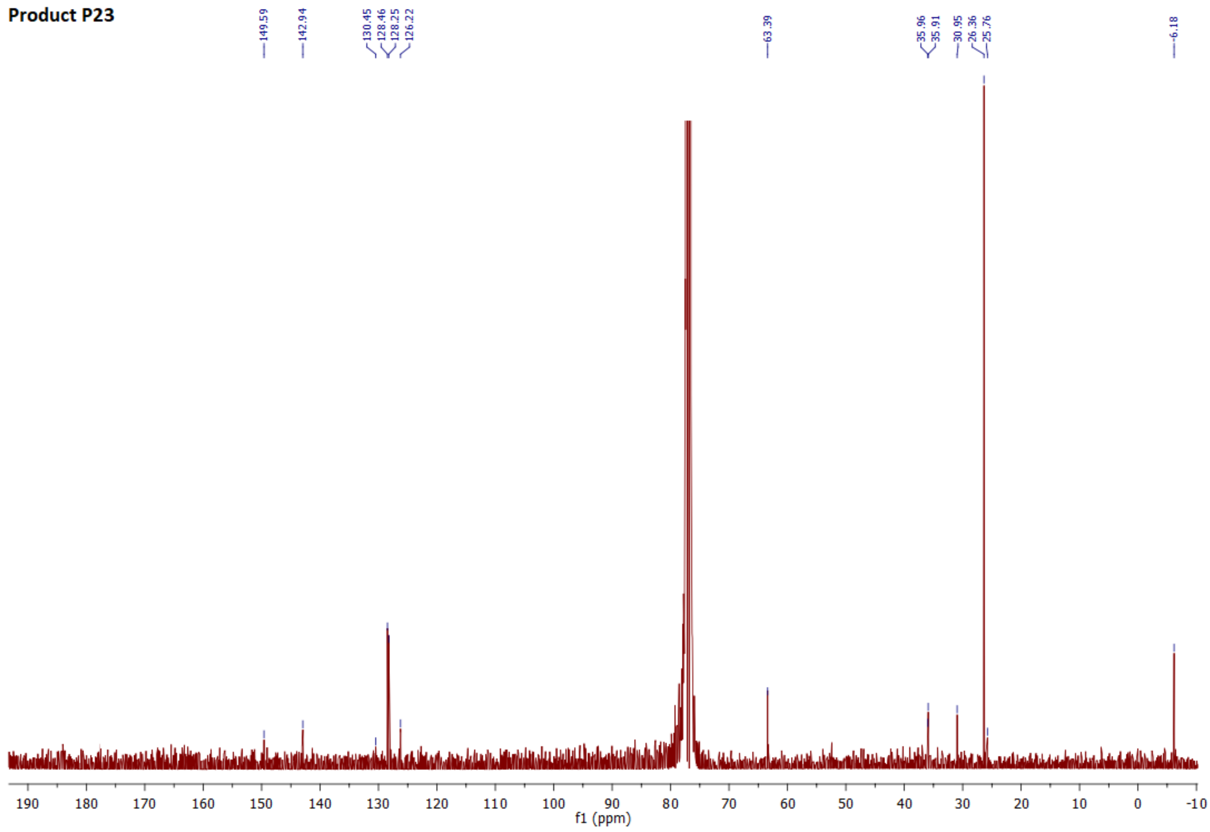

Figure S50. ^{13}C NMR (101 MHz, $CDCl_3$) of product **P23**

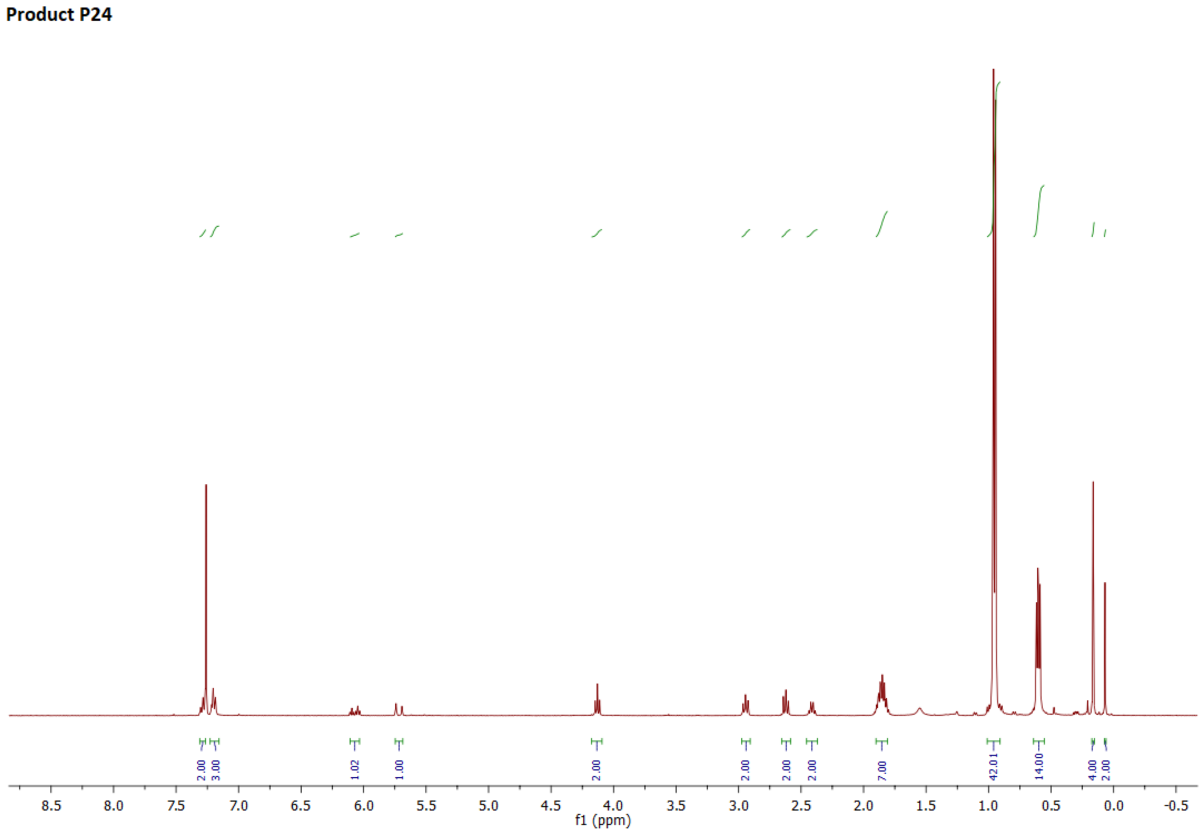

Figure S51. ^{1}H NMR (400 MHz, $CDCl_3$) of product **P24**

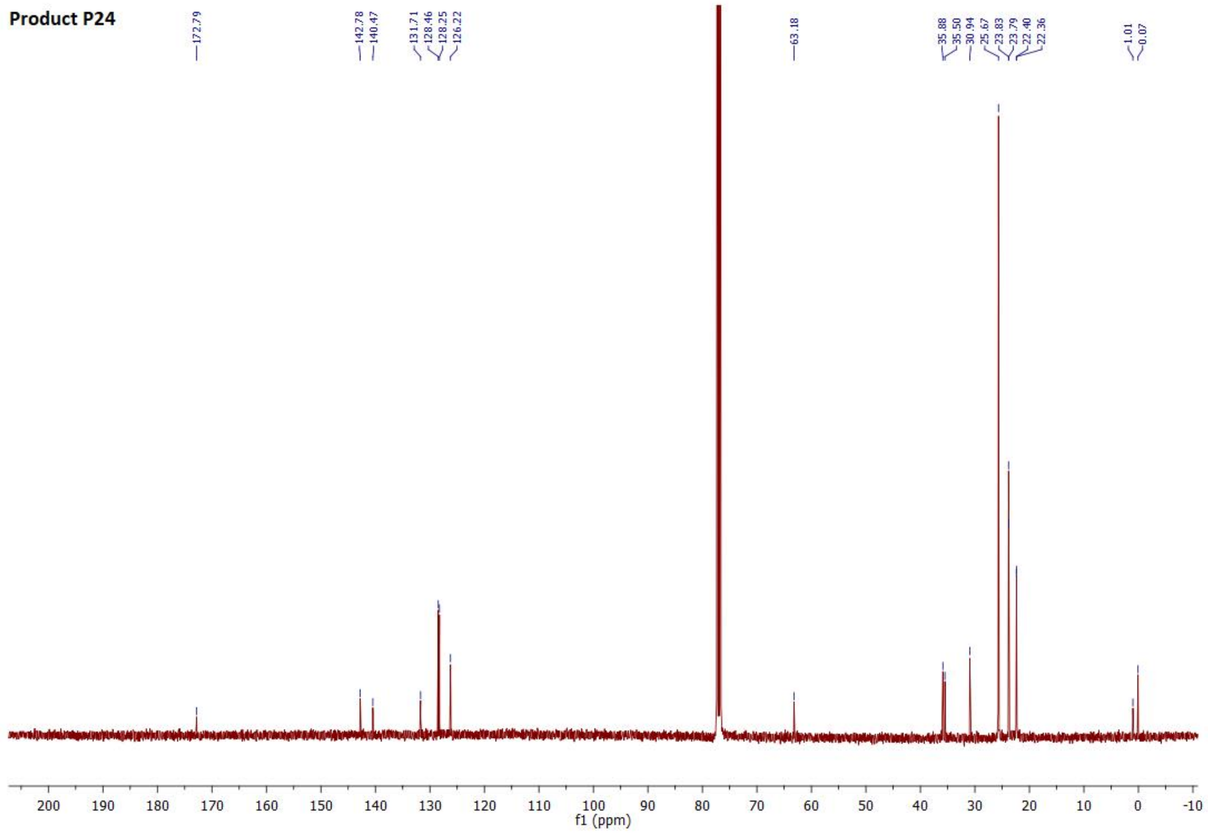

Figure S52. ^{13}C NMR (101 MHz, $CDCl_3$) of product **P24**

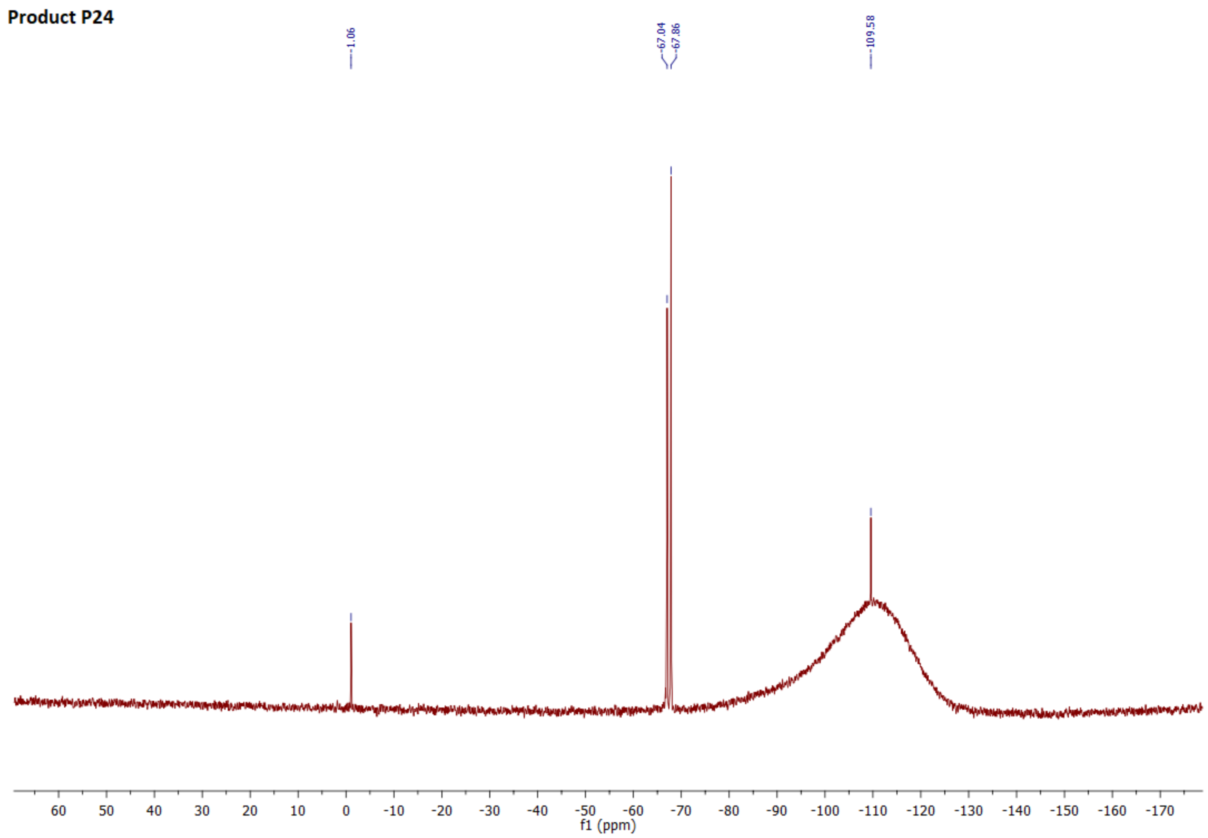

Figure S53. ^{29}Si NMR (79 MHz, $CDCl_3$) of product **P24**

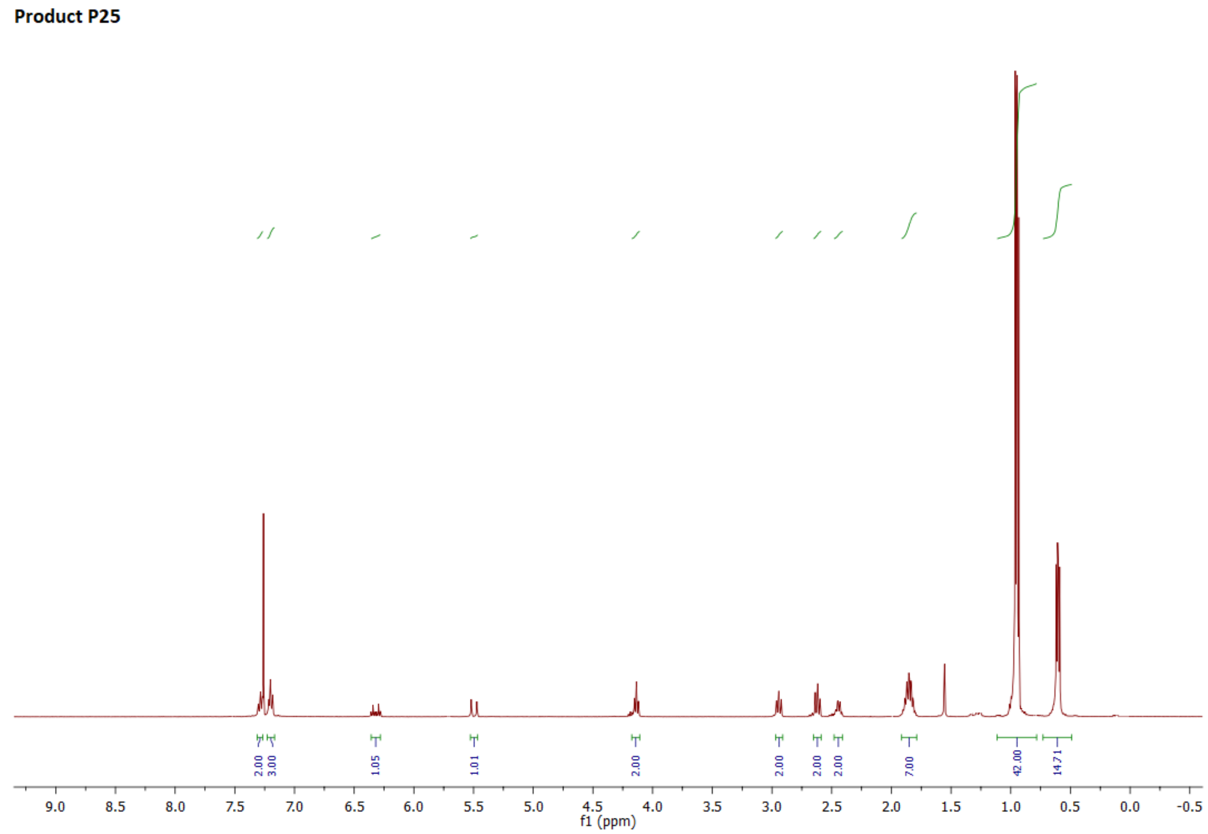

Figure S54. ^{1}H NMR (400 MHz, $CDCl_3$) of product **P25**

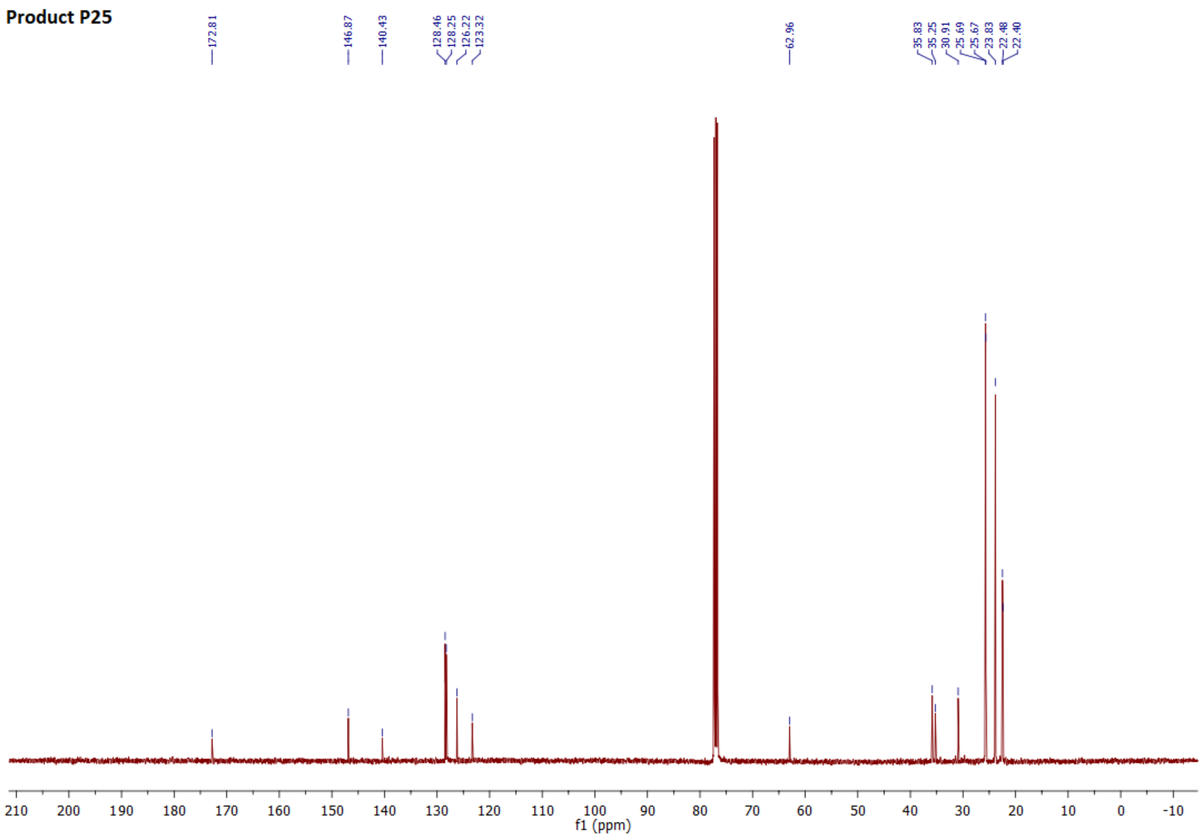

Figure S56. ^{13}C NMR (101 MHz, $CDCl_3$) of product **P25**

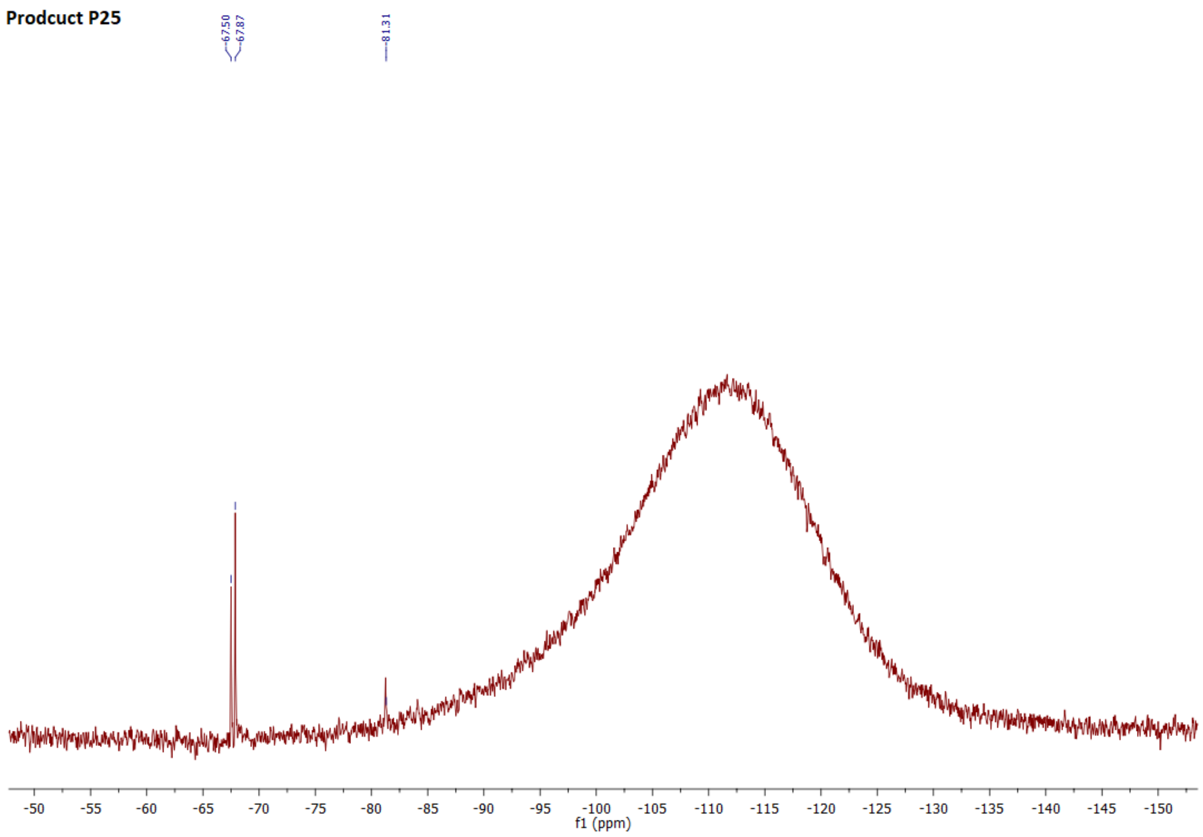

Figure S57. ^{29}Si NMR (79 MHz, $CDCl_3$) of product **P25**

5. References

[1] A. Chan, K. A. Scheidt, *Org. Lett*., **2005,** *7*, 5, 905–908.

[2] S. Zhibin; Z. Youkang; T. Duanjian, Patent Application, CN109678718A, **2019**.

[3] M. Shuang, P. H. Toy., *Molecules*, **2016**, *21*, 1-12.

[4] P. E. Peterson, M. Stepanian, *J. Org. Chem*., **1988**, *53*, 1903- 1907.

[5] H. Chen, DH.Chen, PQ. Huang, *Sci. China Chem.*, **2020**, *63*, 370–376.

[6] N. Kurono, K. Sugita, S. Takasugi, M. Tokuda, *Tetrahedron*, **1999**, *55*, 6097-6108.

[7] I. A. Rivero, S. Heredia, A. Ochoa, *Synth. Commun*., **2001**, *31*, 2169-2176.

[8] V. Singhania, M. Cortes-Clerget, J. Dussart-Gautheret, B. Akkachairin, J. Yu, N. Akporji, B. H. Lipshutz, *Chem. Sci.*, **2022**, *13*, 1440-1445.
